# The Conformational Dynamics of the Ligands Determines the Electronic Circular Dichroism of the Chiral Au_38_(SC_2_H_4_Ph)_24_ Cluster

**DOI:** 10.1021/acs.jpclett.2c03923

**Published:** 2023-02-14

**Authors:** M. Monti, G. Brancolini, E. Coccia, D. Toffoli, A. Fortunelli, S. Corni, M. Aschi, M. Stener

**Affiliations:** † Dipartimento di Scienze Chimiche e Farmaceutiche, Università di Trieste, Via L. Giorgieri 1, 34127 Trieste, Italy; ‡ Istituto Nanoscienze, CNR-NANO, Center S3, Via G. Campi 213/A, 41100 Modena, Italy; § CNR-ICCOM, Consiglio Nazionale delle Ricerche, via G. Moruzzi 1, 56124, Pisa, Italy; ∥ Dipartimento di Scienze Chimiche, Università di Padova, Via Francesco Marzolo 1, 35131 Padova, Italy; ⊥ Dipartimento di Scienze Fisiche e Chimiche, Università dell’Aquila, Via Vetoio, 67100, l’Aquila, Italy

## Abstract

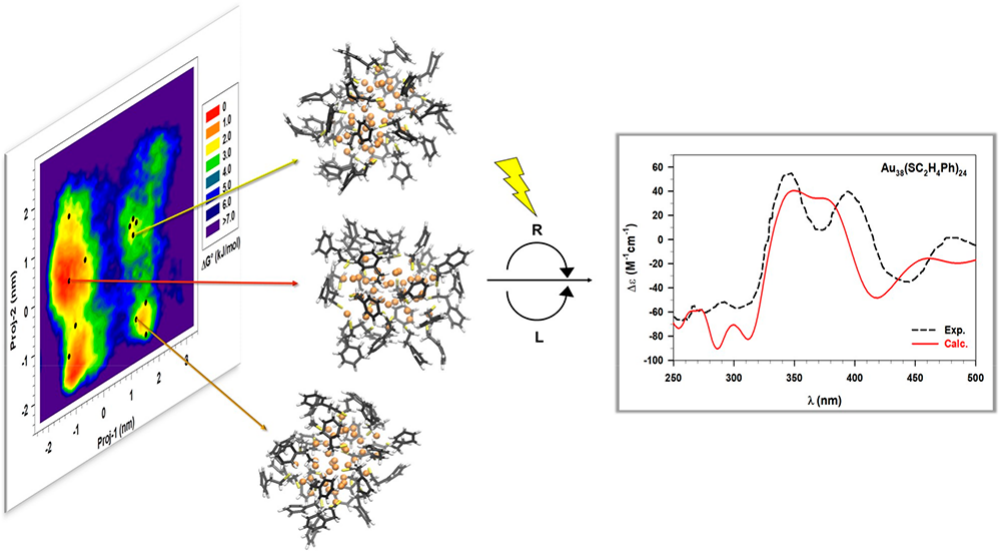

Effects of the conformational dynamics of 2-PET protective ligands on the electronic circular dichroism (ECD) of the chiral Au_38_(SC_2_H_4_Ph)_24_ cluster are investigated. We adopt a computational protocol in which ECD spectra are calculated via the first principle polTDDFT approach on a series of conformations extracted from MD simulations by using Essential Dynamics (ED) analysis, and then properly weighted to predict the final spectrum. We find that the experimental spectral features are well reproduced, whereas significant discrepancies arise when the spectrum is calculated using the experimental X-ray structure. This result unambiguously demonstrates the need to account for the conformational effects in the ECD modeling of chiral nanoclusters. The present procedure proved to be able of capturing the essential conformational features of the dynamic Au_38_(SC_2_H_4_Ph)_24_ system, opening the possibility to model the ECD of soluble chiral nanoclusters in a realistic way.

Chiral thiolate-protected gold nanoclusters (RS-AuNCs) have attracted much attention in recent years because of their several potential applications in different fields, such as chiral catalysis,^1,2^ sensing and recognition,^3,4^ as well as chiral separation for biological molecules.^5^ In general, the correlation between the cluster structure and its optical properties is a fundamental issue to allow rational design for potential applications.^6^ Of particular interest in these systems is the electronic circular dichroism (ECD) response beyond the spectral region due to the ligands themselves that generally lies in the UV range, i.e., the metal-based region of optical absorption.^7^ An important question concerns the origin of the chiroptical properties of RS-AuNCs, and several mechanisms have been debated over the years to explain and rationalize it. In detail, either the metal core has an asymmetric arrangement which determines an intrinsic chirality,^8^ or the chiral response can be induced by the presence of the protective ligands. In the latter case, we can consider: (i) achiral ligands that form chiral adsorption patterns (footprint model)^9^ and/or (ii) chiral ligands that induce the chirality in the metal core by trapping its electrons in a dissymmetric electric field.^10^

For instance, the chirality of the Au_38_(SC_2_H_4_Ph)_24_ cluster (SC_2_H_4_Ph abbreviated as 2-PET from now on), which is the objective of this study, is induced by the passivation of achiral 2-PET ligands on the surface of the metallic core. This nanocluster has attracted research interest not only on fundamental studies of chirality,^11^ but also on stability,^12^ doping,^13^ and catalysis,^14^ especially after 2010 when its total crystal structure was resolved by Qian et al.^15^ Moreover, the first characterization of the 8 kDa cluster by Schaaff et al.^16^ in 1997, well before the determination of its atomistic structure, inspired many theoretical works to examine structural and electronic properties of the Au_38_(SR)_24_ nanocluster.^17−19^ After a decade of density-functional theory (DFT) predictions, both experiment^15^ and theory^20,21^ confirmed that the Au_38_(2-PET)_24_ shape is prolate with a face-fused bi-icosahedral Au_23_ core protected by 3 short Au(SR)_2_ and 6 long Au_2_(SR)_3_ staples. The Au_23_ core, although slightly distorted, can be associated with a *D*_3*h*_ symmetry that is lowered to a *D*_3_ one by the Au–S staple motifs, which assume a chiral arrangement. Indeed, the 6 long staples are divided in 2 triblade fans with a staggered configuration that rotate clockwise or anticlockwise depending on the enantiomer considered (see Figure 1). The 3 short staples instead slightly tilt with respect to the 3-fold axis and follow the handedness of the Au_2_(SR)_3_ staples.

**Figure 1:**
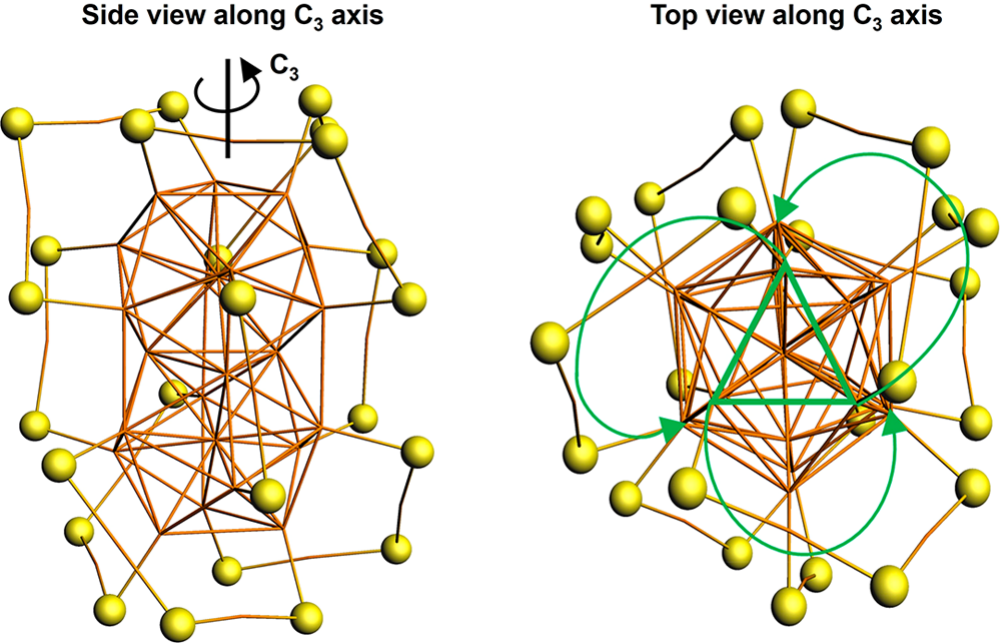
Side (left) and top (right) view along the *C*_3_ axis of the gold–sulfur architecture. For clarity the organic ligands were removed, while the Au and S atoms are reported as sticks in orange and balls in yellow, respectively. The (left-)handedness of the cluster studied in this work is modeled by the green arrows that connect the core Au atoms (green triangle) with the inner Au atoms of the *star-shaped* staples.

Despite many studies on the structure and chirality of Au_38_(2-PET)_24_, the measurement of the ECD was revealed to be very challenging because of the inability to separate the two enantiomers in solution. Such limitation was finally overcome in 2012 when Dolamic et al.^22^ performed the separation of the racemic mixture employing chiral high-performance liquid chromatography. The ECD of the left-handed enantiomer was proposed again in 2014 by Barrabés and co-workers^23^ together with the spectra of Pd_2_Au_36_(2-PET)_24_ clusters to investigate the doping effects on the chiroptical properties. Independently, in the same year Xu et al.^7^ proposed an enantioselective synthesis of several Au_38_(SR)_24_ nanoclusters by using chiral ligands such as 2-phenylpropane-1-thiol. The ECD spectra recorded strongly resemble those measured by Dolamic and co-workers^22^ for the Au_38_(2-PET)_24_ enantiomers.

From a theoretical perspective, the availability of experimental ECD spectra together with the resolution of several X-ray structures represented a huge step forward for the investigation of the RS-AuNCs chirality and the assignment of their absolute configurations. Focusing on Au_38_ NCs, ECD calculations have been already performed for instance by Lopez-Acevedo et al.^21^ or by Baseggio and co-workers^24^ using time-dependent density functional theory (TDDFT) and different thiolate protective ligands. However, theoretical approaches for modeling the ECD of nanoclusters have so far mostly neglected a fundamental aspect of the physics of this system, i.e., the mobility of the ligands and thus its influence on the chiroptical response. To the best of our knowledge only one previous explorative study has highlighted the role of the different conformations of the ligands on the ECD of the RS-AuNCs.^25^ Indeed, the ECD calculation is typically performed on the experimental X-ray structure in a vacuum to overcome the computational costs and difficulties of conformational and response predictions. While the metal and Au–S interface structures are expected to be preserved in solution, significant changes can instead occur in the ligands, so that the neglect of these conformational effects can produce discrepancies between experiment and theory.^24^ In recent years, the dynamic effects of RS-AuNCs have been investigated in several works of Bürgi and collaborators^26−28^ combining vibrational spectroscopy and theoretical calculations (i.e., DFT and molecular dynamics (MD) simulations). Furthermore, in a recent work of Pyo et al.^29^ the interplay between ligand orientations and p*K*_a_ of Au_25_ and Au_102_ NCs has been assessed by combining MD simulations and ED analysis.

To solve this issue, herein we propose an affordable computational procedure which includes conformational effects into a proper modeling of the ECD. This provides the possibility of a more realistic calculation of the ECD, not only for this Au_38_(SR)_24_ cluster, but also of a large variety of soluble chiral gold NCs. The method here adopted is an extension of a protocol recently published by some of these authors^30^ where the chiroptical properties of solvated peptides were investigated. The conformers produce an excellent qualitative description of the experimental chiral response^23^ of the 2-PET conformational transitions, thus demonstrating the validity of the proposed approach.

MD simulations of the solvated clockwise-Au_38_(2-PET)_24_ nanocluster were performed with the Gromacs package^31^ version 5.1.2. The OPLSA-AA topology was produced with the TPPMKTOP topology generator starting from the experimental X-ray geometry,^15,24^ while the Lennard-Jones parameters for Au and S atoms were taken from the GolP-OPLS-AA^32^ Force Field (FF), which contains the parameters to describe thiolate-protected gold nanoclusters compatibly with the OPLS-AA FF.^33^ The RESP charges and the parametrization procedure have been previously assessed for similar Au_25_ and Au_144_ nanoclusters.^34−36^ The OPLS-AA parameters of the toluene solvent were generated using LibParGen.^37^ All the MD simulations were performed in the NVT ensemble using the velocity rescaling algorithm^38^ to keep the temperature constant. All the bond lengths were constrained by using the LINCS algorithm.^39^ The Particle Mesh Ewald method^40^ was used with 34 wave vectors in each direction, a cubic interpolation of the fourth order and a 1.0 nm cutoff in order to compute the long-range electrostatic interactions.

The experimental X-ray Au_38_(2-PET)_24_ structure^15,24^ (see Figure S1 of the Supporting Information, SI) was inserted in a cubic box in the presence of toluene at a density resembling the reference experimental conditions,^23^ i.e., 303 K and 1.0 bar. For this purpose, since our simulations were carried out in the canonical ensemble (see below), the dimension of the solute–solvent box was then adjusted in order to achieve the average pressure of a box containing only toluene, with the same number of molecules (653), previously simulated in the NVT ensemble at the experimental temperature (303 K) and pure toluene density^41^ (857.55 kg/m^3^). After an initial slow thermal equilibration, we produced a trajectory of 150 ns constraining the gold atoms to preserve the experimental X-ray *staple* (i.e., Au – S) motifs. The importance and effect of the experimental metal architecture on the spectral features have been already discussed elsewhere.^24,42−44^ However, for the sake of completeness, we also performed an additional 50 ns all-atoms MD simulation to analyze the contribution of the gold atoms to the principal conformational transitions (essential for the modeling of the final spectrum) and evaluate the quality of Au-constrained MD. The results, discussed more in detail in the Supporting Information (Figures S2 and S3), show that the contribution of Au atoms to the most relevant transitions is almost null. Therefore, the significant conformations of the whole system can be extracted by analyzing only the conformational states of the flexible 2-PET ligands (see next sections).

The MD simulation described above was analyzed in terms of ED^45−47^ to sample the conformational landscape of the whole ensemble of the 2-PET ligands present on the nanocluster. The procedure adopted in this work has been previously explained in detail,^30^ and it is here only briefly summarized. The covariance matrix of all the 2-PET moieties atomic coordinates was constructed and diagonalized producing a set of eigenvectors with corresponding eigenvalues representing the mean square fluctuations. The eigenvectors showing the largest eigenvalues represent the directions along which the system undergoes the largest amplitude motions, i.e., the motions associated with the conformational transitions. Consequently, the projection of the Cartesian coordinates along the MD simulation onto such a subspace—i.e., the Principal Components Analysis—allows one to reduce the dimension and the complexity of the conformational space. Moreover, as also shown in the present case, it is possible to consider only the two eigenvectors associated with the two highest eigenvalues, hence allowing a straightforward conformational analysis on a bidimensional (2D) conformational landscape hereafter termed as essential space (ES). In this respect, each region of the ES showing a high density of projected points defines an *i*th conformational basin, and the number of projected points, divided by the total number of frames, allows one to evaluate the conformational probability *P*(*i*). Considering each *P*(*i*) and defining one of the above basins as the reference basin with a probability *P*_ref_ we can calculate the free energy differences between the *i*th basin and the reference one employing the standard relation:
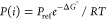
1where Δ*G*° corresponds to the standard Gibbs free energy difference between the *i*th and the reference basin on the ES within the following approximations: (i) negligible difference between the partial molar volumes of the Au_38_(2-PET)_24_ structures falling in the reference and *i*th basin (note that our simulations are carried out in the NVT ensemble); (ii) negligible differences between the quantum-vibrational molecular partition function of the Au_38_(2-PET)_24_ structures falling in the reference and *i*th basin.^48^ Therefore, we extracted from all the basins a certain number of Au_38_(2-PET)_24_ structures—hereafter termed as Representative Conformations (RC)—falling in the (0–2.5) kJ/mol free energy range. Because of the large dimensions of these basins, it was initially necessary to extract more than one RC from each basin. We selected, indeed, a set of 19 structures (see Figures S4 and S5 of the Supporting Information). However, for each basin, the extracted RC were further analyzed in terms of Root Mean Square Deviation (RMSD) to estimate their actual conformational difference. In this respect we assumed as spectroscopically equivalent RC with a RMSD < 1.78 Å following a preliminary analysis based on the outcome of the ECD spectra as reported in the Figure S5 of the Supporting Information. From this latter analysis we then found 10 of the previously extracted RC structures with RMSD values ≥2 Å, whereas the remaining RC were separated in two groups with the RMSD < 1.78 Å. This allowed us to reduce the number of the final RC from 19 to 12 to be considered for the quantum chemical calculations (see below). We calculated the ECD signal on each of the 12 RC, which was then weighted using the normalized probabilities, i.e., eq 1. The sum of all the weighted ECD signals produced the actual spectra reported in the following.

The geometries of the 12 Au_38_(2-PET)_24_ conformations extracted by the MD simulations were relaxed with specific constraints necessary to maintain the features of the corresponding basins, i.e., the values of the semiclassical coordinates. This was accomplished by relaxing only the ligands stretching and bonding angles and keeping frozen the corresponding proper dihedral angles as well as the metallic atoms coordinates. All the optimizations were realized at the DFT level,^49^ employing the standard GGA PBE exchange-correlation (xc) functional^50^ combined with the GRIMME-D3 dispersion terms.^51^ Furthermore, it was used a basis set of Slater-type orbitals (STO) of triple-ζ plus polarization quality (TZP), and the scalar relativistic effects were treated within the Zero Order Regular Approximation^52^ (ZORA).

The ECD spectra of the 12 conformations were calculated with the complex polarizability time-dependent DFT (polTDDFT) algorithm,^53^ where the rotatory strength *R* is defined as
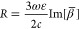
2

In eq 2, Im[β̅] represents the average over all the possible orientations of the imaginary part of the rotatory strength tensor, ω is the photon energy, ε corresponds to the imaginary part of the photon energy, and *c* is the speed of light. In this work, ε was taken as equal to 0.15 eV, allowing a direct comparison with a Lorentzian broadening with the same half width half-maximum. In addition, we used a cutoff of 3 eV above the excitation energy. All the 12 polTDDFT calculations were performed in the gas-phase as a compromise between computational cost and accuracy, moreover considering that solvent effects on the ECD are generally very pronounced for polar molecules in polar solvents.^54−56^ Therefore, since the present cluster is a nonpolar molecule as well as the solvent (toluene), we did not consider the solvent effect in the calculation. The LB94^57^ xc functional, which includes the correct asymptotic behavior, was employed together with the TZP basis set and the ZORA scheme for the relativistic effects. Each spectrum was multiplied by the corresponding statistical weight (eq 1) and summed up to obtain the final averaged spectrum, then compared with the experimental reference^23^ to evaluate the quality of our result.

An additional analysis has been performed calculating the polTDDFT ECD spectrum of the experimental X-ray structure^15,24^ with the LB94, and the hybrid B3LYP^58^ xc functional which contains a portion of the Hartree–Fock nonlocal exchange. The latter one provides a more accurate description, in particular of the metallic spectral features. Additionally, for the B3LYP calculation we employed the Hybrid Diagonal Approximation^59^ (HDA) which allows us to perform polTDDFT calculations with hybrid functionals, although it is still computationally demanding with respect to standard GGA functionals like, for instance, LB94. For this reason, we restricted the HDA-B3LYP calculation to the experimental X-ray diffraction structure only. All the other parameters of the calculation (e.g., basis set, ZORA) were maintained also in these two new calculations.

We report in Figure 2 the spectrum of the covariance matrix eigenvalues (mean square fluctuations) of the 2-PET (-SC_2_H_4_Ph) which may help us in predicting the number of modes needed to describe the collective motions of the Au_38_(2-PET)_24_ nanocluster, i.e., the size of the ES.

**Figure 2:**
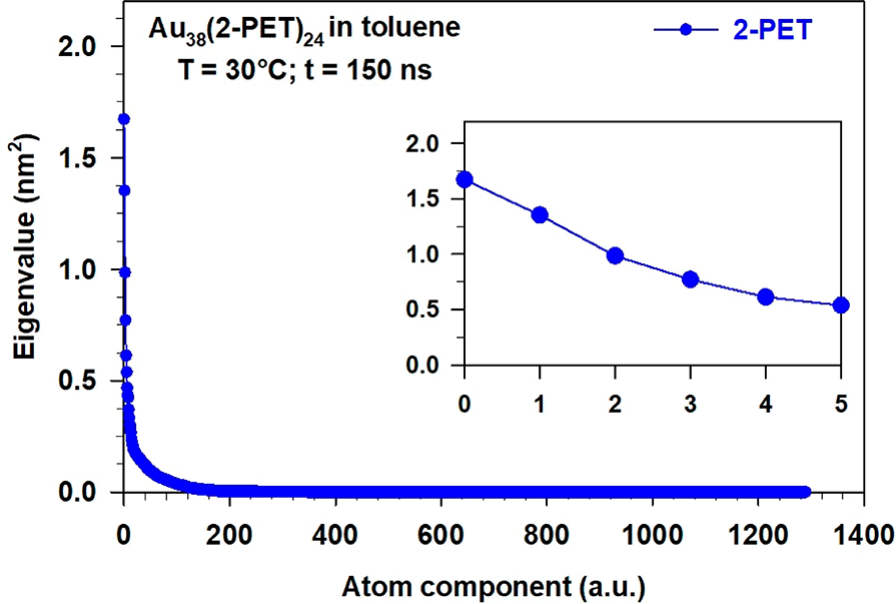
Plot of the eigenvalues resulting from the diagonalization of the 2-PET (-SC_2_H_4_Ph) ligands covariance matrix. The matrix was built with the atomic positions of the thiolate ligands extracted from the MD trajectory of 150 ns at *T* = 30 °C. The first 6 eigenvalues are highlighted in the right inset of the figure.

The spectrum of the eigenvalues rapidly decays to 0, with only the first 6 eigenvalues (inset of Figure 2) larger than 0.5 nm^2^. However, since the first pair of eigenvalues accounts for the largest fraction of the whole matrix trace (i.e., the whole fluctuation), we can simplify the analysis considering only the corresponding first 2 eigenvectors. In addition, it should be remarked that the inclusion of, at least, the third eigendirection, although possible in principle,^60^ might be very expensive in the case of highly complex systems like the present one. Therefore, well aware of the possible incompleteness of the conformational analysis, we decided to adopt a 2D ES for representing the best compromise between accuracy and computational effort. The comparison with experimental data will provide a validation of this approximation.

The conformational landscape, obtained from the procedures described previously, is depicted in Figure 3.

**Figure 3:**
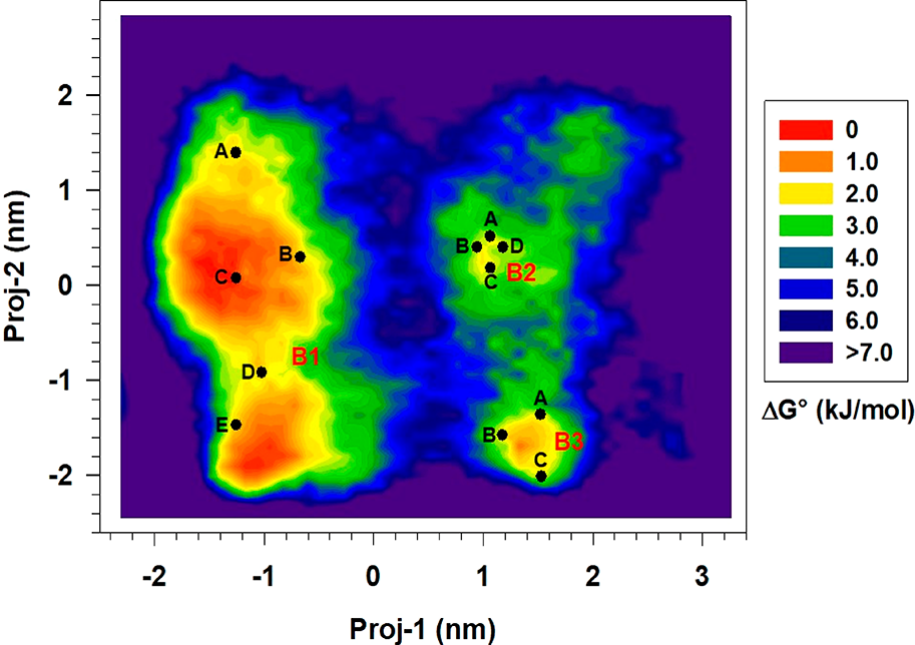
Probability pattern built with the MD trajectory projections (proj-1, and proj-2 in nm) on the first two essential modes. Each square, graphically smoothed in the histogram, represents a conformational basin associated with a specific relative free energy Δ*G*° value reported in kJ/mol (right side of the figure). The three low-energy basins are labeled as B1, B2, and B3, respectively, while the positions of the selected 12 low-energy conformations are marked with black dots.

In Figure 3 one observes three low-energy conformational basins (B1, B2, and B3) surrounded by barriers with Δ*G*° ≥ 3 kJ/mol. The three basins differ in terms of dimensions and energy range, indeed while B2 and B3 are quite small and associated with higher Δ*G*° values (>2.0 kJ/mol), B1 is very extended and contains the most probable conformers. Such a result shows the possibility of having different, more or less probable, ligand conformations in different regions of the ES. Therefore, the reduction from 19 to 12 structures thanks to the RMSD analysis does not interfere on the absence of low energy structures in the B2 and B3 basins. As also previously reported, the extension of the three basins, in particular B1, suggests the presence—in each basin—of different conformations which can rapidly mutually interconvert. On the other hand, the B1 ⇆ B2, B1 ⇆ B3, and B2 ⇆ B3 conformational transitions appear as more hindered being characterized by higher energy barriers. This particular feature prompted us to extract a number of RC (12) much larger than the actual number of basins (3) as already described. The energy features of the 12 RC, with their own normalized probabilities, are reported in Table 1. Additional information (i.e., the Cartesian coordinates) can be found in Table S1 of the Supporting Information.

**Table 1:** Relative Free-Energies and Normalized Probability Values of the 12 Au_38_(SC_2_H_4_Ph)_24_ Representative Conformations Selected within the ED Analysis

Basin-Representative Conformation	Δ*G***°** (kJ/mol)	*P*(*i*)_norm_^a^
B1-A	2.0	0.079
B1-B	1.9	0.082
B1-C	0.2	0.165
B1-D	1.8	0.086
B1-E	1.7	0.090
B2-A	2.3	0.071
B2–B	2.4	0.068
B2-C	2.3	0.070
B2-D	2.3	0.070
B3-A	2.3	0.070
B3-B	2.0	0.079
B3-C	2.3	0.071

a ∑_*i* = 1_^*RC*^*P*(*i*)_norm_ = 1

*Electronic Circular Dichroism Spectra*. The statistically averaged calculated ECD spectrum, using all the selected 12 RC, is compared with the experimental one^23^ in Figure 4.

**Figure 4:**
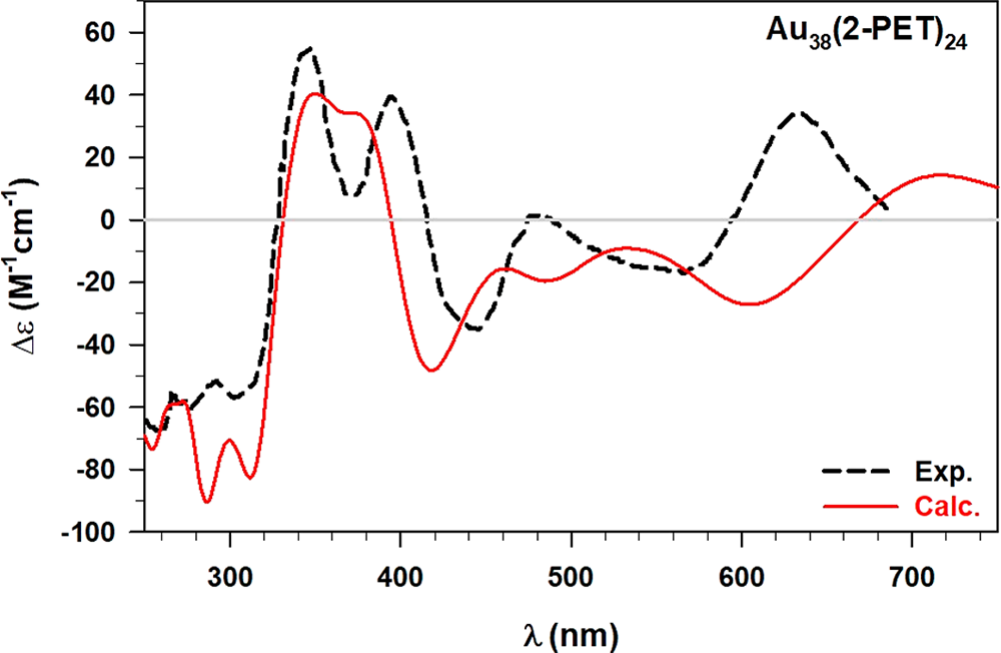
Comparison between the experimental (Exp., black dashed line) and calculated (Calc., red line) ECD spectrum averaged over 12 conformations of the Au_38_(2-PET)_24_ cluster. All the details of the calculation (i.e., xc functional, basis-set, HWHM) have been reported in the text.

Figure 4 reveals a very good agreement with the experimental pattern in the regions between 250 and 450 nm, while some energy shifts of the calculated ECD emerge when moving toward higher wavelength values. Indeed, the two calculated peaks at 539 nm (exp. peak at 473 nm) and 712 nm (exp. peak at 627 nm) are systematically found to be red-shifted and also a small maximum peak appears around 460 nm. However, this systematic drawback can be explained by recalling that the metallic response, predominant in this low-energy region, can suffer from the well-known^24,42^ attractive character of the LB94 xc functional. On the contrary, the high energy range, where an excellent qualitative agreement between experimental and calculated result is observed, is mostly determined by the spectral features of the flexible thiolate ligands. Therefore, Figure 4 reveals that the combination of a dynamics approach with polTDDFT calculations brings to a qualitatively correct description of the optical response of high-flexible systems, such as the ligands of protected NCs. This result has been corroborated by comparing the statistical ECD with those calculated with different xc functionals but only considering the experimental X-ray structure.^15,24^ All the results are reported in Figure 5.

**Figure 5:**
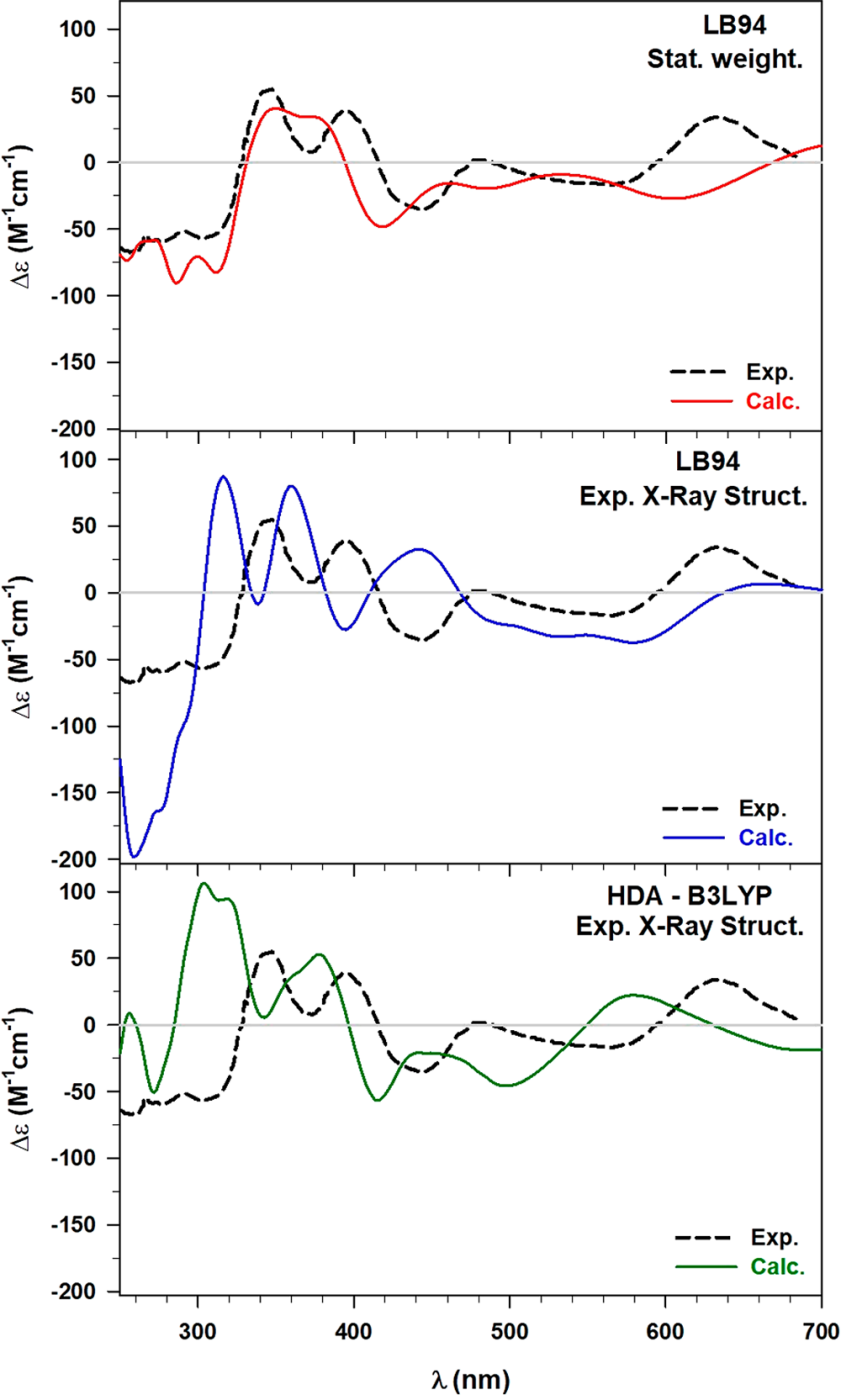
Comparison between the experimental (Exp., black dashed line) and the calculated ECD spectrum. (Top) statistically weighting 12 conformations and by using the LB94 xc functional (upper panel, red line), (middle) using the exp. X-ray structure and the LB94 xc functional (middle panel, blue line), (bottom) using the exp. X-ray structure and the HDA in combination with the B3LYP xc functional (lower panel, green line).

Concerning the high energy range of the ECD spectrum, it can be observed that the calculation on a single structure, even though it is the experimental one obtained by X-ray diffraction, does not reproduce properly the experimental spectral pattern of the 2-PET. Indeed, using the LB94 in the polTDDFT calculation (i.e., middle panel of Figure 5), a negative intense artifact arises around 250 nm, and the two following positive maximum values are blueshifted with respect to experimental peaks. The same blueshift is observed in correspondence of the experimental peak located at 473 nm and even a not negligible difference in intensity arises for this feature. On the contrary, some qualitative similarities between the two ECD spectra can be observed at higher wavelengths, i.e., beyond 600 nm.

In the lower panel of Figure 5, we compare the experimental ECD with the result obtained using the hybrid functional B3LYP (polTDDFT in combination with the HDA). It has been already shown^59^ that this approach increases the accuracy of the description of the Au optical response, even though it requires a significant computational effort. Indeed, the calculated spectrum beyond 450 nm is characterized by two maxima (i.e., 452 nm and 585 nm) in reasonable agreement with the experimental values at 473 and 627 nm, respectively. Therefore, despite a minor difference in terms of energy distributions, the hybrid calculation is able to reproduce much better the experimental features in the region of the metallic response. In contrast, in the ligand region of the spectrum, we can notice discrepancies which confirm again the importance of including the ligand conformational effects to model the ECD. The present B3LYP-HDA calculation has been rather demanding, 6 days using 144 cores on the Galileo supercomputer of CINECA (Bologna, Italy) consisting of several nodes each with 2 x CPU Intel CascadeLake 8260, with 24 cores each. Instead, for each structure calculated at the LB94 level, the SCF required 5 h at 144 cores on the Galileo supercomputer, while the polTDDFT section took 15 h with 24 CPU on a HP ProLiant DL580 Gen10 server. The latter consists of 4 processors each with 18 cores Intel Xeon Gold 6140 CPU @ 2.30 GHz, in total 72 cores and 728 GB of RAM. We are working on a new implementation of the B3LYP-HDA scheme based on the Resolution of the Identity instead of the presently implemented numerical integration, which is very promising in terms of numerical efficiency.

In conclusion, the above data clearly suggest that for a correct ECD modeling of chiral RS-AuNCs, at least the relevant conformations of the protective ligands, must be included in the response predictions. This effect becomes more and more relevant with the increase of the ligand flexibility, but also in the presence of intramolecular interactions as well as solvent effects, especially considering the aqueous environment. The metal core description, which is much less affected by conformational effects, suffers the limitations of the LB94 functional. However, preliminary calculations using the HDA^59^-B3LYP scheme demonstrate the possibility of a better description also of the Au response.

In conclusion, we propose a computational approach that is able to efficiently and accurately include the conformational effects of the dynamic protective ligands into the ECD modeling of chiral RS-AuNCs. Extending a protocol recently proposed for solvated peptides, we investigate these more complex gold NCs and their chiroptical properties focusing on the well-known Au_38_(2-PET)_24_ cluster. We start with a MD simulation of this system in toluene using an accurate force-field, which is then analyzed to sample the conformational space of the ligands via the ED analysis. This statistical tool allows us to consider only the *essential* conformational dynamics, hence to extract properly the most relevant conformations of the system. A reduced number of structures is then considered for the first-principles polTDDFT calculations of the ECD. We obtain an excellent agreement between the experimental and calculated spectral features of the 2-PET ligands both in terms of energy and intensity. Such a result is corroborated by calculating the ECD only on the X-ray crystal structure with two different xc functionals (LB94, B3LYP) and finding a much worse agreement with the experimental response. This demonstrates the need to include conformational effects into the modeling of the ECD of gold nanoclusters, together with the proposal of a reproducible and affordable computational approach to accomplish this goal. Some limitations are found in the description of the metal core response, but we assume that they are related to the chosen xc-functional and not to the procedure here proposed.

## Supporting Information Available

The experimental X-ray structure, the comparison between all-atoms and Au-constrained MD simulation, the additional analysis on the conformational landscape for the extraction of the final set of 12 RC, and the atomic coordinates of the conformers (PDF)
Transparent Peer Review report available (PDF)
