# Supplementary material for: The Conformational Dynamics of the Ligands Determines the Electronic Circular Dichroism of the Chiral Au38(SC2H4Ph)24 Cluster
Source: J Phys Chem Lett. 2023 Feb 14;14(7):1941–8. doi: 10.1021/acs.jpclett.2c03923 (PMC9940292; doi:10.1021/acs.jpclett.2c03923)
Supplement: Supplementary file 1 — jz2c03923_si_001.pdf [file jz2c03923_si_001.pdf]

Supporting Information for:

The Conformational Dynamics of the Ligands Determines the Electronic Circular Dichroism of the Chiral $Au_{38}(SC_2H_4Ph)_{24}$ Cluster

M. Monti[1], G. Brancolini[2], E. Coccia[1], D. Toffoli[1], A. Fortunelli[3*], S. Corni[2,4], M. Aschi[5*] and M. Stener[1*]

[1] Dipartimento di Scienze Chimiche e Farmaceutiche, Università di Trieste, Via L. Giorgieri 1, 34127 Trieste, Italy.

[2] Centro S3, CNR Instituto di Nanoscienza, via Campi 213/A, 41125 Modena, Italy.

[3] CNR-ICCOM, Consiglio Nazionale delle Ricerche, via G. Moruzzi 1, 56124, Pisa, Italy.

[4] Dipartimento di Scienze Chimiche, Università di Padova, Via Francesco Marzolo 1, 35131 Padova, Italy.

[5] Dipartimento di Scienze Fisiche e Chimiche, Università dell'Aquila, Via Vetoio, 67100, l'Aquila, Italy

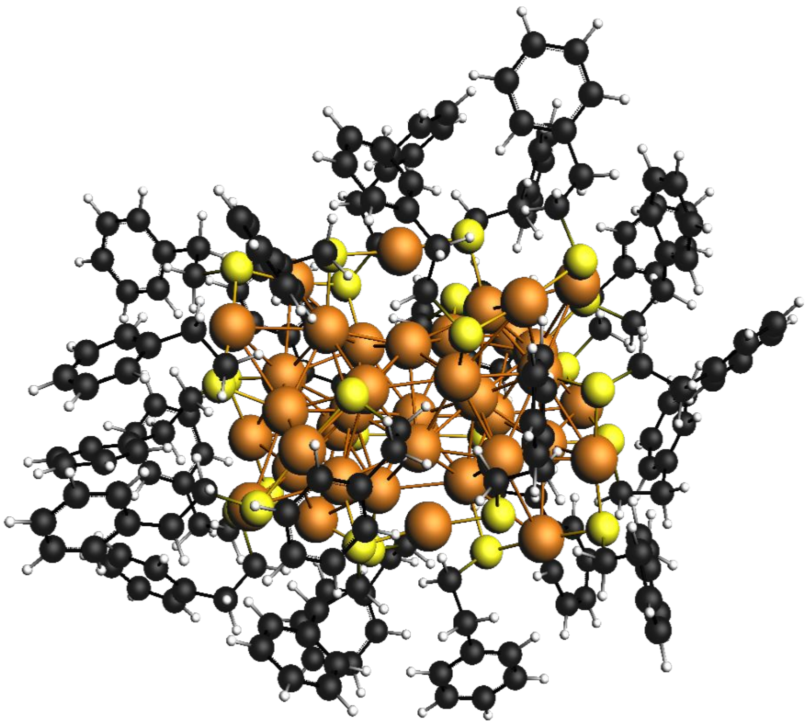

Figure S1. Ball and stick model of the experimental X-ray $Au_{38}(SC_2H_4Ph)_{24}$ structure[1]. Au, S, C, and H atoms are reported in orange, yellow, black, and white, respectively.

All-atoms MD vs Au-constrained MD

It is well known the importance of calculating the ECD spectra on realistic geometries to obtain reliable results and in the specific case of gold nanoclusters, the relevance of a reasonable metal-sulphur architecture has proven to be fundamental for the ECD calculation[1,2]. Therefore, the resolution of several X-ray gold nanostructures has represented a breakthrough for a suitable analysis of the spectral features. Naturally, the X-ray structure is less important when the chirality regards the ligands because of their higher conformational flexibility, which characterizes their ECD in the liquid phase. Hence, the calculation of the ECD on a gas phase nanocluster and its comparison with respect the experimental liquid phase ECD must consider the flexibility of the ligands as well as the necessity of a reliable geometry of the staples (Au-S structures). Starting from these considerations, we constrained the gold atoms to maintain the experimental geometry for the metallic region but without affecting the ligands internal motions. However, we wanted to verify the validity of our choice estimating the effective contribution of the gold atoms to the conformational transitions and to understand if their exclusion in the conformational analysis is justified. Such an estimate was

performed running a 50 ns all-atoms MD simulation with no constraints and calculating the first two total eigenvectors components of the covariance matrix for Au and S (see Figure S2).

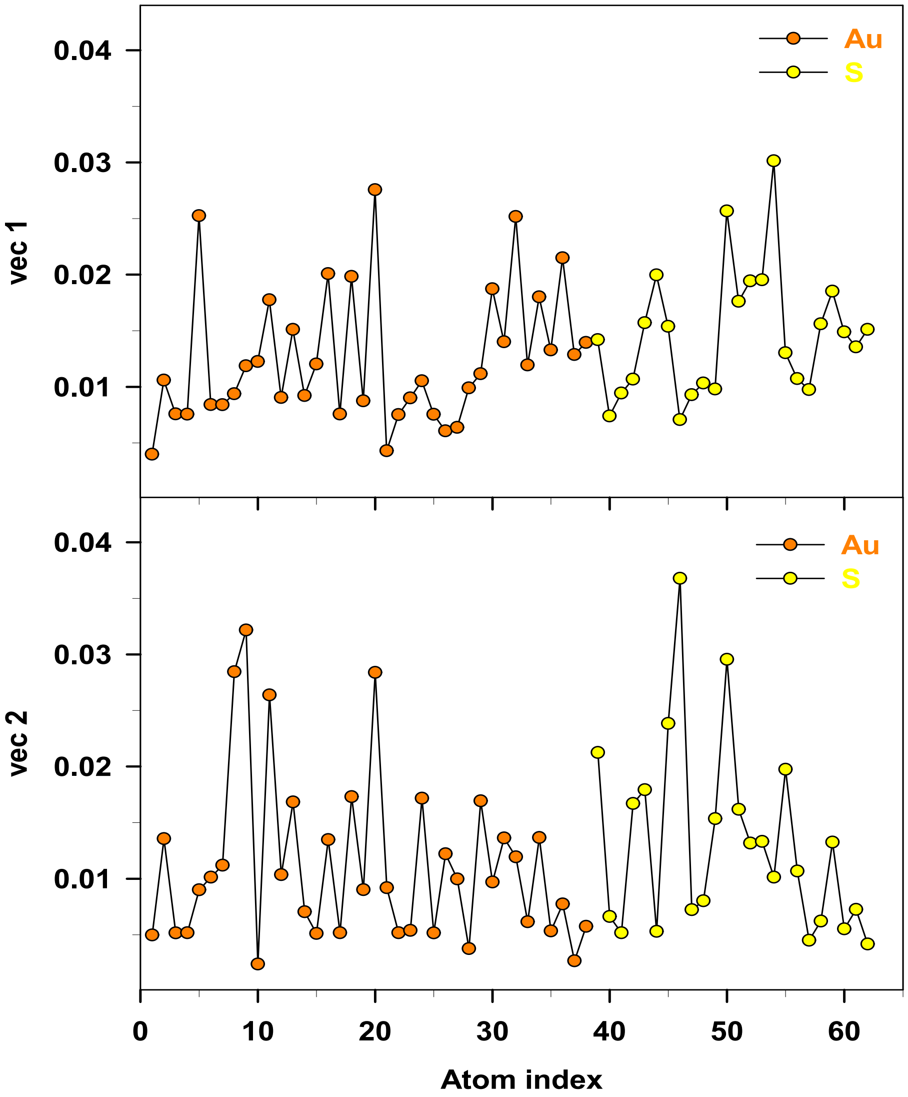

Figure S2. First eigenvector (upper panel) and second eigenvector (lower panel) total components for the gold (orange) and sulphur (yellow) atoms calculated from the 50 ns all atoms free-MD simulation.

Looking at the value scale of the total first pair of eigenvectors, it is worth noting that the contribution of both gold and sulphur atoms to the most relevant conformational transitions is quite small. Such result justifies the constraints on the gold atoms and thus their exclusion from the conformational analysis we carried out to find the most probable structures. The following Figure S3 underlines how small is the contributions of the staple atoms with respect to the ligand ones, which start from atom index 63.

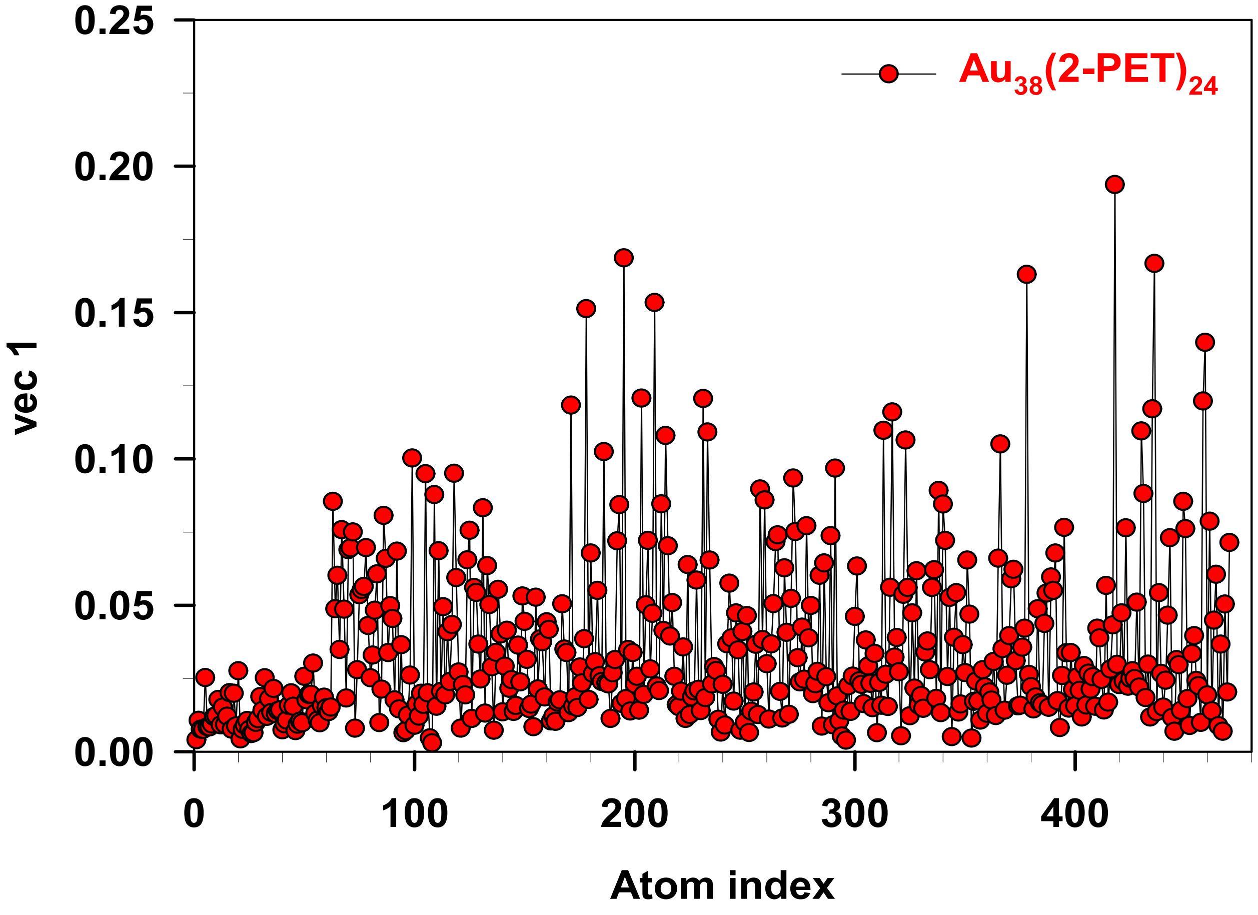

Figure S3. First eigenvector total component for the $Au_{38}(2\text{-}PET)_{24}$ atoms calculated from the 50 ns all atoms free-MD simulation.

As we can see, we obtained that the total component of the first eigenvector, which is directly related to the atomic contribution to the most relevant internal transitions, is on average 10 times larger for the (2-PET) atoms with respect to the values found for the Au/S atoms. This result is easily understood comparing the flexibility, and thus the fluctuations, of the metallic and organic components of the system.

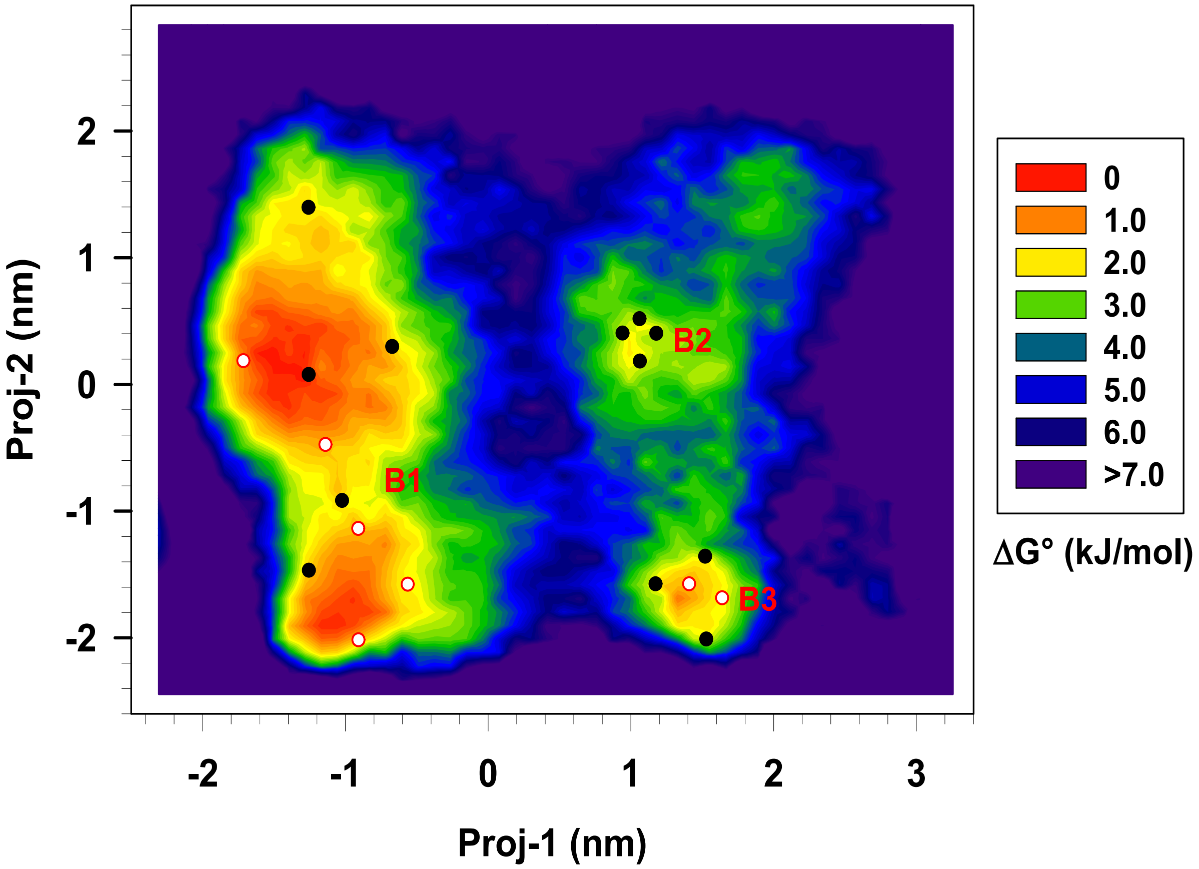

Figure S4. Probability pattern built to represent the ΔG° landscape on the conformational space. The positions of the 19 conformations initially extracted have been marked with dots (i.e., black for the conformations of the final set, white for the conformations discarded in the final analysis).

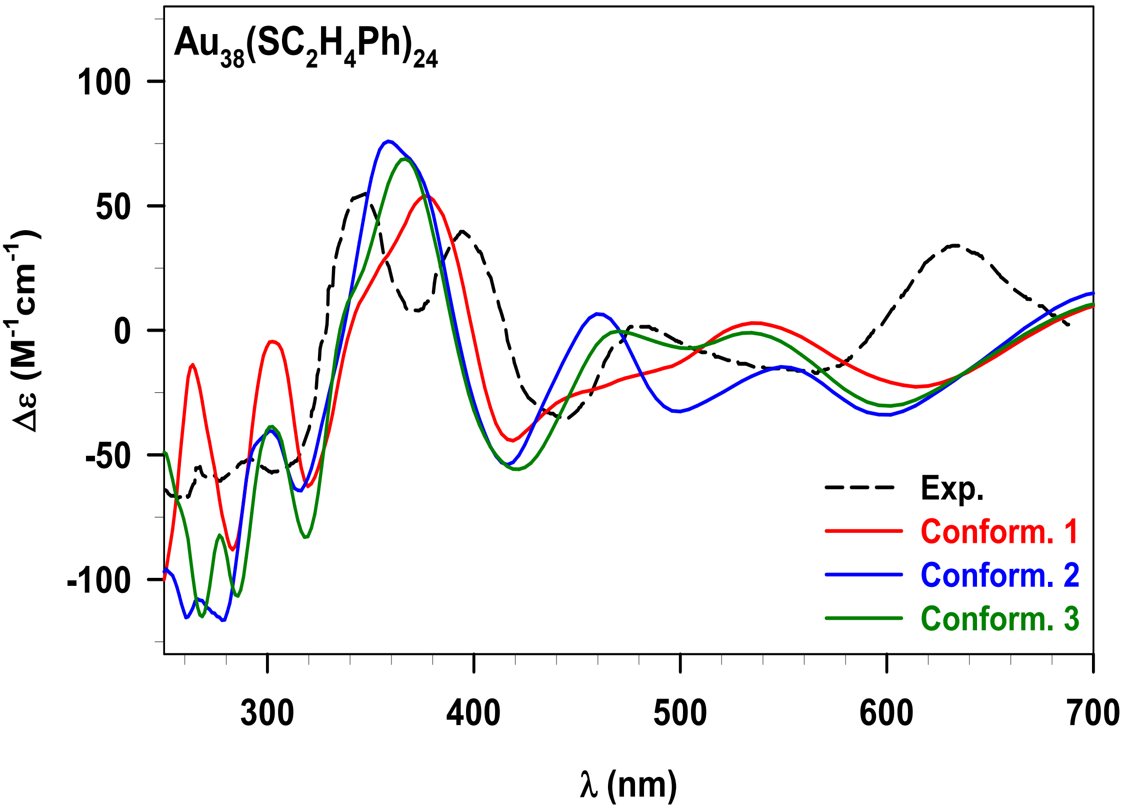

Figure S5. Experimental (Exp., black dashed line) and Calculated ECD spectra of three similar conformations in terms of RMSD values (red, blue, and green solid line). The computational details of the polTDDFT calculations have been reported in the Computational Section.

Table S1. Atomic Coordinates of the 12 selected $Au_{38}(2\text{-}PET)_{24}$ conformations

**B1-A**	x (Å)	y (Å)	z (Å)
Au	0.098494	-0.071185	0.052891
Au	2.903032	-0.054136	0.10086
Au	1.409987	2.399101	0.019794
Au	1.640136	-1.970445	-1.469053
Au	1.507063	0.263255	2.507801
Au	0.375789	4.77246	1.04364
Au	3.262129	4.467004	0.812334
Au	3.983451	2.250211	-1.128596
Au	-0.691273	2.067122	1.904326
Au	1.607102	0.828972	-2.274374
Au	1.821398	3.028805	2.729325
Au	5.739221	0.001018	0.06139

Au	4.322615	-2.402107	0.018144
Au	4.316982	1.785135	1.689938
Au	1.700332	-2.253163	1.36238
Au	4.166636	-0.535662	-2.332675
Au	4.104844	-0.922612	2.466586
S	6.084377	-1.214399	-3.645056
Au	2.132918	5.754466	3.202088
Au	-0.439934	4.316086	3.689383
Au	4.342627	3.947036	3.577175
Au	0.274268	1.608253	4.615525
Au	3.317134	1.339427	4.37881
Au	2.00762	3.664348	5.425171
S	5.072231	5.934121	-0.00433
S	-0.527771	5.789977	-0.933102
S	5.905406	-1.813162	3.800121
S	-2.138696	-0.887273	0.314693
S	7.906572	1.022775	0.248992
S	6.770719	4.155673	3.545776
S	0.380234	-3.608151	2.870861
S	0.134906	2.035255	-3.697546
S	-1.071683	-0.143769	5.58199
S	3.01467	4.730818	7.387963
S	4.439875	-4.824955	0.34738
Au	-1.021584	-3.095294	-0.313407
C	-2.510567	-1.219685	2.057432
Au	2.148411	-4.77749	-0.239897
C	5.03297	-5.311173	-1.347855
Au	4.803638	6.811594	2.295339
C	6.669195	5.048317	-0.093694
Au	7.206851	2.508967	1.911277
C	8.362756	2.062959	-1.22919
C	7.779455	3.518016	4.962473
Au	-0.133427	3.870667	-2.278788

C	0.6105	7.092465	-1.607025
C	-1.351834	1.168608	-4.340551
Au	-0.216908	-1.7521	4.102174
C	-2.889766	-0.01053	5.27036
C	1.351906	-4.770908	3.938667
Au	3.96159	6.168321	5.727063
C	4.426285	3.538103	7.381467
S	4.793945	7.925932	4.349206
C	6.550741	8.023337	4.927079
S	-0.060968	-5.142005	-0.910326
C	-0.694973	-6.35024	0.341831
S	-2.895396	2.346741	0.973011
S	1.527827	8.072952	3.117833
Au	-2.707728	4.704701	1.630149
C	-2.670168	2.312162	-0.841584
Au	-0.566171	7.348636	2.555394
C	1.263513	8.647932	4.868277
S	-2.79307	6.975622	2.047728
C	-3.423105	7.659453	0.456888
S	0.90785	-2.964547	-3.573696
S	4.221691	3.955649	-2.743562
Au	2.10749	-1.133576	-4.581917
C	-0.876999	-2.496131	-3.6652
Au	3.717947	2.052719	-4.347189
C	2.889685	5.16122	-2.732053
S	3.168551	0.461356	-5.914359
C	4.5569	-0.167801	-6.938706
S	-2.290822	4.758613	5.266902
S	3.710653	-0.173778	6.330097
Au	-0.811877	3.481391	6.751334
C	-3.558882	3.615303	4.581155
Au	1.925916	1.246214	7.331806
C	3.070289	-1.832439	5.868743

S	0.329321	2.440311	8.538129
C	-0.823849	1.073503	9.041691
Au	6.899065	-2.222017	-1.804406
C	5.656649	-2.72304	-4.639175
S	8.032857	-3.504063	-0.15484
C	7.700613	5.932615	-0.798582
C	8.02778	7.149334	0.033055
C	8.746859	7.006621	1.228944
C	7.577803	8.423273	-0.330848
C	8.934951	8.09466	2.080902
C	8.37944	9.337878	1.762012
C	7.764291	9.515272	0.520632
C	-3.819103	9.127125	0.609999
C	-3.557867	10.01345	-0.596901
C	-3.688929	11.409267	-0.444689
C	-2.915832	9.588349	-1.768672
C	-3.373345	12.219227	-1.538217
C	-3.563015	11.687661	-2.82134
C	-3.212374	10.326158	-2.938622
C	9.792891	-2.985083	-0.400008
C	9.959937	-1.494257	-0.686072
C	11.301371	-1.059477	-1.207402
C	12.070221	-1.838807	-2.095412
C	12.043157	-0.048457	-0.566042
C	12.885548	-1.12091	-2.985731
C	13.464752	0.076374	-2.530039
C	13.058998	0.59708	-1.292872
C	-3.995517	2.180164	-1.580486
C	-4.70739	3.513637	-1.731657
C	-6.070555	3.658544	-1.46645
C	-3.971167	4.672563	-2.027377
C	-4.620052	5.828689	-2.445702
C	-6.023179	5.826273	-2.486195

C	-6.65101	4.930619	-1.562407
C	7.371449	2.039635	-2.397866
C	8.005029	2.006633	-3.767753
C	9.023834	1.095639	-4.08433
C	7.716161	3.017082	-4.69714
C	9.480286	0.991537	-5.400468
C	8.049126	2.816648	-6.043398
C	8.864767	1.739437	-6.411043
C	1.694494	7.535171	-0.617519
C	2.064655	8.990056	-0.72117
C	1.122938	10.008411	-0.48485
C	3.354857	9.396877	-1.085317
C	1.575323	11.265927	-0.094362
C	2.78209	11.6955	-0.686951
C	3.614516	10.755003	-1.314497
C	7.041782	6.869587	5.800744
C	7.716397	7.214632	7.110074
C	7.667592	8.479812	7.709044
C	8.339944	6.187257	7.831458
C	8.585597	8.82299	8.703452
C	9.535883	7.879659	9.099572
C	9.15687	6.5266	8.919366
C	-2.189137	2.134157	-5.181211
C	-1.974155	2.056248	-6.674914
C	-1.045364	1.217867	-7.309469
C	-2.872674	2.720365	-7.537709
C	-0.917584	1.310161	-8.708057
C	-1.848791	1.962578	-9.515226
C	-2.601803	2.989673	-8.872182
C	-1.45296	-2.81526	-5.049713
C	-1.961706	-4.235071	-5.133463
C	-1.528265	-5.108493	-6.138548
C	-2.997212	-4.679214	-4.295347

C	-1.983674	-6.432597	-6.163288
C	-3.202133	-6.044666	-4.105722
C	-2.758654	-6.922345	-5.106606
C	2.86187	-4.724917	3.740863
C	3.596374	-5.913019	4.318679
C	4.674242	-6.482621	3.62569
C	3.174668	-6.542773	5.501688
C	5.434172	-7.497124	4.214465
C	5.045343	-8.063309	5.430997
C	3.966709	-7.506787	6.127234
C	0.722849	7.535939	5.758326
C	0.813908	7.69954	7.250134
C	-0.021169	6.922118	8.073253
C	1.676523	8.602423	7.895743
C	0.347836	6.719067	9.405147
C	1.101004	7.688849	10.071544
C	1.84282	8.579853	9.286021
C	6.727103	-0.241115	4.299951
C	3.22941	6.291938	-3.705837
C	2.802581	5.884429	-5.11258
C	1.581789	5.211024	-5.290316
C	3.59186	6.132006	-6.241264
C	1.171028	4.769827	-6.545638
C	3.273714	5.53537	-7.465253
C	2.038375	4.903299	-7.635185
C	7.919545	-0.615644	5.218622
C	9.192593	-0.908976	4.467136
C	10.045584	-1.958816	4.834808
C	9.650924	-0.031265	3.468552
C	11.120147	-2.298349	4.006621
C	11.707229	-1.290129	3.234041
C	10.951796	-0.138472	2.974249
C	6.639299	-2.937193	-5.815249

C	7.376092	-4.232441	-5.583632
C	6.811809	-5.460914	-5.955118
C	8.634126	-4.236798	-4.963112
C	7.424652	-6.653817	-5.564769
C	8.422946	-6.631443	-4.587918
C	9.118316	-5.426929	-4.404036
C	-4.921934	3.677953	5.306965
C	-5.97561	4.086473	4.306941
C	-6.463072	5.398585	4.255866
C	-6.482033	3.162541	3.380471
C	-7.089987	5.868033	3.099488
C	-7.623861	4.92975	2.206421
C	-7.439457	3.5606	2.4454
C	-3.627771	-1.273755	5.704689
C	-3.951479	-1.455473	7.166048
C	-4.306085	-2.729473	7.644919
C	-3.751945	-0.472928	8.145792
C	-4.983375	-2.81869	8.868519
C	-5.023106	-1.730077	9.748889
C	-4.478281	-0.50734	9.34004
C	9.220889	3.989697	4.944377
C	10.046842	3.421693	6.083102
C	9.501355	2.620064	7.09867
C	11.313319	3.975152	6.356336
C	10.377788	1.936641	7.956466
C	12.19692	3.339918	7.228846
C	11.702181	2.354819	8.09715
C	5.187623	1.001709	-7.685528
C	4.44105	1.526595	-8.89687
C	4.888344	2.713957	-9.493256
C	3.470147	0.784814	-9.578901
C	2.636771	1.405297	-10.51253
C	4.135051	3.27921	-10.528769

C	2.875631	2.73942	-10.850921
C	3.049684	-2.772049	7.072287
C	4.393811	-3.110734	7.680407
C	4.505184	-3.271421	9.063473
C	5.405709	-3.668238	6.886845
C	6.721284	-3.653142	7.334721
C	5.788293	-3.598218	9.547452
C	6.904037	-3.344014	8.694895
C	5.577267	3.834691	8.331863
C	5.960381	2.644281	9.20179
C	5.223229	1.447124	9.269344
C	7.147126	2.704722	9.960043
C	5.833661	0.314556	9.817875
C	6.796409	0.451902	10.81398
C	7.567667	1.628518	10.749928
C	-0.115818	-0.223522	9.445881
C	-0.449451	-0.836326	10.792586
C	0.059658	-2.12288	11.054561
C	-1.482248	-0.394944	11.621851
C	-0.030867	-2.626356	12.351139
C	-1.358333	-0.777431	12.981247
C	-0.428059	-1.760842	13.377053
C	-0.702845	-7.761293	-0.256109
C	-0.002344	-8.832168	0.560063
C	0.478807	-8.636454	1.860978
C	0.110024	-10.126497	0.020965
C	1.172785	-9.667036	2.506488
C	1.064475	-11.004206	0.553201
C	1.741584	-10.686334	1.747334
C	-3.966506	-1.640313	2.24158
C	-4.164218	-3.138476	2.237357
C	-5.35086	-3.734452	1.784109
C	-3.204028	-3.991892	2.819078

C	-5.609524	-5.059965	2.151102
C	-4.537409	-5.950749	2.2284
C	-3.335063	-5.383615	2.695409
C	4.761062	-6.790669	-1.643362
C	3.613855	-7.026979	-2.605784
C	2.829462	-8.19603	-2.52245
C	3.196178	-6.102569	-3.58351
C	1.592122	-8.189202	-3.17247
C	1.579313	-7.686783	-4.480657
C	2.396414	-6.548912	-4.654214
H	-4.051657	6.667199	-2.852923
H	14.182042	0.617042	-3.150138
H	-6.624362	2.830497	-1.01718
H	13.271696	-1.601986	-3.887429
H	0.095182	9.718489	-0.23697
H	-3.371503	13.30519	-1.403445
H	11.630215	0.427535	0.325254
H	8.493006	10.182585	2.443875
H	13.561661	1.471182	-0.873915
H	-3.897324	11.84144	0.535682
H	-3.666297	12.322881	-3.701265
H	-4.4658	0.350259	10.014981
H	11.680733	-2.807531	-2.428547
H	5.209019	-3.855442	5.829459
H	7.907638	-3.306703	9.125076
H	12.367103	1.850611	8.800367
H	4.286266	1.331375	8.70479
H	1.773775	0.873387	-10.916265
H	0.595304	-2.670716	10.276386
H	9.481621	7.962785	3.0174
H	-2.149684	0.420432	11.347148
H	8.651249	9.858755	9.042666
H	7.557674	-3.82284	6.656504

H	7.133623	-0.408046	11.395145
H	5.322796	-0.646173	9.702738
H	9.132812	6.023954	1.511511
H	7.00934	9.255213	7.315028
H	0.898448	11.985471	0.369034
H	0.430144	-3.588214	12.589626
H	-1.955695	-0.262017	13.736829
H	-0.319982	-2.031375	14.427994
H	10.323632	8.139894	9.808858
H	-2.879263	4.621026	-1.971403
H	-6.558929	6.714381	-2.829986
H	6.976882	-7.604526	-5.863119
H	-7.704209	5.067472	-1.305229
H	7.044604	8.553001	-1.275217
H	8.795977	-7.558499	-4.149789
H	9.712594	5.735447	9.429344
H	7.371329	10.49439	0.240947
H	-3.059248	9.87481	-3.920784
H	-2.584625	8.554635	-1.881973
H	5.90548	-5.484071	-6.56404
H	8.261805	5.146143	7.521792
H	9.715942	-2.659455	5.607201
H	3.07768	12.745356	-0.636761
H	9.407425	0.405795	-3.328436
H	11.661329	-3.23134	4.182023
H	-0.283252	0.668244	-6.753434
H	7.046019	3.83231	-4.413907
H	-6.124614	6.11307	5.011594
H	4.109384	8.641922	-1.321374
H	10.020303	-5.406755	-3.788447
H	-7.28647	6.933328	2.969602
H	4.983271	-6.054797	2.670517
H	9.150709	-3.29243	-4.776875

H	4.556599	11.069404	-1.767718
H	-0.175713	0.663995	-9.186017
H	-0.739209	6.238573	7.607989
H	12.669749	-1.445348	2.743746
H	1.028205	4.974304	-4.37461
H	10.237854	0.245472	-5.650078
H	-0.808034	-4.762073	-6.883495
H	6.293022	-7.903422	3.675905
H	7.682554	3.511772	-6.803089
H	-4.302312	-3.585423	6.96398
H	-8.225284	5.260276	1.357878
H	9.133393	1.58433	-7.458439
H	-3.432536	-3.976086	-3.578131
H	4.541243	6.662991	-6.128006
H	2.307726	9.269168	7.304744
H	5.775183	3.22409	-9.111594
H	2.303182	-6.156473	6.036173
H	9.030404	0.816284	3.152862
H	-3.628538	3.346574	-7.044277
H	-6.187151	2.112098	3.446018
H	-1.730509	1.949735	-10.601533
H	3.650856	-3.147001	9.730584
H	11.352813	0.651565	2.338453
H	-0.177647	5.954492	9.983954
H	-7.899037	2.823544	1.784393
H	8.458559	2.299483	7.065413
H	-1.667754	-7.102482	-6.965613
H	0.221619	4.243316	-6.6611
H	4.437433	4.237773	-10.95621
H	5.640914	-8.859032	5.882136
H	-5.378909	-3.784346	9.191184
H	-3.333188	0.488888	7.843236
H	11.694578	4.760046	5.695506

H	7.769063	3.602127	9.906687
H	-3.243077	3.665109	-9.442517
H	-3.85415	-6.400477	-3.306035
H	5.952852	-3.68391	10.6245
H	3.666032	-7.914881	7.093977
H	3.956054	5.628112	-8.312231
H	2.508937	9.30561	9.75703
H	1.263626	7.62799	11.148821
H	1.775491	4.468693	-8.601202
H	-3.007528	-7.984152	-5.057743
H	9.965011	1.19639	8.645701
H	3.226441	-0.228446	-9.255342
H	8.449173	1.745577	11.384945
H	2.215933	3.263603	-11.544622
H	13.218497	3.709217	7.336307
H	-5.555427	-1.81278	10.697858
H	0.256477	-7.723269	2.413104
H	-0.389413	-10.371691	-0.919562
H	1.515111	-9.500946	3.531733
H	1.303123	-11.925984	0.017873
H	2.472818	-11.373371	2.174745
H	-6.612656	-5.457683	1.964926
H	-2.270688	-3.581462	3.227618
H	-6.165988	-3.100489	1.424362
H	-2.494829	-6.023102	2.974895
H	-4.685886	-7.03138	2.227772
H	2.988699	-8.878529	-1.682302
H	3.685674	-5.132576	-3.684742
H	0.865388	-8.962342	-2.899044
H	0.79062	-7.929762	-5.192994
H	2.305085	-5.93068	-5.549173
Au	6.836659	-2.597803	1.706525
H	-4.888813	9.195079	0.864674

H	7.321777	6.228782	-1.78807
H	7.000755	4.788926	0.917437
H	9.200176	-1.20677	-1.446589
H	-2.0362	1.43378	-1.026512
H	9.699835	-0.926174	0.215092
H	9.334714	1.648126	-1.529705
H	2.599222	6.926658	-0.713672
H	10.29665	-3.148225	0.563327
H	-3.284148	9.555853	1.474133
H	8.611732	5.33191	-0.963022
H	8.538525	3.079604	-0.863834
H	7.710026	6.23047	5.212785
H	-2.639424	7.497826	-0.290197
H	6.725899	1.150992	-2.294022
H	6.468308	4.124587	-0.650583
H	10.166526	-3.63506	-1.198662
H	-2.08646	3.182908	-1.152867
H	6.692366	2.899935	-2.334662
H	-3.775668	1.781743	-2.585763
H	1.316731	7.348321	0.400497
H	1.006171	6.74593	-2.561924
H	-4.271573	7.019665	0.186102
H	6.123349	6.242483	5.994833
H	-4.642467	1.447191	-1.077727
H	-1.375739	-3.073134	-2.873217
H	2.859825	5.504058	-1.693941
H	5.63722	-3.593814	-3.977922
H	7.158248	8.105552	4.019212
H	-3.689293	3.947455	3.537943
H	-0.953859	0.325097	-4.915349
H	1.935429	4.659681	-2.925645
H	-0.061552	7.937648	-1.818322
H	3.255763	-3.790382	4.165866

H	-3.217453	0.868449	5.842491
H	-2.013981	3.167356	-4.840067
H	-0.95866	-1.434608	-3.408897
H	7.191744	0.242294	3.436649
H	-3.135634	2.60677	4.543451
H	1.283025	6.614623	5.507245
H	7.648785	-1.457023	5.871922
H	-2.295087	-2.122518	-5.227476
H	-0.317523	7.309856	5.46962
H	6.592492	8.977987	5.464179
H	-3.06042	0.203624	4.210636
H	-1.930487	0.767818	-3.497618
H	4.62878	-2.557474	-4.999416
H	7.715453	2.424065	4.959756
H	3.07178	-4.667225	2.663562
H	2.258311	8.981668	5.191539
H	2.72453	7.220394	-3.395198
H	-0.707601	-2.617741	-5.832872
H	4.310128	6.496069	-3.675783
H	0.591368	9.514011	4.815575
H	-5.152697	2.69465	5.74537
H	1.054523	-4.574262	4.975619
H	-3.257881	1.943278	-4.977823
H	-3.113481	-2.164402	5.309455
H	6.072682	-2.977037	-6.757167
H	9.684121	3.679962	3.990908
H	6.017527	0.313063	4.920406
H	7.341235	-2.092964	-5.88469
H	8.103262	0.242497	5.889921
H	7.26377	3.872598	5.865737
H	0.972225	-5.766401	3.661572
H	-4.610629	-1.257432	5.195503
H	5.303867	-0.627118	-6.281452

H	-4.87966	4.401726	6.13366
H	2.067996	-1.731278	5.424029
H	3.759844	-2.171733	5.089532
H	9.274122	5.089578	4.960187
H	6.200934	0.688545	-7.994908
H	4.130466	-0.941656	-7.5884
H	4.775269	3.431528	6.346936
H	2.389883	-2.351899	7.847225
H	5.321808	4.692518	8.976876
H	5.344664	1.826342	-6.972698
H	2.572206	-3.710642	6.732464
H	3.945547	2.585592	7.618203
H	-1.471954	0.890513	8.183555
H	-0.288909	-0.964882	8.648715
H	6.464539	4.153097	7.770844
H	0.97549	-0.050415	9.439693
H	-1.422313	1.508813	9.851453
H	-1.741588	-8.071019	-0.455673
H	-0.213688	-7.730983	-1.242974
H	-1.698191	-6.01252	0.625236
H	-0.053863	-6.256071	1.221949
H	-4.329265	-1.247407	3.20295
H	-2.30102	-0.265267	2.560471
H	-1.807678	-1.959197	2.441793
H	-4.59551	-1.171955	1.46836
H	5.676137	-7.225586	-2.082506
H	6.109343	-5.096532	-1.349383
H	4.573535	-7.338008	-0.708238
H	4.556269	-4.638577	-2.063116
**B1-B**			
Au	-0.05402	-0.018579	0.033042
Au	2.740092	0.002246	0.100308
Au	1.254146	2.453639	0.052068

Au	1.501815	-1.887949	-1.5113
Au	1.327414	0.274894	2.502766
Au	0.21627	4.810334	1.110113
Au	3.104855	4.503779	0.90289
Au	3.845707	2.321075	-1.07213
Au	-0.859332	2.090343	1.926189
Au	1.465206	0.921595	-2.258061
Au	1.657983	3.037141	2.774091
Au	5.586324	0.065285	0.080327
Au	4.165357	-2.34097	-0.013632
Au	4.149559	1.818238	1.730247
Au	1.544402	-2.221263	1.325039
Au	4.031489	-0.434861	-2.323572
Au	3.938252	-0.903473	2.458634
S	6.09897	-1.037319	-3.518663
Au	1.970395	5.754652	3.297304
Au	-0.607332	4.309233	3.751688
Au	4.165054	3.935198	3.655063
Au	0.096299	1.58627	4.624887
Au	3.140951	1.315572	4.413667
Au	1.827551	3.623733	5.491329
S	4.759041	6.115745	0.081233
S	-0.631825	5.980449	-0.808146
S	5.75822	-1.823901	3.784792
S	-2.375823	-0.826735	0.032852
S	7.77482	1.122168	0.193985
S	6.59295	4.093746	3.701557
S	0.38271	-3.710788	2.836094
S	0.004161	2.234873	-3.679096
S	-1.522002	-0.1057	5.156618
S	2.878524	4.839462	7.340586
S	4.251637	-4.835479	0.027273
Au	-1.167284	-3.044336	-0.379654

C	-2.990268	-0.922286	1.76132
Au	2.007327	-4.738211	-0.325839
C	4.332081	-5.533145	-1.720305
Au	4.73497	6.80313	2.405604
C	6.271318	5.129624	-0.258165
Au	7.046115	2.530664	1.974037
C	8.211696	2.144959	-1.291412
C	7.232913	3.272385	5.22508
Au	-0.262968	3.990415	-2.178126
C	0.470567	7.309903	-1.507622
C	-1.597877	1.435852	-4.092846
Au	-0.392032	-1.782775	4.043137
C	-1.700776	-0.534335	6.938937
C	1.535317	-4.689072	3.902711
Au	3.810154	6.141659	5.814384
C	4.221707	3.617273	7.725485
S	4.955175	7.74226	4.521989
C	6.74069	7.479974	4.906394
S	-0.227463	-5.108193	-0.752404
C	-0.964909	-6.323682	0.428471
S	-3.06258	2.389764	0.938379
S	1.402623	8.133214	3.160592
Au	-2.838597	4.729185	1.70823
C	-2.912621	2.487652	-0.883702
Au	-0.70925	7.340806	2.644184
C	0.855694	8.696559	4.868535
S	-3.008739	6.954721	2.363342
C	-3.52139	7.720431	0.761187
S	0.7386	-2.772823	-3.613423
S	3.929188	4.093163	-2.728515
Au	2.135747	-1.107152	-4.613408
C	-0.934113	-1.980603	-3.571205
Au	3.607706	2.157666	-4.271406

C	2.517698	5.20434	-2.923638
S	3.460244	0.43056	-5.810697
C	2.642516	0.956435	-7.380318
S	-2.365361	4.72922	5.389887
S	3.653464	-0.108739	6.313257
Au	-1.013628	3.417249	6.808236
C	-3.580281	3.445684	4.796177
Au	1.720979	1.175334	7.3295
C	2.683061	-1.644787	5.920163
S	-0.043989	2.119064	8.496515
C	0.513698	3.311096	9.789363
Au	6.776679	-2.128742	-1.777284
C	5.71755	-2.222119	-4.862822
S	7.752731	-3.455402	-0.095822
C	7.324667	5.963267	-1.00388
C	8.430608	6.493077	-0.116386
C	8.575563	7.871171	0.115974
C	9.114119	5.662359	0.793249
C	9.65125	8.319864	0.901792
C	10.497979	7.41851	1.557847
C	10.340318	6.035631	1.331619
C	-3.410962	9.242878	0.783503
C	-3.510882	9.870401	-0.586675
C	-4.411427	10.916545	-0.844021
C	-2.863951	9.319154	-1.712674
C	-4.286603	11.618449	-2.052028
C	-3.780982	10.998756	-3.192538
C	-2.884605	9.937104	-2.964894
C	9.404253	-2.611972	0.250284
C	9.870458	-1.717736	-0.908119
C	11.3405	-1.730751	-1.246565
C	11.980243	-0.534966	-1.611502
C	12.142931	-2.879519	-1.177136

C	13.319172	-0.544057	-2.01615
C	13.94646	-1.766712	-2.291848
C	13.300449	-2.965051	-1.952495
C	-4.258388	2.320761	-1.572457
C	-4.937295	3.625452	-1.943284
C	-6.32787	3.658296	-2.117047
C	-4.212897	4.806689	-2.172578
C	-4.86414	5.985286	-2.546176
C	-6.244258	5.990605	-2.758178
C	-6.977438	4.823028	-2.527337
C	7.184039	2.083157	-2.411878
C	7.687554	2.390466	-3.796587
C	7.160056	3.463296	-4.535061
C	8.892062	1.860904	-4.284366
C	7.093531	3.311306	-5.922692
C	9.087098	1.946368	-5.677526
C	8.087194	2.496989	-6.49945
C	1.800531	7.577021	-0.792997
C	1.964503	9.02177	-0.382822
C	0.961791	9.761132	0.263934
C	3.231031	9.627707	-0.366405
C	1.282567	10.989536	0.859096
C	2.561215	11.550606	0.672814
C	3.405106	10.999924	-0.354173
C	7.72723	7.986875	3.833277
C	8.544225	9.168468	4.28298
C	8.070618	10.477509	4.116983
C	9.833138	8.986867	4.801921
C	8.733672	11.550356	4.714496
C	9.883576	11.315719	5.474571
C	10.534627	10.080409	5.322063
C	-2.540444	2.438934	-4.742089
C	-3.891304	1.891112	-5.147508

C	-4.448025	0.704692	-4.634442
C	-4.895074	2.815284	-5.537028
C	-5.588817	0.231006	-5.333381
C	-6.399857	1.080692	-6.093377
C	-5.961052	2.40407	-6.330008
C	-1.863041	-2.506635	-4.679049
C	-2.807276	-3.534507	-4.102116
C	-4.173789	-3.263277	-3.977623
C	-2.370924	-4.837867	-3.836231
C	-4.952398	-3.994275	-3.080081
C	-3.258286	-5.685559	-3.156606
C	-4.36023	-5.108265	-2.472029
C	2.946767	-4.771859	3.348234
C	3.797191	-5.909475	3.859561
C	5.183352	-5.876514	3.624652
C	3.347734	-6.848909	4.791168
C	5.880392	-7.081906	3.570497
C	5.217468	-8.235147	4.024673
C	4.085995	-8.053639	4.841369
C	0.765371	7.592429	5.915261
C	-0.165819	7.830839	7.083754
C	-0.014584	7.043774	8.2404
C	-1.432536	8.422159	6.929203
C	-0.904451	7.225801	9.308751
C	-2.015328	8.072357	9.196935
C	-2.171523	8.845698	8.031347
C	6.753126	-0.298637	4.04202
C	2.913524	6.350454	-3.863499
C	2.865529	5.899591	-5.308159
C	1.652091	5.451931	-5.854253
C	4.039195	5.640774	-6.0235
C	1.539597	5.215246	-7.222666
C	3.94932	5.234852	-7.36222

C	2.703478	5.206362	-8.003108
C	7.691629	-0.473347	5.273164
C	9.143275	-0.622255	4.858751
C	9.994058	-1.575643	5.442611
C	9.688905	0.165254	3.827953
C	11.159374	-1.955435	4.77123
C	11.80313	-0.996042	3.977444
C	11.047273	0.087933	3.506912
C	6.923098	-2.189624	-5.818295
C	7.982577	-3.157831	-5.351721
C	9.299137	-2.741572	-5.110277
C	7.716801	-4.528492	-5.193317
C	10.27435	-3.677537	-4.742918
C	9.843026	-4.900563	-4.203823
C	8.487575	-5.275893	-4.30541
C	-5.028105	3.712496	5.26401
C	-5.886613	3.693992	4.029547
C	-6.469488	4.844101	3.473694
C	-6.369018	2.476639	3.497461
C	-6.867831	4.748325	2.123267
C	-7.144992	3.523567	1.510356
C	-6.697399	2.349368	2.150331
C	-2.845669	-1.552144	7.099914
C	-4.146062	-0.846976	7.391215
C	-5.239975	-0.950811	6.520343
C	-4.3745	-0.240611	8.637084
C	-6.346918	-0.110764	6.703415
C	-6.390957	0.768062	7.790642
C	-5.376369	0.724557	8.757185
C	8.77349	3.257714	5.305895
C	9.380868	4.034698	6.445245
C	10.134242	3.394129	7.444673
C	8.902722	5.30596	6.828757

C	10.841423	4.229482	8.337458
C	9.69711	6.203408	7.532749
C	10.550668	5.591282	8.48096
C	1.136486	1.237806	-7.363092
C	0.224597	0.166839	-7.934327
C	0.621981	-0.734915	-8.930867
C	-1.129561	0.163211	-7.556399
C	-2.030572	-0.776648	-8.059395
C	-0.288979	-1.640959	-9.486084
C	-1.60923	-1.692723	-9.026973
C	3.263437	-2.839177	6.675187
C	2.244371	-3.864565	7.144128
C	0.884272	-3.570509	7.356197
C	2.706251	-5.074078	7.693495
C	1.802396	-6.121652	7.89005
C	-0.005193	-4.547868	7.818551
C	0.456859	-5.828986	8.136587
C	5.088727	4.031639	8.921191
C	5.746833	2.795163	9.477661
C	6.867296	2.211161	8.859292
C	5.554611	2.384054	10.811355
C	7.132084	0.868682	9.189958
C	6.614789	0.294794	10.359445
C	5.715048	1.036913	11.145238
C	1.692101	2.747022	10.580489
C	1.806823	3.249869	12.001389
C	2.528165	2.50573	12.946602
C	1.263587	4.476963	12.409818
C	2.639641	2.95346	14.264198
C	1.512191	4.987109	13.686809
C	1.950385	4.094419	14.683978
C	-1.11249	-7.656461	-0.300996
C	0.159458	-8.485775	-0.270781

C	0.997108	-8.526272	0.855605
C	0.62277	-9.091708	-1.447975
C	2.234504	-9.169877	0.805866
C	1.756069	-9.910686	-1.434878
C	2.557662	-9.973567	-0.291106
C	-4.367937	-1.58926	1.781537
C	-4.361613	-3.07494	2.082886
C	-5.402052	-3.885852	1.603656
C	-3.422666	-3.659668	2.943548
C	-5.338721	-5.275393	1.721959
C	-4.341369	-5.85952	2.510136
C	-3.42442	-5.037929	3.170978
C	4.421277	-4.683947	-2.971999
C	3.600538	-5.290313	-4.132416
C	2.693344	-6.350299	-3.958752
C	3.470153	-4.585183	-5.342368
C	2.091829	-6.956308	-5.066126
C	2.471953	-6.571741	-6.350449
C	2.982416	-5.267126	-6.467112
H	-4.284849	6.898459	-2.699196
H	14.930346	-1.782726	-2.764246
H	-6.906662	2.747163	-1.943834
H	13.830823	0.394955	-2.235238
H	-0.053391	9.368999	0.361363
H	-4.883215	12.528335	-2.17469
H	11.703157	-3.810773	-0.808312
H	11.350207	7.798063	2.124682
H	13.799466	-3.924611	-2.098942
H	-4.976504	11.358812	-0.019125
H	-3.84002	11.484986	-4.167644
H	-5.444383	1.339098	9.656441
H	11.433224	0.409272	-1.551412
H	3.774672	-5.301574	7.637196

H	-0.240313	-6.613584	8.435044
H	11.127717	6.191547	9.187608
H	7.287742	2.637932	7.947034
H	-3.06971	-0.772237	-7.71402
H	3.014524	1.576472	12.641695
H	9.775132	9.389776	1.080713
H	0.775854	5.118297	11.6717
H	8.302812	12.552427	4.663169
H	2.186115	-7.121673	8.107756
H	6.829711	-0.749703	10.592848
H	7.879377	0.316224	8.614325
H	7.955502	8.589549	-0.425437
H	7.13181	10.642895	3.582645
H	0.561389	11.484764	1.510603
H	3.148083	2.325865	14.999768
H	1.124663	5.970651	13.960576
H	2.001874	4.413459	15.726794
H	10.384027	12.139396	5.987356
H	-3.121801	4.822637	-2.067026
H	-6.749296	6.906868	-3.068763
H	11.322888	-3.385388	-4.665969
H	-8.05978	4.818612	-2.671314
H	8.860657	4.596113	0.800724
H	10.557775	-5.552711	-3.698086
H	11.52528	9.929632	5.756807
H	10.970725	5.30629	1.8435
H	-2.345254	9.477304	-3.796242
H	-2.208949	8.456141	-1.574418
H	9.577396	-1.701562	-5.298815
H	10.281941	7.991052	4.802715
H	9.590277	-2.227299	6.223221
H	2.760239	12.547524	1.076013
H	6.408902	4.092246	-4.044753

H	11.701745	-2.847308	5.092942
H	-3.850151	-0.01122	-4.063305
H	9.536756	1.218478	-3.681702
H	-6.281558	5.827605	3.913054
H	4.036262	9.023534	-0.809774
H	8.15239	-6.251569	-3.950013
H	-7.171098	5.66366	1.606437
H	5.628594	-4.980813	3.182373
H	6.722749	-4.900089	-5.469478
H	4.357321	11.463744	-0.616201
H	-5.955737	-0.775898	-5.115188
H	0.874472	6.420878	8.364925
H	12.816737	-1.161245	3.609301
H	0.749072	5.462425	-5.237561
H	6.413495	3.904401	-6.532916
H	-4.56743	-2.339728	-4.408262
H	6.89239	-7.12809	3.166068
H	9.955725	1.46722	-6.134759
H	-5.159264	-1.564349	5.619567
H	-7.478379	3.507624	0.471832
H	8.156023	2.384352	-7.583002
H	-1.379661	-5.1771	-4.140073
H	5.011412	5.801317	-5.55421
H	-1.672079	8.871344	5.959172
H	1.651623	-0.736151	-9.299322
H	2.378655	-6.772686	5.28072
H	9.020139	0.8344	3.282061
H	-4.59108	3.868777	-5.601236
H	-6.092517	1.564784	4.042854
H	-7.27666	0.678056	-6.60475
H	0.464219	-2.626043	7.002208
H	11.482012	0.796364	2.79939
H	-0.774409	6.637424	10.220424

H	-6.866248	1.364413	1.710478
H	10.402983	2.338059	7.354847
H	-5.948556	-3.652275	-2.79497
H	0.563219	5.03225	-7.674448
H	0.045664	-2.329578	-10.2651
H	5.696905	-9.214203	3.968119
H	-7.173188	-0.134713	5.990058
H	-3.597837	-0.301565	9.4062
H	8.126368	5.737698	6.187412
H	4.892667	2.993397	11.438215
H	-6.595486	3.128529	-6.843313
H	-2.943111	-6.69962	-2.90007
H	-1.058195	-4.293516	7.954139
H	3.702315	-8.895509	5.422504
H	4.85951	5.08129	-7.94627
H	-3.032836	9.504192	7.907403
H	-2.661972	8.232372	10.062346
H	2.645628	5.023792	-9.077872
H	-4.909261	-5.694622	-1.732293
H	11.518188	3.764364	9.058899
H	-1.482215	0.905812	-6.839643
H	5.360666	0.652498	12.103674
H	-2.310167	-2.423845	-9.438124
H	9.4373	7.261737	7.595094
H	-7.216364	1.475391	7.889083
H	0.720956	-7.996759	1.769005
H	0.031399	-8.999666	-2.362702
H	2.892399	-9.134689	1.677137
H	2.040938	-10.459445	-2.334607
H	3.474862	-10.564397	-0.292259
H	-6.097163	-5.898652	1.244184
H	-2.639078	-3.050216	3.400524
H	-6.187011	-3.434503	0.990748

H	-2.664933	-5.472651	3.823887
H	-4.299967	-6.943483	2.633507
H	2.492714	-6.791132	-2.984039
H	3.87757	-3.588567	-5.466204
H	1.46208	-7.835558	-4.911614
H	2.140142	-7.122305	-7.231719
H	3.042818	-4.790132	-7.447937
Au	6.663354	-2.584468	1.750615
H	-4.168513	9.67899	1.451142
H	6.842861	6.794271	-1.539573
H	6.65932	4.709732	0.678566
H	9.349448	-2.047316	-1.826914
H	-2.240693	1.656834	-1.137645
H	9.532826	-0.687813	-0.726854
H	9.169338	1.714138	-1.613839
H	2.654281	7.303277	-1.418306
H	9.337564	-2.052882	1.187197
H	-2.428596	9.505957	1.218732
H	7.775377	5.321755	-1.781466
H	8.403174	3.171466	-0.958013
H	7.165117	8.227291	2.918158
H	-2.887798	7.281714	-0.017735
H	6.772155	1.063767	-2.446998
H	5.91221	4.295793	-0.872063
H	10.104488	-3.451102	0.343186
H	-2.388538	3.408355	-1.154042
H	6.324706	2.725009	-2.170554
H	-4.112248	1.740419	-2.495687
H	1.856341	6.934726	0.104287
H	0.603995	7.062708	-2.564977
H	-4.554428	7.388178	0.588862
H	8.39522	7.154039	3.57217
H	-4.932878	1.716987	-0.946095

H	-1.333455	-2.215495	-2.565112
H	2.277002	5.538989	-1.912774
H	5.54648	-3.214626	-4.462083
H	6.922252	7.699927	5.964069
H	-3.534541	3.509659	3.699981
H	-1.336555	0.618574	-4.776805
H	1.65579	4.653391	-3.321193
H	-0.161156	8.206101	-1.465861
H	3.462251	-3.803989	3.4685
H	-0.744644	-0.906968	7.325441
H	-2.052555	2.867732	-5.634286
H	-0.780772	-0.900903	-3.615998
H	7.357922	-0.079078	3.16549
H	-3.219088	2.45218	5.083264
H	1.775253	7.382681	6.300451
H	7.380354	-1.335273	5.879487
H	-2.438675	-1.666284	-5.093446
H	0.441078	6.661149	5.415583
H	6.825223	6.388045	4.788762
H	-1.929514	0.407524	7.452421
H	-2.027145	0.999159	-3.181469
H	4.813933	-1.830632	-5.3443
H	6.801434	2.268007	5.285361
H	2.86993	-4.914804	2.25704
H	1.59664	9.454038	5.166873
H	2.211495	7.187587	-3.719158
H	-1.264853	-2.917018	-5.50695
H	3.916014	6.722593	-3.605935
H	-0.098559	9.208295	4.712084
H	-5.333196	2.942094	5.987409
H	1.498179	-4.298073	4.923374
H	-2.686926	3.291875	-4.05665
H	-2.591591	-2.212563	7.9473

H	6.569564	-2.488479	-6.821813
H	9.134403	2.221124	5.350733
H	6.039804	0.46014	4.385672
H	7.32349	-1.170417	-5.905216
H	7.57976	0.412458	5.920409
H	6.809345	3.862055	6.049243
H	1.066544	-5.684699	3.913913
H	-2.923645	-2.186663	6.206142
H	3.201601	1.858142	-7.664183
H	-5.093165	4.69205	5.760144
H	1.641916	-1.436171	6.171281
H	2.732954	-1.78943	4.83199
H	9.173771	3.663027	4.360275
H	0.970218	2.160974	-7.947562
H	2.910473	0.161049	-8.086544
H	4.805619	3.430914	6.818202
H	4.021583	-3.33636	6.051575
H	4.455812	4.498482	9.691101
H	0.822355	1.487922	-6.337395
H	3.799822	-2.47644	7.568973
H	3.685555	2.682015	7.960634
H	-0.377358	3.437916	10.419971
H	1.615447	1.648141	10.609081
H	5.828034	4.784227	8.601104
H	2.624601	2.957562	10.033627
H	0.754686	4.26699	9.310604
H	-1.924149	-8.220785	0.194252
H	-1.429841	-7.487917	-1.33839
H	-1.944065	-5.908237	0.697171
H	-0.361087	-6.372583	1.338441
H	-4.967015	-1.080361	2.559577
H	-3.050883	0.116696	2.103443
H	-2.245696	-1.432912	2.382589

H	-4.888466	-1.405641	0.829785
H	4.05346	-3.661986	-2.77558
H	3.498094	-6.230221	-1.746549
H	5.476343	-4.591622	-3.263663
H	5.229085	-6.174605	-1.672943
**B1-C**			
Au	-0.020651	0.021488	0.027932
Au	2.784324	0.016491	0.038552
Au	1.309592	2.48173	-0.004987
Au	1.495726	-1.877531	-1.52877
Au	1.423026	0.327173	2.466138
Au	0.307726	4.855515	1.049557
Au	3.189639	4.528521	0.787586
Au	3.877168	2.321377	-1.179476
Au	-0.76797	2.150819	1.914737
Au	1.464713	0.925165	-2.303126
Au	1.77168	3.09006	2.702296
Au	5.620082	0.049992	-0.038406
Au	4.185286	-2.3515	-0.081606
Au	4.233608	1.833221	1.621718
Au	1.582224	-2.183558	1.309952
Au	4.013626	-0.468614	-2.407238
Au	4.020786	-0.877386	2.381576
S	5.856569	-1.123835	-3.907034
Au	2.101607	5.808836	3.191639
Au	-0.475311	4.384774	3.712165
Au	4.30144	3.981002	3.523612
Au	0.228592	1.666024	4.599636
Au	3.266817	1.374124	4.32985
Au	1.999473	3.70292	5.40946
S	4.772222	6.060721	-0.181736
S	-0.581963	5.835626	-1.027946
S	5.754351	-2.055584	3.618404

S	-2.296153	-0.917966	0.093374
S	7.820387	1.020374	0.034858
S	6.725813	4.117948	3.417498
S	0.363821	-3.637088	2.946286
S	-0.0807	2.118752	-3.742252
S	-1.183742	-0.035262	5.529829
S	3.141646	4.736324	7.320604
S	4.370352	-4.772435	-0.041532
Au	-1.117408	-3.013028	-0.330604
C	-2.697071	-0.959217	1.876491
Au	1.990406	-4.727713	-0.323431
C	4.803138	-5.28965	-1.762966
Au	4.874465	6.828773	2.206728
C	6.394884	5.196895	-0.295175
Au	7.228926	2.475939	1.807896
C	8.211761	2.039808	-1.478144
C	7.511059	3.533414	4.976906
Au	-0.220358	3.949714	-2.240389
C	0.531067	7.163601	-1.697146
C	-1.762214	1.486111	-4.151932
Au	-0.278019	-1.650509	4.055358
C	-3.009392	0.01198	5.34377
C	1.526147	-4.491712	4.113871
Au	4.008808	6.202674	5.607448
C	4.606923	3.598101	7.421346
S	5.110886	7.843918	4.306061
C	6.833134	7.318478	4.790031
S	-0.302678	-5.142905	-0.772499
C	-0.755482	-6.312327	0.603681
S	-2.949002	2.391354	1.020335
S	1.633322	8.206769	3.008288
Au	-2.802501	4.793501	1.693559
C	-2.918595	2.781246	-0.761749

Au	-0.582469	7.412585	2.57702
C	1.25122	8.664726	4.790461
S	-2.889033	7.047588	2.281325
C	-3.345584	7.830865	0.659153
S	0.866182	-2.722092	-3.659261
S	4.110398	4.020197	-2.890119
Au	2.045755	-1.129556	-4.668525
C	-0.839574	-2.026706	-3.626182
Au	3.580804	2.146342	-4.367052
C	2.752101	5.21033	-3.051738
S	3.254232	0.465611	-5.947699
C	2.057039	0.996915	-7.266318
S	-2.230959	4.934795	5.276342
S	3.761763	-0.025689	6.27019
Au	-0.918022	3.538798	6.745905
C	-3.330603	3.528088	4.749512
Au	1.85686	1.306686	7.273757
C	2.830918	-1.580741	5.942956
S	0.118788	2.318884	8.43703
C	0.823456	3.541288	9.624847
Au	6.778012	-2.182022	-1.905954
C	5.00225	-2.620074	-4.628844
S	7.912769	-3.361504	-0.257741
C	7.507111	6.134079	-0.816173
C	8.703383	6.19308	0.103546
C	8.584344	6.714634	1.401499
C	9.916426	5.579517	-0.243951
C	9.675179	6.635649	2.267885
C	10.963663	6.544193	1.741306
C	11.060663	5.819476	0.538363
C	-2.614548	9.14885	0.412913
C	-2.66751	9.627595	-1.014839
C	-2.970463	10.958266	-1.346483

C	-2.499611	8.742301	-2.105375
C	-2.664593	11.374364	-2.651798
C	-2.807777	10.517483	-3.73694
C	-2.451247	9.181313	-3.433589
C	9.437241	-2.317686	0.000972
C	10.014578	-1.810297	-1.325234
C	11.522326	-1.684663	-1.413877
C	12.068243	-1.060768	-2.546545
C	12.403833	-2.062127	-0.395305
C	13.428126	-1.156571	-2.82739
C	14.263236	-1.794386	-1.899164
C	13.782097	-1.877826	-0.570182
C	-4.283507	2.625708	-1.43078
C	-4.901329	3.989839	-1.644962
C	-4.352807	4.894894	-2.571172
C	-6.18566	4.273091	-1.158473
C	-6.7369	5.549122	-1.326635
C	-6.086583	6.502111	-2.114612
C	-4.830214	6.202826	-2.656484
C	7.174584	1.909205	-2.584744
C	7.524971	2.550838	-3.900789
C	8.02603	3.859386	-4.001617
C	7.420354	1.841206	-5.113829
C	8.090141	4.449137	-5.271708
C	7.402369	2.462621	-6.361422
C	8.090286	3.700214	-6.448329
C	1.629363	7.634154	-0.751176
C	1.930252	9.119703	-0.761414
C	1.067734	10.103809	-1.267181
C	3.021175	9.578853	0.003561
C	1.558195	11.40486	-1.455531
C	2.695392	11.841754	-0.775572
C	3.474372	10.893303	-0.097922

C	7.441694	8.193134	5.914954
C	8.708488	8.846842	5.432351
C	8.67989	9.960498	4.57553
C	9.972026	8.308149	5.726945
C	9.798248	10.227383	3.783619
C	11.054681	9.970802	4.358681
C	11.129437	9.025488	5.393998
C	-2.571535	2.612973	-4.813301
C	-3.763949	2.129197	-5.607459
C	-5.039381	1.982564	-5.044458
C	-3.606958	1.769021	-6.95389
C	-6.134493	1.652999	-5.849497
C	-5.962293	1.473463	-7.225215
C	-4.649142	1.177437	-7.670173
C	-1.567868	-2.251003	-4.964766
C	-3.022407	-2.570415	-4.732689
C	-3.980077	-1.559925	-4.563059
C	-3.482089	-3.894756	-4.789246
C	-5.28714	-1.890532	-4.193464
C	-4.700121	-4.23838	-4.20031
C	-5.639116	-3.22537	-3.966753
C	3.024505	-4.348902	3.812089
C	3.791087	-5.638708	4.038896
C	3.131095	-6.84713	4.26295
C	5.193882	-5.641549	4.133869
C	3.854842	-7.987784	4.624663
C	5.194107	-7.885404	4.991093
C	5.898997	-6.827945	4.340436
C	0.925473	7.497631	5.72284
C	0.121239	7.833604	6.947129
C	-1.164794	8.392907	6.861096
C	0.63251	7.617032	8.238866
C	-1.775311	8.852545	8.031052

C	-1.515291	8.13928	9.211036
C	-0.2635	7.504862	9.312282
C	6.452958	-0.3592	4.033944
C	2.653388	5.725847	-4.489323
C	3.600791	6.866584	-4.793534
C	3.804416	7.915164	-3.88387
C	4.276196	6.92148	-6.021109
C	4.743628	8.90747	-4.167672
C	5.281199	7.878506	-6.228041
C	5.699093	8.70222	-5.161681
C	7.483835	-0.345111	5.158899
C	8.727212	-1.047395	4.68264
C	9.481151	-1.971893	5.417205
C	9.463824	-0.436298	3.623792
C	10.251831	-2.840268	4.61153
C	11.01972	-2.295536	3.579601
C	10.533001	-1.097589	3.011255
C	5.740962	-3.124563	-5.883199
C	5.161615	-2.61708	-7.17864
C	5.754226	-1.553779	-7.872283
C	3.879465	-3.015015	-7.611509
C	5.165225	-1.080087	-9.057481
C	3.940223	-1.589725	-9.510751
C	3.429253	-2.759273	-8.907488
C	-4.667017	3.425004	5.478499
C	-5.700538	2.820758	4.550219
C	-6.363641	1.632875	4.893773
C	-5.934306	3.351502	3.270556
C	-7.137298	0.953293	3.948368
C	-7.58951	1.653345	2.824013
C	-7.015474	2.894416	2.515693
C	-3.704032	-1.09506	6.122765
C	-3.430473	-1.14199	7.609811

C	-3.539043	-2.359645	8.296727
C	-3.070041	-0.004236	8.346876
C	-3.344024	-2.422479	9.678643
C	-3.045738	-1.262831	10.400665
C	-3.045515	-0.027927	9.741278
C	9.004586	3.31703	4.769211
C	9.843545	3.239922	6.025352
C	11.224421	2.990197	5.913387
C	9.363415	3.538619	7.30904
C	12.013264	3.078645	7.061422
C	10.078048	3.055019	8.422979
C	11.436035	2.717021	8.284862
C	0.661004	0.373708	-7.136018
C	-0.010937	-0.054924	-8.429225
C	-1.339787	-0.500528	-8.391891
C	0.727208	-0.369614	-9.577716
C	0.081479	-0.491452	-10.80889
C	-1.944791	-0.742662	-9.64345
C	-1.299713	-0.289681	-10.83909
C	3.170256	-2.595717	7.033991
C	2.482655	-2.211152	8.323776
C	3.194108	-1.999911	9.506861
C	1.084125	-2.30432	8.402463
C	0.410177	-1.658112	9.435241
C	2.508892	-1.359613	10.557365
C	1.164077	-0.980477	10.40093
C	5.495074	3.868732	8.653421
C	5.797898	2.5896	9.405877
C	4.93601	2.186047	10.434054
C	6.61388	1.613293	8.821633
C	5.304795	1.106649	11.238025
C	6.552312	0.517752	11.020831
C	6.99093	0.551581	9.671287

C	0.753483	2.973303	11.041786
C	1.758013	3.607855	11.973883
C	2.254524	4.912014	11.806424
C	2.252684	2.898599	13.095855
C	3.028275	5.398278	12.871368
C	3.422596	3.354208	13.745553
C	4.030992	4.608006	13.40229
C	0.484484	-6.820798	1.342472
C	0.757423	-8.30054	1.36999
C	2.072244	-8.751333	1.617834
C	-0.083258	-9.297541	0.828364
C	2.207103	-10.093503	2.016226
C	0.049742	-10.64916	1.159683
C	1.318059	-11.08433	1.589607
C	-4.202792	-1.021216	2.137932
C	-4.681045	-2.434613	2.426731
C	-5.945734	-2.883615	2.015773
C	-3.890855	-3.354001	3.158364
C	-6.203936	-4.258703	2.078444
C	-5.676625	-5.020995	3.117121
C	-4.367634	-4.631105	3.48244
C	4.588022	-6.795918	-1.973017
C	3.271775	-7.102962	-2.649001
C	2.328587	-7.955263	-2.051238
C	2.850756	-6.419165	-3.803107
C	1.027954	-7.999897	-2.561926
C	0.865325	-7.754787	-3.933701
C	1.839675	-6.97847	-4.590446
H	-7.713853	5.776512	-0.894656
H	15.332154	-1.892967	-2.100471
H	-3.349334	4.691228	-2.974317
H	13.81572	-0.827214	-3.793151
H	0.134426	9.834769	-1.76446

H	-2.722578	12.452435	-2.846299
H	12.026428	-2.372214	0.579758
H	11.842798	6.731938	2.359198
H	14.467615	-2.133344	0.241217
H	-3.091755	11.702525	-0.555318
H	-2.79619	10.888566	-4.762687
H	-2.836132	0.891375	10.291519
H	11.394746	-0.605476	-3.277956
H	0.516337	-2.70851	7.560094
H	0.665891	-0.391836	11.173564
H	12.041957	2.450259	9.151207
H	4.081576	2.80689	10.71133
H	0.669549	-0.535203	-11.72939
H	1.796273	5.603992	11.098628
H	9.55553	6.990425	3.296551
H	1.836116	1.924898	13.360027
H	9.71711	10.917513	2.94091
H	-0.676288	-1.582981	9.419943
H	6.95682	-0.227989	11.707422
H	4.727294	0.888906	12.139133
H	7.600724	7.032764	1.762431
H	7.708841	10.381323	4.295875
H	0.937562	12.127546	-1.99073
H	3.205547	6.484744	12.856215
H	3.917093	2.678239	14.448609
H	4.889037	4.997671	13.948864
H	11.963064	10.402063	3.934946
H	-6.696986	3.533245	-0.538961
H	-6.51205	7.500165	-2.233807
H	5.618273	-0.233835	-9.578865
H	-4.31047	6.943598	-3.263765
H	10.008259	5.041728	-1.190558
H	3.521687	-1.231506	-10.46212

H	12.095257	8.737422	5.812162
H	12.036885	5.498895	0.167382
H	-2.328541	8.443606	-4.230928
H	-2.264839	7.692343	-1.893633
H	6.714814	-1.149787	-7.534445
H	10.044045	7.439101	6.387128
H	9.076101	-2.356092	6.360823
H	3.035687	12.87421	-0.873506
H	8.125583	4.487014	-3.116094
H	10.571797	-3.793212	5.046711
H	-5.202931	2.19585	-3.987879
H	7.018313	0.825477	-5.051316
H	-6.148177	1.165254	5.85819
H	3.642926	8.850349	0.533673
H	2.511023	-3.223785	-9.284298
H	-7.53678	-0.036861	4.176722
H	2.042204	-6.854666	4.133223
H	3.370487	-3.793182	-7.031656
H	4.371743	11.198145	0.44394
H	-7.140156	1.680273	-5.423326
H	-1.572707	8.667215	5.88432
H	11.788579	-2.886352	3.077995
H	3.193273	8.010595	-2.986212
H	8.412334	5.494371	-5.325403
H	-3.699914	-0.51088	-4.660472
H	3.299162	-8.898117	4.867944
H	7.174349	1.875666	-7.254483
H	-3.790759	-3.266689	7.741506
H	-8.334671	1.210334	2.160828
H	8.24803	4.192176	-7.410637
H	-2.767668	-4.685184	-5.039104
H	4.05249	6.188788	-6.799239
H	1.656063	7.247194	8.346947

H	-1.906194	-0.602291	-7.467671
H	5.744629	-4.736897	3.864316
H	9.005268	0.416341	3.084967
H	-2.605355	1.792859	-7.39286
H	-5.357893	4.214515	2.91554
H	-6.823771	1.247332	-7.860671
H	4.275752	-2.136403	9.549408
H	11.050262	-0.626523	2.172439
H	-2.685766	9.453229	7.958675
H	-7.306572	3.426783	1.609377
H	11.686628	2.932635	4.925283
H	-6.025749	-1.097487	-4.063737
H	4.883485	9.707159	-3.4347
H	-3.008441	-0.995827	-9.676173
H	5.731444	-8.753683	5.378389
H	-3.388889	-3.38631	10.189048
H	-2.933323	0.95437	7.841578
H	8.332293	3.860825	7.454854
H	7.082458	1.746184	7.846946
H	-4.476492	0.838388	-8.694001
H	-4.97678	-5.287091	-4.078522
H	3.067326	-1.031427	11.436213
H	6.991539	-6.804985	4.353859
H	5.865066	7.84269	-7.150906
H	0.058455	7.072112	10.261907
H	-2.170033	8.225575	10.079261
H	6.542221	9.384446	-5.283115
H	-6.633714	-3.472369	-3.590987
H	13.100871	3.030194	6.950627
H	1.815923	-0.363056	-9.548329
H	7.772708	-0.138938	9.343831
H	-1.851701	-0.267379	-11.781642
H	9.58301	2.998961	9.394261

H	-2.912231	-1.311319	11.482883
H	2.857824	-8.034932	1.870104
H	-1.061858	-8.98926	0.444648
H	3.18119	-10.407103	2.406256
H	-0.714758	-11.362717	0.84591
H	1.507671	-12.134128	1.82073
H	-7.109006	-4.620436	1.576897
H	-2.84174	-3.109479	3.358961
H	-6.556729	-2.24598	1.371346
H	-3.753604	-5.279193	4.113345
H	-6.046564	-6.023064	3.338461
H	2.544886	-8.402627	-1.078263
H	3.509943	-5.670035	-4.253108
H	0.247424	-8.514679	-1.997939
H	-0.051982	-8.043503	-4.448979
H	1.68457	-6.646452	-5.618083
Au	6.744548	-2.616361	1.598027
H	-2.986102	9.933944	1.088691
H	7.093037	7.149049	-0.93062
H	6.645357	4.785443	0.688025
H	9.703712	-2.489107	-2.138942
H	-2.194522	2.072286	-1.192075
H	9.544662	-0.842841	-1.563218
H	9.188546	1.659261	-1.811401
H	2.562249	7.083561	-0.92543
H	9.143034	-1.435712	0.576995
H	-1.550035	8.999642	0.681355
H	7.816124	5.826506	-1.822848
H	8.347938	3.074298	-1.141949
H	7.642588	7.571204	6.798646
H	-3.107479	7.102528	-0.122121
H	6.974831	0.84215	-2.748681
H	6.225019	4.35429	-0.979105

H	10.127264	-2.965507	0.549745
H	-2.51679	3.784922	-0.916558
H	6.217032	2.326908	-2.221512
H	-4.128931	2.118413	-2.391822
H	1.3392	7.353788	0.277395
H	0.919429	6.820752	-2.659802
H	-4.436097	7.95977	0.689347
H	6.718031	8.963589	6.224125
H	-4.945188	1.986607	-0.829271
H	-1.347308	-2.587035	-2.824393
H	2.999419	6.001247	-2.340133
H	4.964558	-3.381376	-3.847291
H	6.772282	6.280704	5.121711
H	-3.520931	3.736118	3.687132
H	-1.583746	0.668206	-4.861799
H	1.814743	4.726298	-2.731969
H	-0.172664	7.975416	-1.907576
H	3.478315	-3.567394	4.426042
H	-3.308418	0.992458	5.733368
H	-1.897116	3.162578	-5.489239
H	-0.802691	-0.974899	-3.323937
H	6.883428	0.071011	3.124889
H	-2.749419	2.603404	4.77864
H	1.862068	7.004501	6.019519
H	7.07234	-0.816364	6.063848
H	-1.442224	-1.366639	-5.603588
H	0.356263	6.741145	5.153644
H	7.444417	7.386243	3.88731
H	-3.276624	-0.011659	4.286419
H	-2.263525	1.071453	-3.265929
H	3.973293	-2.323644	-4.880029
H	6.992713	2.635988	5.337721
H	3.154827	-4.029888	2.763637

H	2.141607	9.203752	5.148078
H	2.810216	4.895012	-5.192359
H	-1.091964	-3.091844	-5.49244
H	1.61185	6.065714	-4.646767
H	0.423065	9.381346	4.736951
H	-4.580877	2.835028	6.402989
H	1.282349	-4.150399	5.126956
H	-2.882051	3.322047	-4.040218
H	-3.457321	-2.069955	5.675383
H	6.809878	-2.870121	-5.821072
H	9.157719	2.396303	4.183059
H	5.602067	0.21781	4.408823
H	5.685838	-4.226236	-5.885652
H	7.701585	0.707763	5.397526
H	7.322341	4.342817	5.697208
H	1.240025	-5.563456	4.064593
H	-4.78965	-0.962164	5.961144
H	2.021166	2.092045	-7.2547
H	-4.988232	4.436535	5.779323
H	1.755569	-1.370444	5.893265
H	3.157972	-1.910628	4.951152
H	9.38539	4.132116	4.129387
H	0.006531	1.086293	-6.609284
H	2.565557	0.684039	-8.185129
H	5.146434	3.641786	6.47154
H	2.826741	-3.591034	6.701917
H	4.983229	4.569294	9.330354
H	0.751858	-0.492183	-6.430986
H	4.260437	-2.656388	7.165314
H	4.169339	2.592914	7.50692
H	0.248287	4.468243	9.50937
H	-0.268009	3.090061	11.440748
H	6.425414	4.368142	8.342161

H	0.945915	1.890219	11.000493
H	1.85606	3.75303	9.324365
H	1.374957	-6.274224	0.944594
H	0.407204	-6.488139	2.38991
H	-1.310475	-7.107753	0.093281
H	-1.444184	-5.784862	1.271593
H	-4.466428	-0.362818	2.980018
H	-2.275888	-0.026986	2.279921
H	-2.14219	-1.775907	2.344425
H	-4.750635	-0.616091	1.274148
H	5.413763	-7.168735	-2.602855
H	5.860562	-5.01331	-1.886643
H	4.65743	-7.330097	-1.014867
H	4.208461	-4.692182	-2.459584
**B1-D**			
Au	0.011798	-0.063816	-0.019463
Au	2.806191	-0.057948	0.038499
Au	1.333824	2.401054	-0.033175
Au	1.552041	-1.959563	-1.547192
Au	1.403011	0.250277	2.442223
Au	0.312648	4.775574	1.000995
Au	3.197561	4.451299	0.77784
Au	3.92089	2.242499	-1.174304
Au	-0.766229	2.074437	1.841566
Au	1.527972	0.843015	-2.335823
Au	1.749916	3.013559	2.680618
Au	5.652649	-0.011168	0.008566
Au	4.217944	-2.410309	-0.053004
Au	4.231172	1.768716	1.642821
Au	1.601818	-2.258379	1.28252
Au	4.086469	-0.528655	-2.394162
Au	4.007058	-0.943158	2.403227
S	6.148986	-1.112895	-3.596586

Au	2.078736	5.734451	3.171106
Au	-0.50576	4.311026	3.641301
Au	4.274515	3.909137	3.542556
Au	0.186302	1.592867	4.553069
Au	3.22803	1.304131	4.325325
Au	1.931122	3.632009	5.380778
S	4.789985	6.058785	-0.088692
S	-0.670584	5.725191	-1.023996
S	5.732435	-1.908246	3.790929
S	-2.242648	-1.027915	0.010186
S	7.902193	1.066418	0.210905
S	6.643248	4.067861	3.474039
S	0.353374	-3.661932	2.869447
S	0.170854	2.038709	-3.968214
S	-1.175405	-0.141414	5.43171
S	2.916998	4.901878	7.316292
S	4.379838	-4.822889	-0.091504
Au	-1.025552	-3.173274	-0.384929
C	-2.792182	-0.91343	1.765621
Au	2.084536	-4.809425	-0.332899
C	4.751473	-5.120595	-1.885655
Au	4.870446	6.737936	2.154365
C	6.38458	5.174912	-0.359266
Au	7.187217	2.434227	1.870021
C	8.536721	1.972589	-1.287594
C	7.157282	3.401416	5.121984
Au	-0.104168	3.751148	-2.309786
C	0.362604	7.197096	-1.495202
C	-1.582778	1.509319	-4.203837
Au	-0.32203	-1.762579	4.011715
C	-3.01	-0.24048	5.573693
C	1.486475	-4.765573	3.860603
Au	3.954118	6.15853	5.636766

C	4.247523	3.663958	7.698976
S	5.232549	7.643049	4.258551
C	4.469268	9.327582	4.233832
S	-0.148635	-5.282453	-0.80468
C	-0.831254	-6.392071	0.510319
S	-2.99603	2.234288	0.918331
S	1.512011	8.137215	3.147669
Au	-2.813718	4.689272	1.729677
C	-2.994884	2.610327	-0.873352
Au	-0.62036	7.30312	2.618728
C	1.069091	8.638783	4.891119
S	-2.949649	6.913899	2.393201
C	-3.564568	7.775552	0.860887
S	1.165296	-2.990859	-3.771323
S	4.442969	3.904512	-2.912232
Au	2.087654	-1.136713	-4.632977
C	-0.642778	-3.0969	-4.146857
Au	3.688613	2.090151	-4.346989
C	3.02816	5.067954	-3.073672
S	2.983401	0.579916	-5.944774
C	4.399098	-0.080082	-6.913299
S	-2.215205	4.831963	5.284434
S	3.722896	-0.090817	6.251317
Au	-0.982221	3.483337	6.682847
C	-3.523135	3.577191	4.865512
Au	1.769921	1.239718	7.246134
C	2.803809	-1.653028	5.87388
S	-0.016396	2.207852	8.415236
C	0.825813	3.374904	9.580187
Au	6.802371	-2.219459	-1.695105
C	5.460639	-2.495713	-4.642865
S	7.799746	-3.550296	0.024814
C	7.426298	6.144816	-0.914744

C	7.8816	7.145146	0.120007
C	8.424036	6.714742	1.341917
C	7.751541	8.523868	-0.075893
C	8.423963	7.561016	2.447241
C	8.370351	8.944188	2.199135
C	8.246814	9.415478	0.881427
C	-2.619001	8.79569	0.228538
C	-3.161428	10.180505	-0.064475
C	-2.366873	11.055144	-0.827802
C	-4.346565	10.694986	0.478842
C	-2.854045	12.305071	-1.209602
C	-3.957689	12.856804	-0.553063
C	-4.750574	12.011129	0.225753
C	9.354429	-2.518501	0.12833
C	10.575558	-3.198024	-0.532024
C	10.998277	-2.382647	-1.742075
C	11.147816	-2.961982	-3.00784
C	10.939082	-0.979517	-1.676812
C	11.420407	-2.162589	-4.125982
C	11.509681	-0.773012	-4.005875
C	11.332239	-0.184877	-2.748249
C	-4.41764	2.821994	-1.439069
C	-4.484903	4.149959	-2.152476
C	-5.082062	5.273802	-1.562162
C	-3.778168	4.339073	-3.348993
C	-3.772687	5.577874	-3.989478
C	-4.185947	6.718017	-3.297098
C	-4.971698	6.539066	-2.152066
C	7.52268	2.17845	-2.409545
C	8.19604	2.529391	-3.713813
C	7.868481	1.869763	-4.906573
C	9.09661	3.599907	-3.82123
C	8.76873	1.86235	-5.96978

C	9.512685	4.0208	-5.086288
C	9.588642	3.008176	-6.071673
C	1.432923	7.512541	-0.453727
C	1.991579	8.90961	-0.447596
C	1.268795	9.999534	0.058174
C	3.30632	9.133221	-0.887203
C	1.907675	11.231603	0.203925
C	3.298236	11.318574	0.165822
C	3.904857	10.391444	-0.721211
C	5.443382	10.283545	4.952274
C	5.093409	10.64364	6.383598
C	6.036667	11.330344	7.16845
C	3.862362	10.340904	6.985197
C	5.803528	11.519968	8.53133
C	4.492004	11.520713	9.013516
C	3.538449	10.827198	8.258745
C	-1.943374	0.221926	-3.495188
C	-3.31786	-0.329898	-3.816272
C	-4.017243	0.033908	-4.976369
C	-3.777493	-1.450557	-3.10581
C	-5.335126	-0.394969	-5.1637
C	-5.9493	-1.163366	-4.17445
C	-5.106888	-1.854093	-3.283547
C	-0.873532	-4.083257	-5.290011
C	-0.812282	-3.437881	-6.660204
C	-1.425819	-2.199943	-6.915928
C	-0.482847	-4.201245	-7.789478
C	-1.046468	-1.499022	-8.06749
C	-0.032513	-3.512619	-8.927354
C	-0.243175	-2.130132	-9.026773
C	2.954468	-4.776715	3.40467
C	3.496323	-6.142554	3.061367
C	2.819562	-7.000243	2.18157

C	4.709058	-6.582946	3.603691
C	3.469246	-8.086475	1.600728
C	4.781369	-8.383007	1.984614
C	5.301503	-7.758705	3.127983
C	0.489962	7.529468	5.755457
C	-0.251102	7.944972	7.001733
C	-0.460037	6.976063	8.001375
C	-0.510369	9.273778	7.366591
C	-1.176264	7.308536	9.153618
C	-1.734315	8.588722	9.295715
C	-1.501726	9.558589	8.309977
C	6.819769	-0.446653	4.120186
C	3.174683	5.98719	-4.30423
C	1.859444	6.211037	-5.020109
C	1.149745	7.393577	-4.766246
C	1.131662	5.116953	-5.491942
C	0.110585	7.739631	-5.633978
C	0.118361	5.486759	-6.410603
C	-0.164213	6.851455	-6.681499
C	8.083731	-0.895743	4.891684
C	9.316817	-1.008202	4.030323
C	9.945152	-2.241593	3.802877
C	9.772127	0.076724	3.270183
C	11.277888	-2.288654	3.396119
C	11.729394	-1.147434	2.696595
C	10.797094	-0.132683	2.34548
C	6.350122	-2.8248	-5.831774
C	5.659008	-3.618698	-6.92411
C	4.262662	-3.716011	-7.039267
C	6.426249	-4.402443	-7.802073
C	3.691854	-4.118971	-8.251005
C	4.471589	-4.825748	-9.171153
C	5.833266	-5.01511	-8.90922

C	-4.553069	3.405939	5.998201
C	-5.964462	3.666873	5.523254
C	-6.298008	4.911415	4.965039
C	-7.000462	2.76798	5.799763
C	-7.583001	5.100682	4.457169
C	-8.388217	3.992328	4.192679
C	-8.213089	2.923005	5.097626
C	-3.404276	-1.58302	6.176794
C	-3.411428	-1.65044	7.691287
C	-3.301437	-0.532439	8.529662
C	-3.469943	-2.911529	8.31503
C	-3.531329	-0.672209	9.90393
C	-3.274945	-1.900959	10.512225
C	-3.298428	-3.041252	9.695383
C	8.532807	3.883771	5.555368
C	8.471388	5.326382	6.034974
C	9.577111	6.191752	5.96731
C	7.35834	5.832755	6.734869
C	9.3388	7.574274	6.001242
C	7.181948	7.199172	6.958954
C	8.231913	8.084734	6.687672
C	5.014543	0.991982	-7.819056
C	5.087984	0.674058	-9.302602
C	4.658685	-0.52824	-9.877733
C	5.536328	1.687408	-10.167736
C	5.567943	1.493564	-11.547711
C	4.919029	-0.808028	-11.223347
C	5.296085	0.227268	-12.080632
C	3.165117	-2.710016	6.915158
C	2.315078	-2.586728	8.163809
C	0.932577	-2.348433	8.08717
C	2.888941	-2.685528	9.437866
C	2.104897	-2.495951	10.581301

C	0.244194	-1.869534	9.204877
C	0.861136	-1.872542	10.462354
C	4.749017	3.678549	9.142827
C	5.715051	2.52552	9.262811
C	7.085543	2.701906	9.022971
C	5.256427	1.242462	9.595591
C	7.942884	1.59775	8.998649
C	7.420266	0.303606	9.102055
C	6.0539	0.125204	9.345908
C	-0.050931	3.568457	10.825476
C	0.240727	2.542143	11.896912
C	1.523824	2.442205	12.46013
C	-0.794861	1.869494	12.557722
C	1.787178	1.402921	13.357846
C	-0.476692	0.642449	13.164752
C	0.860424	0.361685	13.488763
C	-2.086077	-7.094196	-0.012426
C	-1.899984	-8.515876	-0.50578
C	-0.794053	-9.318874	-0.194166
C	-2.811745	-9.031131	-1.447283
C	-0.764295	-10.651141	-0.625758
C	-2.759434	-10.352113	-1.892125
C	-1.590833	-11.09895	-1.653242
C	-4.278077	-1.201795	1.932832
C	-4.647723	-2.617325	2.328367
C	-5.992218	-2.931121	2.599353
C	-3.718396	-3.61313	2.654697
C	-6.324022	-4.141728	3.213867
C	-5.369304	-5.181302	3.204696
C	-4.088931	-4.955364	2.65017
C	4.887279	-6.637028	-2.083945
C	3.693132	-7.28391	-2.755435
C	2.953351	-6.652671	-3.764825

C	3.297086	-8.569758	-2.358624
C	1.713575	-7.159957	-4.15689
C	1.251227	-8.368341	-3.630672
C	2.116974	-9.137032	-2.844955
H	-3.240992	5.684611	-4.938381
H	11.724571	-0.155649	-4.879776
H	-5.654226	5.151923	-0.638945
H	11.536525	-2.629504	-5.106299
H	0.212674	9.89751	0.309745
H	-2.258479	12.925563	-1.883664
H	10.741054	-0.496359	-0.716968
H	8.516804	9.649105	3.020612
H	11.297708	0.900779	-2.619857
H	-1.435075	10.687437	-1.265878
H	-4.276238	13.879298	-0.762293
H	-3.196433	-4.035141	10.134894
H	11.086868	-4.047517	-3.117024
H	3.939461	-2.97113	9.536192
H	0.321672	-1.523244	11.342956
H	8.096946	9.159769	6.815691
H	7.478637	3.705456	8.841992
H	5.864208	2.313288	-12.204994
H	2.317501	3.134361	12.169937
H	8.594149	7.176692	3.452105
H	-1.833516	2.090216	12.298126
H	6.602331	11.939581	9.148786
H	2.559722	-2.617824	11.567602
H	8.07685	-0.561182	8.988717
H	9.013918	1.743665	8.843904
H	8.580676	5.643739	1.502501
H	7.030426	11.528591	6.757623
H	1.333951	12.052635	0.645627
H	2.787574	1.298771	13.783558

H	-1.270053	-0.055092	13.440505
H	1.125894	-0.561125	14.006365
H	4.265698	11.855832	10.026652
H	-3.314242	3.472256	-3.829573
H	-4.067742	7.709589	-3.737229
H	2.615369	-4.013431	-8.397394
H	-5.418545	7.40511	-1.658826
H	7.397722	8.895473	-1.041421
H	4.026917	-5.223318	-10.085479
H	2.528839	10.674652	8.645834
H	8.269773	10.487889	0.677943
H	-5.662997	12.388897	0.691623
H	-4.991116	10.067077	1.095771
H	3.611038	-3.240837	-6.302147
H	3.094072	9.800998	6.42624
H	9.491442	-3.132892	4.24835
H	3.811124	12.241276	0.444116
H	7.090814	1.102213	-4.859591
H	11.890906	-3.183528	3.496304
H	-3.590082	0.750648	-5.67918
H	9.288547	4.23365	-2.950786
H	-5.567116	5.722773	4.945767
H	3.880091	8.306717	-1.318737
H	6.443826	-5.610216	-9.591104
H	-7.828955	6.0665	4.006273
H	1.808932	-6.74428	1.85856
H	7.5076	-4.469718	-7.659251
H	4.95186	10.518251	-1.009007
H	-5.912572	-0.01614	-6.009247
H	-0.128905	5.947858	7.831465
H	12.712824	-1.15751	2.219977
H	1.576989	8.113115	-4.060532
H	8.704654	1.136049	-6.780642

H	-1.986631	-1.682205	-6.134174
H	2.977468	-8.65576	0.809584
H	10.071572	4.949711	-5.211933
H	-3.246246	0.475012	8.114485
H	-9.304458	4.079447	3.607028
H	10.17847	3.176806	-6.975788
H	-0.251487	-5.264124	-7.675794
H	1.473691	4.086244	-5.40355
H	-0.211298	10.074473	6.683391
H	4.284925	-1.342348	-9.254158
H	5.201136	-5.992998	4.380077
H	9.204609	1.01244	3.253413
H	-3.163322	-1.906068	-2.327729
H	-6.825271	1.883435	6.415123
H	-7.007907	-1.419847	-4.242876
H	0.420658	-2.349117	7.122045
H	11.083891	0.658349	1.650089
H	-1.334484	6.557866	9.9305
H	-8.96476	2.131839	5.156238
H	10.505322	5.830911	5.512811
H	-1.373388	-0.468388	-8.215914
H	-0.346547	8.7302	-5.593812
H	4.700203	-1.801591	-11.618802
H	5.316801	-9.216205	1.524991
H	-3.590394	0.229626	10.518762
H	-3.544039	-3.808832	7.69556
H	6.471349	5.214383	6.884946
H	4.199638	1.099241	9.839525
H	-5.519605	-2.635561	-2.64134
H	0.429096	-4.058191	-9.753031
H	-0.77853	-1.508766	9.091727
H	6.259601	-8.084753	3.537845
H	-0.357103	4.714069	-7.020148

H	-1.882414	10.574324	8.428008
H	-2.390479	8.804226	10.141242
H	-0.865936	7.112802	-7.475369
H	0.128017	-1.575086	-9.88939
H	10.153111	8.250272	5.725353
H	5.825979	2.655265	-9.750011
H	5.63466	-0.875733	9.458761
H	5.406149	0.046567	-13.151414
H	6.21536	7.530703	7.348322
H	-3.22505	-1.99421	11.59798
H	-0.040027	-8.990843	0.523052
H	-3.671671	-8.414802	-1.726444
H	0.079795	-11.273167	-0.314511
H	-3.514184	-10.714646	-2.593464
H	-1.477256	-12.105811	-2.058766
H	-7.335203	-4.326403	3.577996
H	-2.654728	-3.393523	2.5176
H	-6.765139	-2.174284	2.438247
H	-3.338355	-5.74733	2.632026
H	-5.671322	-6.186678	3.508366
H	3.277468	-5.691924	-4.170952
H	3.907396	-9.117115	-1.635769
H	1.10214	-6.597462	-4.863476
H	0.27055	-8.75862	-3.908129
H	1.804003	-10.124558	-2.50029
Au	6.673368	-2.668582	1.743403
H	-1.738917	8.908609	0.889767
H	7.029	6.658375	-1.8025
H	6.711139	4.707175	0.577257
H	11.396956	-3.249728	0.201006
H	-2.502734	1.754567	-1.348621
H	10.347474	-4.231197	-0.831018
H	9.431705	1.437743	-1.651899

H	2.247479	6.781472	-0.535079
H	9.140694	-1.648466	-0.489158
H	-2.228381	8.365834	-0.704898
H	8.288491	5.542988	-1.255269
H	8.895766	2.936323	-0.899124
H	5.478207	11.225393	4.376339
H	-3.813186	6.987383	0.146001
H	6.937539	1.257738	-2.542048
H	6.152796	4.384126	-1.078607
H	9.517027	-2.246733	1.17456
H	-2.365052	3.488864	-1.049104
H	6.803784	2.954376	-2.113706
H	-4.661747	1.995008	-2.123045
H	0.975523	7.335269	0.533959
H	0.751899	7.035617	-2.506996
H	-4.505032	8.228869	1.196489
H	6.46452	9.87489	4.913388
H	-5.155008	2.797096	-0.62359
H	-1.09948	-3.452959	-3.212993
H	3.010111	5.645281	-2.141974
H	5.285275	-3.369783	-4.008003
H	4.384994	9.62832	3.186438
H	-4.001851	3.959958	3.950974
H	-2.22373	2.367017	-3.961677
H	2.099616	4.482531	-3.112774
H	-0.369102	8.015474	-1.556394
H	3.591622	-4.310488	4.17035
H	-3.306096	0.616737	6.188299
H	-1.852004	0.353031	-2.406199
H	-1.044099	-2.109549	-4.371432
H	7.090539	0.047189	3.181898
H	-3.019931	2.64042	4.600601
H	1.286296	6.810189	6.002338

H	7.893813	-1.842542	5.41666
H	-0.154258	-4.911126	-5.229221
H	-0.225484	6.955526	5.137346
H	3.521918	9.322285	4.75674
H	-3.454634	-0.109826	4.578687
H	-1.638189	1.369076	-5.294776
H	4.479018	-2.12401	-4.964473
H	7.120756	2.306941	5.06273
H	3.0493	-4.133283	2.511979
H	1.941189	9.086321	5.391835
H	3.619851	6.948321	-4.008582
H	-1.874703	-4.527967	-5.140375
H	3.87893	5.520846	-5.012283
H	0.337624	9.445688	4.747664
H	-4.480227	2.395678	6.425912
H	1.386734	-4.476552	4.913239
H	-1.160807	-0.51416	-3.732726
H	-2.757037	-2.372029	5.763537
H	6.713183	-1.884426	-6.283594
H	8.88113	3.224797	6.370965
H	6.221208	0.194989	4.78045
H	7.254887	-3.353856	-5.493323
H	8.282811	-0.144516	5.675944
H	6.381337	3.699506	5.825497
H	1.031076	-5.757322	3.74272
H	-4.422925	-1.824131	5.821043
H	5.139736	-0.473921	-6.211955
H	-4.305609	4.102684	6.815813
H	1.730422	-1.44611	5.824605
H	3.134673	-1.951752	4.86968
H	9.256307	3.791921	4.73198
H	6.029306	1.226199	-7.459324
H	3.972408	-0.922684	-7.465789

H	5.068612	3.806918	6.995138
H	3.006405	-3.704607	6.457229
H	3.903376	3.553132	9.834509
H	4.450497	1.931824	-7.701482
H	4.233584	-2.651447	7.166741
H	3.809354	2.67682	7.487071
H	1.02888	4.313378	9.05549
H	0.155083	4.57951	11.220008
H	5.235452	4.638311	9.37444
H	-1.114735	3.547832	10.547571
H	1.7821	2.909613	9.842156
H	-2.827879	-7.120433	0.805655
H	-2.554363	-6.487676	-0.802068
H	-1.024271	-5.775918	1.39714
H	-0.028106	-7.094453	0.754083
H	-4.674205	-0.50836	2.695094
H	-2.561609	0.115402	2.057375
H	-2.155073	-1.555767	2.390034
H	-4.814536	-0.938174	1.005957
H	5.785702	-6.822392	-2.700822
H	5.701661	-4.599216	-2.079441
H	5.075922	-7.128879	-1.119172
H	3.968042	-4.665216	-2.497006
**B1-E**			
Au	0.055062	-0.017184	0.031627
Au	2.849608	0.001345	0.078459
Au	1.365237	2.453985	0.04861
Au	1.59809	-1.882981	-1.529791
Au	1.454795	0.267695	2.492036
Au	0.336964	4.808206	1.121435
Au	3.221264	4.51132	0.883937
Au	3.948351	2.324596	-1.105082
Au	-0.735581	2.088202	1.927367

Au	1.557422	0.930651	-2.277307
Au	1.789473	3.02885	2.769368
Au	5.685695	0.061034	0.038255
Au	4.272193	-2.342577	-0.053092
Au	4.271365	1.821228	1.705499
Au	1.650677	-2.224533	1.295696
Au	4.122179	-0.427597	-2.365941
Au	4.06433	-0.912444	2.42517
S	6.042935	-1.107099	-3.721003
Au	2.09796	5.742477	3.298642
Au	-0.468438	4.301248	3.757934
Au	4.303628	3.922331	3.634666
Au	0.23872	1.583238	4.628876
Au	3.280753	1.31261	4.384645
Au	1.978744	3.60872	5.477523
S	4.795863	6.096918	-0.053754
S	-0.514884	5.756415	-0.905213
S	5.786478	-1.924397	3.757429
S	-2.148677	-0.936631	0.121198
S	7.83717	1.183496	0.234657
S	6.713357	3.940795	3.656493
S	0.349963	-3.610555	2.907676
S	0.174196	2.254132	-3.749248
S	-1.094198	-0.174774	5.562605
S	2.846546	4.73557	7.440009
S	4.402507	-4.701021	-0.009915
Au	-1.035105	-3.072065	-0.380282
C	-2.476806	-1.170115	1.902283
Au	2.171719	-4.716032	-0.3655
C	4.9683	-5.152153	-1.715455
Au	4.887386	6.803943	2.335134
C	6.346402	5.116065	-0.210323
Au	7.215229	2.429186	1.917304

C	8.354107	1.969733	-1.364192
C	7.448606	3.297908	5.216111
Au	-0.13801	3.93663	-2.178931
C	0.389174	7.281417	-1.44014
C	-1.511247	1.634972	-4.149845
Au	-0.256939	-1.747574	4.037992
C	-2.930897	-0.082393	5.509372
C	1.522144	-4.826193	3.692569
Au	3.953537	6.111819	5.809143
C	4.445827	3.73349	7.599813
S	5.103888	7.58255	4.490278
C	4.646552	9.352287	4.623354
S	-0.100991	-5.164155	-0.838266
C	-0.680723	-6.244361	0.552097
S	-2.995339	2.321797	0.999896
S	1.577526	8.151526	3.280395
Au	-2.746853	4.732647	1.722321
C	-2.71865	2.421415	-0.816732
Au	-0.585695	7.364775	2.679448
C	1.033652	8.807903	4.946061
S	-2.848941	6.968747	2.278998
C	-3.284136	7.755764	0.658856
S	0.973978	-2.761629	-3.727039
S	4.170845	4.068733	-2.767007
Au	2.129095	-1.024841	-4.625212
C	-0.670269	-1.890513	-3.865353
Au	3.66873	2.171128	-4.310375
C	2.832778	5.315467	-2.871804
S	3.280735	0.558463	-5.952124
C	1.988159	1.503229	-6.927644
S	-2.236481	4.726764	5.328952
S	3.81999	-0.138523	6.326188
Au	-0.969167	3.415224	6.74991

C	-3.402014	3.356832	4.831839
Au	1.819277	1.19165	7.306909
C	3.025065	-1.742124	5.862985
S	0.033642	2.15472	8.466645
C	0.375479	3.432845	9.757942
Au	6.921952	-2.143059	-1.86889
C	5.273398	-2.525584	-4.650596
S	7.908119	-3.345744	-0.128234
C	7.415032	5.849793	-1.043332
C	8.51362	6.361389	-0.140762
C	9.318523	5.46383	0.577124
C	8.835947	7.721125	-0.082708
C	10.114438	5.926644	1.624078
C	10.073859	7.280473	1.968348
C	9.611904	8.179535	0.995056
C	-3.926414	9.125303	0.876005
C	-3.821066	10.033258	-0.332281
C	-3.443692	11.373094	-0.158074
C	-3.804337	9.521055	-1.63702
C	-3.54915	12.264715	-1.227572
C	-4.107526	11.817192	-2.427038
C	-3.996572	10.437006	-2.687322
C	9.704183	-2.854661	-0.07366
C	10.007625	-1.605005	-0.920872
C	11.437423	-1.113379	-0.776923
C	12.476567	-1.928701	-0.301673
C	11.800208	0.132044	-1.316289
C	13.762379	-1.404153	-0.122513
C	14.032597	-0.071195	-0.436757
C	13.021776	0.720748	-0.99494
C	-4.020724	2.057463	-1.529406
C	-5.08714	3.125909	-1.40328
C	-4.757535	4.482969	-1.542083

C	-6.321663	2.822744	-0.818728
C	-7.385131	3.720434	-0.934828
C	-7.16053	4.935593	-1.586408
C	-5.826263	5.390362	-1.636966
C	7.26316	2.123422	-2.416021
C	7.791729	2.340616	-3.811315
C	7.35678	3.430938	-4.577918
C	8.700472	1.441444	-4.394708
C	7.716695	3.524656	-5.925267
C	9.16099	1.629933	-5.700068
C	8.778809	2.77026	-6.422983
C	1.572783	7.702735	-0.560711
C	1.710758	9.200161	-0.493478
C	0.682406	10.027936	-0.012351
C	2.912428	9.851889	-0.840553
C	0.686966	11.369504	-0.413475
C	1.926451	12.027258	-0.374474
C	3.059807	11.231559	-0.62104
C	4.882219	10.172967	3.368799
C	4.110847	11.47812	3.322183
C	3.136893	11.85514	4.257037
C	4.350751	12.359201	2.254145
C	2.453215	13.069201	4.120296
C	2.935816	14.035396	3.231759
C	3.96226	13.695042	2.338279
C	-2.232922	2.679405	-4.995665
C	-3.518924	2.234405	-5.657493
C	-3.896644	0.892308	-5.785372
C	-4.491526	3.200386	-5.979957
C	-4.973004	0.549741	-6.612833
C	-5.963771	1.495765	-6.877416
C	-5.692111	2.84089	-6.594133
C	-1.353958	-2.167567	-5.218887

C	-2.592886	-3.011783	-5.036895
C	-2.63369	-4.332554	-5.501864
C	-3.748414	-2.48657	-4.436989
C	-3.6999	-5.171421	-5.162849
C	-4.781973	-3.332531	-4.033064
C	-4.761912	-4.681632	-4.399591
C	2.903882	-4.778505	3.059394
C	3.921498	-5.765505	3.56109
C	3.641821	-7.135346	3.68518
C	5.235078	-5.34689	3.828711
C	4.635612	-8.017924	4.120584
C	5.968675	-7.58229	4.174066
C	6.285268	-6.259884	3.811897
C	1.012755	7.787788	6.087341
C	-0.238609	7.714312	6.936353
C	-0.142192	7.145569	8.216936
C	-1.48959	8.18609	6.525646
C	-1.287953	6.749834	8.902294
C	-2.538634	6.918938	8.300685
C	-2.616985	7.933578	7.320849
C	6.78374	-0.382991	4.077198
C	3.415967	6.612704	-3.46136
C	3.072211	6.760284	-4.925544
C	1.73566	6.810617	-5.349976
C	4.070325	6.984902	-5.883676
C	1.453522	6.854748	-6.716488
C	3.759703	6.758306	-7.236529
C	2.437258	6.478667	-7.631817
C	7.856639	-0.640423	5.13813
C	9.220938	-0.972368	4.573314
C	10.248645	-1.40778	5.429524
C	9.621991	-0.594611	3.284002
C	11.244512	-2.236362	4.909444

C	11.440979	-2.211801	3.518429
C	10.730656	-1.282947	2.749167
C	5.9991	-2.842264	-5.974679
C	4.977195	-3.12986	-7.047038
C	4.549981	-2.131936	-7.935335
C	4.259439	-4.342013	-7.041455
C	3.673914	-2.479154	-8.969907
C	2.784346	-3.538371	-8.797136
C	3.186655	-4.554255	-7.913797
C	-4.778102	3.460079	5.514088
C	-5.895717	3.259335	4.522654
C	-6.910352	4.204647	4.332768
C	-6.015007	2.052244	3.812569
C	-7.980122	3.920372	3.47374
C	-8.006148	2.720496	2.750271
C	-6.821855	1.944114	2.688139
C	-3.551897	-1.454162	5.803041
C	-4.341087	-1.598094	7.089566
C	-4.94164	-2.840226	7.366347
C	-4.499715	-0.586926	8.050181
C	-5.46718	-3.072073	8.637994
C	-5.997887	-2.001448	9.360052
C	-5.376223	-0.763502	9.134282
C	8.954073	3.512188	5.252081
C	9.422165	4.927225	5.529256
C	8.638454	5.874678	6.198381
C	10.752717	5.261565	5.233711
C	9.110847	7.172741	6.409176
C	11.233398	6.552327	5.456876
C	10.379038	7.540525	5.958937
C	0.696533	0.735963	-7.220483
C	0.575822	0.113183	-8.595455
C	1.093682	0.726184	-9.745974

C	-0.071757	-1.12061	-8.750071
C	-0.129279	-1.747872	-9.995619
C	0.788949	0.229784	-11.016093
C	0.180927	-1.0227	-11.148781
C	3.21004	-2.759004	6.988434
C	2.23254	-2.468394	8.107343
C	2.645029	-2.344785	9.43827
C	0.851812	-2.59736	7.878753
C	-0.036006	-2.10983	8.84107
C	1.800354	-1.598616	10.283955
C	0.468214	-1.356597	9.908134
C	4.990027	3.647896	9.034298
C	5.555839	2.264362	9.282141
C	5.279439	1.557936	10.459944
C	6.613824	1.770787	8.50492
C	5.32959	0.165973	10.44959
C	6.258159	-0.378799	9.538114
C	7.100329	0.491255	8.808414
C	1.595013	3.142213	10.616898
C	1.500189	3.680343	12.033847
C	2.623438	3.587929	12.876211
C	0.541986	4.628425	12.416294
C	2.435118	3.646795	14.256843
C	0.41243	4.884884	13.795949
C	1.245783	4.237881	14.719008
C	0.35462	-7.287643	0.955719
C	0.009912	-8.063517	2.20977
C	-0.5985	-7.464337	3.323166
C	0.534852	-9.354941	2.381331
C	-0.829529	-8.19865	4.49051
C	0.113384	-10.157552	3.44245
C	-0.543136	-9.565505	4.527321
C	-3.948984	-1.465356	2.168509

C	-4.247962	-2.943979	2.105742
C	-5.376039	-3.440511	1.445796
C	-3.46868	-3.830176	2.870415
C	-5.583554	-4.821097	1.379644
C	-4.549486	-5.691651	1.716507
C	-3.645302	-5.209042	2.687255
C	5.055249	-6.626682	-2.051056
C	6.384997	-6.985733	-2.713813
C	7.478482	-6.104923	-2.829382
C	6.636427	-8.333066	-3.028838
C	8.718389	-6.607954	-3.244949
C	8.7393	-7.774486	-4.021529
C	7.635237	-8.644765	-3.953788
H	-8.389428	3.429611	-0.621152
H	15.017346	0.352499	-0.233083
H	-3.734043	4.81505	-1.723881
H	14.553257	-2.040769	0.278641
H	-0.266143	9.570619	0.295175
H	-3.356534	13.32793	-1.067995
H	11.039024	0.738902	-1.811647
H	10.580087	7.639337	2.864997
H	13.2275	1.750323	-1.293103
H	-3.177121	11.735265	0.837192
H	-4.332448	12.512279	-3.23729
H	-5.597636	0.088441	9.780783
H	12.274098	-2.950033	0.024351
H	0.476732	-2.958082	6.918156
H	-0.19892	-0.784784	10.553846
H	10.73617	8.56354	6.088748
H	4.63662	2.045166	11.199081
H	-0.562085	-2.747255	-10.078113
H	3.5326	3.119583	12.489221
H	10.642427	5.214524	2.260492

H	-0.20791	5.007828	11.722039
H	1.632859	13.310579	4.798819
H	-1.110486	-2.128246	8.644813
H	6.463846	-1.451573	9.537447
H	4.814117	-0.446236	11.187944
H	9.215922	4.387788	0.417671
H	2.828068	11.152382	5.043225
H	-0.234638	11.951494	-0.312934
H	3.21625	3.331085	14.949408
H	-0.401242	5.519076	14.153321
H	1.051224	4.330922	15.788792
H	2.510848	15.041759	3.228171
H	-6.494601	1.816181	-0.433014
H	-7.981685	5.628529	-1.777987
H	3.456825	-1.732657	-9.739847
H	-5.626487	6.434296	-1.893084
H	8.361395	8.430257	-0.763722
H	1.95987	-3.693781	-9.493335
H	4.318619	14.421082	1.603835
H	9.752351	9.25344	1.137246
H	-4.163862	10.064304	-3.700312
H	-3.833791	8.448101	-1.831389
H	5.081475	-1.17843	-7.969005
H	5.068296	12.058996	1.485784
H	10.042364	-1.443653	6.50522
H	2.000057	13.117231	-0.36354
H	6.604203	4.105912	-4.165051
H	11.906606	-2.802027	5.566416
H	-3.208228	0.104735	-5.481467
H	9.004281	0.546102	-3.846892
H	-6.86979	5.161386	4.858972
H	3.768057	9.258627	-1.173391
H	2.60895	-5.478116	-7.837748

H	-8.825331	4.608469	3.413669
H	2.632758	-7.513839	3.502367
H	4.564569	-5.141329	-6.360383
H	4.044034	11.694817	-0.729688
H	-5.151109	-0.504311	-6.838801
H	0.845937	6.927058	8.629709
H	12.200533	-2.837957	3.047106
H	0.921825	6.852129	-4.623059
H	7.303397	4.338942	-6.526309
H	-1.77725	-4.735294	-6.047877
H	4.389441	-9.059695	4.332994
H	9.891666	0.934937	-6.119255
H	-4.764345	-3.680322	6.689138
H	-8.823556	2.536991	2.049086
H	9.149484	2.931608	-7.436596
H	-3.772114	-1.439442	-4.128002
H	5.106692	7.131742	-5.573817
H	-1.604576	8.771427	5.612838
H	1.624771	1.677227	-9.668072
H	5.477729	-4.285137	3.680343
H	8.999162	0.023479	2.626643
H	-4.279767	4.255471	-5.787913
H	-5.223283	1.307444	3.952231
H	-6.878336	1.213315	-7.401073
H	3.696464	-2.482892	9.699863
H	10.944267	-1.221282	1.679015
H	-1.196262	6.184551	9.831903
H	-6.757393	1.06919	2.039203
H	7.61665	5.640846	6.501538
H	-3.695068	-6.213322	-5.488737
H	0.408228	6.903273	-7.034363
H	1.093116	0.787851	-11.903392
H	6.763756	-8.302828	4.378051

H	-5.782973	-4.08969	8.888467
H	-3.996965	0.375219	7.943544
H	11.407688	4.507565	4.78967
H	7.075075	2.377087	7.725931
H	-6.436597	3.60897	-6.811953
H	-5.627754	-2.927342	-3.47468
H	2.175601	-1.223762	11.238917
H	7.320536	-5.914888	3.791544
H	4.566474	6.725648	-7.972668
H	-3.5943	8.285149	6.982024
H	-3.444081	6.555631	8.790198
H	2.198093	6.292458	-8.679967
H	-5.574011	-5.345413	-4.097695
H	8.456474	7.912039	6.874365
H	-0.42911	-1.64864	-7.864997
H	7.946426	0.079873	8.254988
H	0.014852	-1.453765	-12.13733
H	12.259875	6.803501	5.181551
H	-6.634655	-2.158487	10.231574
H	-0.928928	-6.426271	3.272514
H	1.109318	-9.802212	1.565548
H	-1.29522	-7.714149	5.350989
H	0.398387	-11.21051	3.48009
H	-0.814242	-10.160117	5.401146
H	-6.447196	-5.196245	0.823765
H	-2.63824	-3.455043	3.48645
H	-6.093891	-2.75123	0.996759
H	-2.973101	-5.908584	3.186193
H	-4.626389	-6.762083	1.518658
H	7.3991	-5.042127	-2.551612
H	5.852189	-9.069765	-2.825988
H	9.596285	-5.958373	-3.221163
H	9.634155	-8.053962	-4.579998

H	7.666863	-9.62197	-4.438214
Au	6.825468	-2.561877	1.697851
H	-4.983957	8.993091	1.156928
H	6.96483	6.671619	-1.617066
H	6.718551	4.875306	0.790278
H	9.780969	-1.805489	-1.981786
H	-1.924657	1.697713	-1.046533
H	9.332069	-0.794979	-0.602341
H	9.12678	1.277134	-1.718783
H	2.509011	7.24608	-0.898404
H	9.97353	-2.562201	0.954022
H	-3.442402	9.62413	1.731345
H	7.826116	5.13639	-1.777717
H	8.842354	2.925082	-1.131904
H	4.584977	9.570503	2.488936
H	-2.366333	7.823966	0.063738
H	6.64329	1.211618	-2.407095
H	6.036922	4.178374	-0.685279
H	10.243687	-3.671134	-0.568093
H	-2.35622	3.420104	-1.079561
H	6.586965	2.943051	-2.143177
H	-3.813741	1.910009	-2.603229
H	1.395873	7.313981	0.459147
H	0.685447	7.146773	-2.487288
H	-3.962088	7.054181	0.157983
H	5.957997	10.36862	3.254198
H	-4.3908	1.093729	-1.148413
H	-1.279274	-2.286481	-3.038523
H	2.480983	5.451172	-1.843274
H	5.209723	-3.397634	-3.993186
H	3.640257	9.405909	5.045343
H	-3.532869	3.488692	3.74699
H	-1.339663	0.721788	-4.733497

H	2.004983	4.924393	-3.473276
H	-0.391294	8.054163	-1.415283
H	3.308608	-3.758368	3.150847
H	-3.189904	0.675139	6.256681
H	-1.53547	3.026369	-5.778838
H	-0.500254	-0.829864	-3.664153
H	7.250554	-0.077412	3.141054
H	-2.899217	2.403098	4.988283
H	1.882634	7.937232	6.74452
H	7.540333	-1.454565	5.808378
H	-1.607267	-1.211824	-5.704323
H	1.153444	6.784407	5.64814
H	5.300314	9.710405	5.434993
H	-3.239901	0.294132	4.525852
H	-2.049283	1.362991	-3.237038
H	4.247031	-2.197595	-4.868598
H	7.193335	2.236239	5.307265
H	2.771565	-4.950577	1.979048
H	1.738273	9.635949	5.155186
H	2.987937	7.461123	-2.902009
H	-0.650477	-2.680631	-5.890012
H	4.501804	6.651759	-3.304975
H	0.060092	9.274754	4.776773
H	-4.848996	2.715081	6.324399
H	1.564548	-4.621078	4.771276
H	-2.429574	3.566417	-4.370864
H	-2.761659	-2.221117	5.783423
H	6.609448	-1.977461	-6.273974
H	9.363256	2.853241	6.041134
H	6.066463	0.365694	4.433048
H	6.682496	-3.693903	-5.839044
H	7.929724	0.25329	5.783292
H	6.939839	3.829504	6.027917

H	1.042714	-5.800528	3.553127
H	-4.230082	-1.727519	4.979103
H	1.763351	2.397951	-6.339594
H	-4.886822	4.446872	5.98787
H	1.965371	-1.573681	5.63166
H	3.524506	-2.068277	4.944668
H	9.394555	3.153387	4.308536
H	-0.138367	1.450482	-7.10294
H	2.51444	1.813205	-7.838163
H	5.178054	4.151302	6.905783
H	3.029071	-3.763469	6.565735
H	4.180116	3.839293	9.752317
H	0.545528	-0.035623	-6.449255
H	4.247157	-2.740534	7.352583
H	4.186823	2.727363	7.245281
H	-0.551318	3.403614	10.347453
H	1.745786	2.050581	10.679913
H	5.747742	4.430618	9.198111
H	2.49403	3.530769	10.114447
H	0.449427	4.410337	9.267931
H	0.527339	-7.989358	0.125538
H	1.319767	-6.767224	1.109404
H	-1.614966	-6.702504	0.19948
H	-0.917922	-5.573777	1.386166
H	-4.198002	-1.083671	3.170522
H	-2.175996	-0.224125	2.375508
H	-1.829896	-1.965102	2.280519
H	-4.579467	-0.905594	1.461512
H	4.960424	-7.236557	-1.137323
H	5.944304	-4.664838	-1.766583
H	4.219279	-6.918386	-2.70402
H	4.319119	-4.614314	-2.41577
**B2-A**			

Au	0.006595	0.000841	-0.033329
Au	2.811339	0.01477	0.001934
Au	1.31981	2.47026	-0.004018
Au	1.549694	-1.855345	-1.615233
Au	1.426877	0.266126	2.423279
Au	0.292225	4.814947	1.090135
Au	3.177217	4.514194	0.8369
Au	3.898451	2.353355	-1.168992
Au	-0.772874	2.087443	1.880416
Au	1.505	0.96428	-2.341773
Au	1.754107	3.025882	2.71915
Au	5.648492	0.059426	-0.051131
Au	4.229843	-2.340634	-0.154385
Au	4.234107	1.808361	1.634755
Au	1.612865	-2.217635	1.207614
Au	4.064491	-0.409704	-2.452119
Au	4.033646	-0.918681	2.336939
S	5.951423	-1.041866	-3.861831
Au	2.06171	5.725892	3.265025
Au	-0.511475	4.28584	3.725994
Au	4.27034	3.916976	3.580863
Au	0.204619	1.552601	4.57318
Au	3.246352	1.288715	4.314498
Au	1.953382	3.586234	5.430694
S	4.904075	6.015105	-0.093137
S	-0.757156	5.841089	-0.810818
S	5.738808	-1.96211	3.694152
S	-2.284388	-0.992719	0.099603
S	7.849972	0.999125	0.220313
S	6.66942	4.160844	3.450957
S	0.383401	-3.576917	2.831422
S	0.155296	2.355884	-3.798461
S	-1.259862	-0.108375	5.460927

S	2.996796	4.573688	7.385408
S	4.518501	-4.738286	-0.214081
Au	-1.093575	-3.094698	-0.456354
C	-2.656063	-0.963668	1.890203
Au	2.078732	-4.709204	-0.466849
C	4.922662	-5.135142	-1.975376
Au	4.843302	6.73166	2.294936
C	6.503586	5.14471	-0.36836
Au	7.149946	2.48768	1.857753
C	7.956294	1.773642	-1.462154
C	7.490129	3.641659	5.02182
Au	-0.233913	4.025283	-2.226229
C	0.257744	7.28254	-1.414996
C	-1.483944	1.766487	-4.350682
Au	-0.30625	-1.758307	4.001866
C	-3.093307	-0.067019	5.386548
C	1.623747	-4.480176	3.885001
Au	3.941123	6.097377	5.741863
C	4.590075	3.592486	7.394261
S	5.031954	7.692148	4.430669
C	6.814708	7.445566	4.842965
S	-0.170822	-5.202984	-0.905884
C	-0.545287	-6.168197	0.64977
S	-3.021534	2.335965	0.92378
S	1.56411	8.096409	3.281582
Au	-2.782462	4.737344	1.628351
C	-2.543615	2.268477	-0.842387
Au	-0.634668	7.315788	2.690267
C	1.125466	8.59471	5.018236
S	-2.895359	7.008498	2.123542
C	-3.800786	7.200961	3.731827
S	0.756329	-2.700075	-3.724837
S	4.076724	4.089943	-2.846918

Au	1.93364	-0.890246	-4.699834
C	-0.885809	-1.875683	-3.487728
Au	3.594657	2.204232	-4.364392
C	2.757443	5.315377	-2.697848
S	2.991082	0.727603	-6.081267
C	4.664776	0.277353	-6.701475
S	-2.30346	4.60427	5.394816
S	3.790278	-0.179179	6.182067
Au	-0.955928	3.359056	6.741581
C	-3.512449	3.4148	4.660271
Au	1.865923	1.123296	7.253935
C	2.800082	-1.707972	5.856037
S	0.094491	2.074203	8.389174
C	0.633802	3.210077	9.735035
Au	6.774279	-2.094778	-1.886354
C	5.571946	-2.563616	-4.850296
S	7.854567	-3.310718	-0.174483
C	7.642733	6.141199	-0.646756
C	8.626388	6.410814	0.474135
C	9.17028	7.69882	0.617459
C	9.35836	5.354164	1.038167
C	10.144121	7.876166	1.604128
C	10.145042	7.028482	2.705057
C	10.03069	5.678556	2.246098
C	-4.31023	8.635863	3.873845
C	-5.703381	8.896809	3.341711
C	-6.030813	10.159843	2.825243
C	-6.741538	7.959198	3.453063
C	-7.255947	10.365881	2.187645
C	-8.325115	9.514833	2.487435
C	-8.059744	8.311284	3.149456
C	9.602035	-2.680994	-0.151433
C	9.937978	-1.81795	-1.375602

C	11.261001	-2.06619	-2.063467
C	12.064719	-3.198946	-1.866143
C	11.742987	-1.095917	-2.96698
C	13.138205	-3.430368	-2.737193
C	13.794457	-2.355759	-3.331255
C	12.998472	-1.220249	-3.569424
C	-3.815038	1.947542	-1.63258
C	-4.717506	3.160148	-1.630717
C	-4.216031	4.401787	-2.052451
C	-6.067251	3.100252	-1.282512
C	-6.752007	4.264527	-0.927589
C	-6.101174	5.48794	-1.151349
C	-5.085632	5.48038	-2.182347
C	9.349146	2.304165	-1.794112
C	9.749057	2.197796	-3.258512
C	9.225753	1.238927	-4.140875
C	10.948663	2.805713	-3.669679
C	9.239197	1.500182	-5.512441
C	11.355025	2.61318	-5.0004
C	10.367011	2.235807	-5.943881
C	1.532911	7.582998	-0.630425
C	1.928738	9.043711	-0.568025
C	0.990742	10.080343	-0.454126
C	3.285251	9.377438	-0.424148
C	1.412673	11.410472	-0.363736
C	2.769616	11.723817	-0.259274
C	3.713125	10.704025	-0.439015
C	7.592482	8.704548	4.363718
C	8.531001	9.310846	5.386188
C	9.700854	9.956783	4.952165
C	8.274955	9.312624	6.767122
C	10.631783	10.426835	5.88055
C	10.204591	10.726962	7.177191

C	9.024277	10.124104	7.62815
C	-2.17356	2.832344	-5.205431
C	-1.919693	2.742792	-6.695742
C	-1.694244	1.53171	-7.363858
C	-2.035466	3.902625	-7.480588
C	-1.409176	1.570625	-8.73166
C	-0.864787	2.71795	-9.292742
C	-1.505067	3.883418	-8.786057
C	-1.745002	-1.711724	-4.721507
C	-3.197452	-1.904823	-4.30727
C	-4.135602	-0.892221	-4.567988
C	-3.58226	-2.909857	-3.389835
C	-5.383688	-0.896609	-3.940904
C	-4.892351	-2.958731	-2.899702
C	-5.875683	-2.072653	-3.383917
C	2.986789	-4.617092	3.202087
C	3.723067	-5.895388	3.509871
C	3.182556	-7.146644	3.168136
C	5.085713	-5.869165	3.85096
C	3.893185	-8.309205	3.489177
C	5.2406	-8.258199	3.85837
C	5.76678	-7.016437	4.256943
C	0.614623	7.424415	5.869605
C	-0.399132	7.732608	6.937239
C	-1.550522	8.493824	6.677034
C	-0.34434	7.050082	8.16746
C	-2.432404	8.761122	7.730026
C	-2.523575	7.894742	8.81844
C	-1.348669	7.18625	9.131323
C	6.788516	-0.460532	3.992563
C	3.097281	6.510835	-3.582174
C	2.914559	6.102238	-5.029437
C	1.713481	5.506201	-5.458022

C	3.896557	6.336448	-6.005152
C	1.685233	4.87493	-6.705287
C	3.79816	5.801184	-7.294878
C	2.58149	5.234918	-7.712444
C	7.984542	-0.845302	4.899442
C	9.176892	-1.419384	4.178735
C	9.670093	-2.697604	4.463478
C	9.874503	-0.6294	3.247627
C	10.800599	-3.191593	3.804898
C	11.446753	-2.416581	2.840715
C	11.060648	-1.079714	2.670105
C	6.755868	-2.924235	-5.766347
C	7.586091	-4.039039	-5.174281
C	8.869413	-3.792035	-4.670858
C	7.017782	-5.306542	-4.970698
C	9.624791	-4.833679	-4.125492
C	9.175318	-6.148891	-4.237552
C	7.800635	-6.3459	-4.458856
C	-4.830859	3.306127	5.433678
C	-5.949205	2.920187	4.494254
C	-6.390077	1.594298	4.401901
C	-6.475096	3.848831	3.583014
C	-7.427767	1.274291	3.521594
C	-8.199126	2.279171	2.944867
C	-7.458927	3.465684	2.666687
C	-3.651642	-1.415194	5.863783
C	-4.114934	-1.475582	7.299668
C	-3.810972	-2.598536	8.085709
C	-4.71383	-0.38943	7.959093
C	-4.381462	-2.728474	9.357819
C	-5.017969	-1.650609	9.976833
C	-5.357883	-0.540086	9.186674
C	8.892281	3.084564	4.773062

C	10.003791	3.50643	5.702361
C	11.12921	2.672275	5.852281
C	9.905979	4.53244	6.660944
C	12.314307	3.272871	6.306847
C	11.063002	5.161627	7.135091
C	12.278171	4.469589	7.035096
C	5.581415	1.50746	-6.678237
C	6.562529	1.621144	-7.820673
C	7.018502	0.511239	-8.550179
C	6.976148	2.888954	-8.259267
C	7.922889	3.002438	-9.282059
C	8.121177	0.600788	-9.399723
C	8.410588	1.866827	-9.938811
C	3.183609	-2.784541	6.870829
C	2.560257	-2.462601	8.213599
C	1.164105	-2.360573	8.316709
C	3.31686	-2.346441	9.382394
C	2.765424	-1.746337	10.517775
C	0.593226	-1.976258	9.532894
C	1.425723	-1.348101	10.490584
C	5.560161	4.102015	8.458861
C	5.378791	3.435236	9.804717
C	5.322127	2.036917	9.919744
C	5.376315	4.19188	10.98495
C	5.06227	1.43962	11.15439
C	5.017471	2.209921	12.319405
C	5.103489	3.601243	12.221427
C	1.441354	2.461771	10.799751
C	0.788272	2.22294	12.146002
C	1.435268	1.348183	13.041085
C	-0.562643	2.472606	12.415131
C	0.964669	1.254823	14.353807
C	-0.920478	2.622263	13.76857

C	-0.094483	2.078585	14.762488
C	-1.126129	-7.528676	0.298196
C	-0.137784	-8.536859	-0.262065
C	-0.620649	-9.680208	-0.915683
C	1.237979	-8.464592	-0.006822
C	0.246696	-10.545499	-1.581773
C	2.095078	-9.474204	-0.454065
C	1.625356	-10.423155	-1.368952
C	-4.131136	-1.238981	2.127727
C	-4.463346	-2.686519	1.835485
C	-3.694162	-3.686873	2.455999
C	-5.669377	-3.054116	1.237831
C	-3.781489	-5.001276	2.003463
C	-4.797202	-5.315402	1.087951
C	-5.882257	-4.418939	0.980846
C	4.458875	-6.534179	-2.392597
C	3.518902	-6.611865	-3.583738
C	2.868313	-7.833514	-3.829461
C	3.064759	-5.500532	-4.305887
C	2.219964	-8.053897	-5.043516
C	1.914613	-6.96001	-5.864452
C	2.269453	-5.677019	-5.445448
H	-7.702649	4.214597	-0.396617
H	14.758118	-2.481483	-3.826831
H	-3.202247	4.454365	-2.473586
H	13.6574	-4.391486	-2.664685
H	-0.079694	9.871127	-0.495154
H	-7.434057	11.296417	1.644314
H	11.139748	-0.210354	-3.178278
H	10.655215	7.261865	3.640013
H	13.377458	-0.393686	-4.174356
H	-5.240627	10.908816	2.723817
H	-9.333933	9.744681	2.140831

H	-5.918897	0.293913	9.612403
H	11.723157	-4.019199	-1.232282
H	4.38334	-2.583554	9.35429
H	0.981577	-0.836468	11.347087
H	13.203281	4.908968	7.412425
H	5.365637	1.40853	9.027146
H	8.213281	3.994861	-9.63423
H	2.372795	0.870736	12.750785
H	10.581422	8.877431	1.703626
H	-1.221633	2.883862	11.648469
H	11.596331	10.800644	5.527861
H	3.401867	-1.495944	11.36892
H	4.830813	1.739061	13.286074
H	4.969347	0.353609	11.210798
H	8.888608	8.50559	-0.05957
H	9.964061	9.916286	3.892535
H	0.665862	12.203061	-0.279819
H	1.49972	0.645157	15.084885
H	-1.874726	3.079674	14.036085
H	-0.371005	2.151451	15.815264
H	10.844272	11.280892	7.865906
H	-6.5217	2.124443	-1.089913
H	-6.600722	6.425693	-0.893883
H	10.635643	-4.62408	-3.771331
H	-4.734195	6.425697	-2.602641
H	9.208041	4.315167	0.746064
H	9.804944	-6.980953	-3.917554
H	8.707645	10.244649	8.665938
H	10.399019	4.87178	2.883783
H	-8.867851	7.607557	3.357657
H	-6.553584	6.976827	3.889188
H	9.278925	-2.779622	-4.697897
H	7.388504	8.816499	7.168971

H	9.160791	-3.313638	5.207908
H	3.087036	12.765057	-0.17668
H	8.450199	0.555157	-3.775493
H	11.14269	-4.208432	4.006843
H	-1.835978	0.569221	-6.873439
H	11.564471	3.371464	-2.964848
H	-5.905485	0.811152	4.987884
H	4.027679	8.576429	-0.465646
H	7.371916	-7.347121	-4.374142
H	-7.799074	0.244569	3.51689
H	2.148939	-7.229984	2.822511
H	5.972752	-5.491994	-5.232094
H	4.781243	10.929591	-0.41085
H	-1.25638	0.610243	-9.236669
H	-1.640737	9.053569	5.742783
H	12.323554	-2.803945	2.318723
H	0.924679	5.272443	-4.730959
H	8.570758	1.008502	-6.219382
H	-3.829971	-0.018697	-5.149265
H	3.440986	-9.278261	3.261455
H	12.304294	3.028344	-5.351045
H	-3.272717	-3.440147	7.64269
H	-9.093843	2.035446	2.36869
H	10.557953	2.369643	-7.012474
H	-2.83261	-3.49893	-2.834709
H	4.835009	6.806119	-5.696729
H	0.540785	6.449762	8.395044
H	6.686709	-0.485395	-8.241703
H	5.546971	-4.892266	4.029116
H	9.527597	0.385414	3.034267
H	-2.396531	4.836683	-7.04751
H	-6.019509	4.837549	3.497428
H	-0.392234	2.718135	-10.275545

H	0.523926	-2.617974	7.469349
H	11.605507	-0.436087	1.976373
H	-3.259472	9.45402	7.542518
H	-7.851556	4.209681	1.970301
H	11.179948	1.725796	5.304536
H	-6.062054	-0.057934	-4.122721
H	0.7981	4.293788	-6.970445
H	8.550971	-0.301615	-9.838902
H	5.788696	-9.18306	4.04961
H	-4.174765	-3.631198	9.938402
H	-4.909209	0.527339	7.394318
H	8.965777	5.083763	6.756341
H	5.46196	5.279377	10.915676
H	-1.423299	4.828138	-9.329829
H	-5.166934	-3.753691	-2.202368
H	-0.492085	-1.881027	9.612246
H	6.790543	-6.938603	4.628371
H	4.59886	5.984835	-8.015245
H	-1.282497	6.608769	10.056302
H	-3.330602	7.990878	9.546363
H	2.46737	4.835955	-8.722096
H	-6.904419	-2.139018	-3.025064
H	13.248004	2.707214	6.25892
H	6.608198	3.783165	-7.750406
H	5.043093	4.219695	13.118628
H	9.1573	1.97449	-10.727995
H	10.985757	6.075633	7.726547
H	-5.416181	-1.757079	10.987527
H	-1.699961	-9.798097	-1.046526
H	1.659466	-7.637847	0.566294
H	-0.148006	-11.342216	-2.21416
H	3.158712	-9.422738	-0.211534
H	2.317185	-11.128897	-1.832864

H	-3.066205	-5.756332	2.331303
H	-6.406129	-2.301302	0.952019
H	-2.83448	-3.394303	3.081876
H	-6.792033	-4.737781	0.466775
H	-4.896536	-6.331587	0.700637
H	3.053365	-8.675723	-3.15845
H	3.374685	-4.488493	-4.045554
H	1.852709	-9.050769	-5.294249
H	1.360236	-7.102985	-6.793725
H	1.946498	-4.802362	-6.012689
Au	6.709387	-2.60607	1.586963
H	-4.308419	8.871631	4.954177
H	7.215066	7.091548	-1.000959
H	6.721514	4.481374	0.480966
H	9.124658	-1.958998	-2.12363
H	-1.782516	1.48799	-0.956373
H	9.864484	-0.758636	-1.087903
H	7.168414	2.528831	-1.568403
H	2.3776	6.996353	-0.99441
H	9.720449	-2.078372	0.75116
H	-3.595845	9.333571	3.411517
H	8.218235	5.740245	-1.500395
H	7.686193	0.935023	-2.114222
H	6.862821	9.48673	4.087712
H	-4.616848	6.471114	3.714735
H	9.447117	3.355252	-1.480895
H	6.298459	4.520952	-1.248137
H	10.211126	-3.589154	-0.11556
H	-2.105304	3.226944	-1.140165
H	10.088163	1.747477	-1.187072
H	-3.561816	1.666393	-2.664555
H	1.371469	7.239365	0.408438
H	0.449626	7.117066	-2.481386

H	-3.120649	6.924291	4.54276
H	8.144885	8.47378	3.443862
H	-4.317498	1.075464	-1.190036
H	-1.365227	-2.508969	-2.73481
H	2.736181	5.561811	-1.632882
H	5.340883	-3.383301	-4.166893
H	6.895405	7.474131	5.932017
H	-3.713619	3.834128	3.658813
H	-1.251073	0.880791	-4.944234
H	1.786423	4.869106	-2.950859
H	-0.450731	8.115995	-1.335317
H	3.611739	-3.744554	3.435588
H	-3.366072	0.754528	6.059436
H	-1.899509	3.833586	-4.840523
H	-0.711862	-0.906465	-3.001405
H	7.21189	-0.084825	3.057748
H	-3.021207	2.449042	4.504908
H	1.476948	6.9031	6.311104
H	7.645718	-1.533111	5.688831
H	-1.597571	-0.747243	-5.209368
H	0.149569	6.686308	5.191623
H	7.224534	6.58344	4.309017
H	-3.423137	0.192949	4.37481
H	-2.075198	1.451231	-3.490046
H	4.657196	-2.338049	-5.418557
H	6.856025	2.906232	5.532464
H	2.819637	-4.580163	2.110614
H	2.040058	9.027582	5.448568
H	2.43438	7.353152	-3.323908
H	-1.469029	-2.480165	-5.465889
H	4.129286	6.840708	-3.395368
H	0.381007	9.39253	4.913363
H	-4.741776	2.587915	6.261552

H	1.690544	-3.974142	4.851326
H	-3.262606	2.748039	-5.027712
H	-2.916503	-2.210707	5.674162
H	6.348929	-3.25061	-6.737645
H	8.848483	1.986726	4.720825
H	6.188846	0.222511	4.605342
H	7.378187	-2.038387	-5.960517
H	8.304883	0.076718	5.415424
H	7.498261	4.557061	5.626182
H	1.168362	-5.466539	4.052319
H	-4.520762	-1.666205	5.22692
H	5.075695	-0.53022	-6.094189
H	-5.054017	4.2843	5.889946
H	1.731284	-1.467471	5.885711
H	3.053019	-2.012191	4.830932
H	9.205321	3.396802	3.761954
H	6.127005	1.51126	-5.718155
H	4.502444	-0.09355	-7.7233
H	5.003781	3.648779	6.38335
H	2.81989	-3.761259	6.508649
H	5.48073	5.194412	8.557072
H	4.968379	2.421355	-6.67607
H	4.277929	-2.857915	6.946773
H	4.299642	2.548685	7.578984
H	1.232869	4.007539	9.279977
H	1.791323	1.500686	10.388038
H	6.584579	3.894805	8.098433
H	2.367784	3.030439	10.989748
H	-0.294634	3.654975	10.112167
H	-1.566911	-7.950813	1.220894
H	-1.96138	-7.399479	-0.407536
H	-1.263535	-5.572494	1.21919
H	0.371796	-6.234962	1.241927

H	-4.376298	-1.010145	3.17579
H	-2.37229	0.037354	2.239063
H	-2.010753	-1.684769	2.405975
H	-4.74158	-0.565075	1.507223
H	5.350432	-7.138033	-2.630671
H	6.013276	-5.028761	-2.052808
H	3.978357	-7.041361	-1.54087
H	4.473453	-4.344507	-2.578901
**B2-B**			
Au	-0.031375	0.036648	-0.073692
Au	2.77328	0.018733	-0.033507
Au	1.309977	2.491027	-0.058669
Au	1.493074	-1.847713	-1.640292
Au	1.387644	0.302265	2.38361
Au	0.307435	4.854654	1.017823
Au	3.189223	4.519217	0.771781
Au	3.889101	2.336765	-1.218215
Au	-0.790176	2.144914	1.824554
Au	1.481912	0.967265	-2.38589
Au	1.745946	3.060033	2.661466
Au	5.610844	0.030579	-0.081794
Au	4.164987	-2.353742	-0.171456
Au	4.213695	1.806925	1.589736
Au	1.547261	-2.19164	1.185002
Au	4.025688	-0.43662	-2.482399
Au	3.98082	-0.912839	2.309918
S	5.977894	-0.887307	-3.870202
Au	2.083509	5.759975	3.189688
Au	-0.50678	4.352598	3.655744
Au	4.270738	3.928089	3.521656
Au	0.176519	1.617133	4.522605
Au	3.215469	1.316731	4.271148
Au	1.946983	3.636378	5.369525

S	4.947997	6.051557	-0.075798
S	-0.733463	5.795144	-0.953707
S	5.759047	-1.873733	3.6193
S	-2.351575	-0.926117	-0.301594
S	7.720556	1.295357	-0.134396
S	6.7048	3.886364	3.65761
S	0.488557	-3.544321	2.831749
S	0.088119	2.249715	-3.909443
S	-1.34767	-0.12561	5.092999
S	2.994625	4.655475	7.289893
S	4.354642	-4.737842	-0.342899
Au	-1.120823	-3.064692	-0.494917
C	-2.85656	-0.868367	1.47819
Au	2.015186	-4.731531	-0.471569
C	4.765933	-5.001405	-2.121401
Au	4.834165	6.780764	2.262492
C	6.455658	5.067567	-0.406059
Au	7.086607	2.489271	1.801321
C	8.348493	2.110494	-1.677083
C	7.449212	2.898693	5.035876
Au	-0.166701	3.93613	-2.311826
C	0.203432	7.350123	-1.353208
C	-1.58731	1.550532	-4.220133
Au	-0.363471	-1.783399	3.864292
C	-1.375756	-0.644295	6.85944
C	1.674498	-4.601027	3.79124
Au	3.996179	6.150562	5.673364
C	4.529835	3.622619	7.491229
S	5.022868	7.793328	4.362825
C	6.791005	7.502563	4.854462
S	-0.258332	-5.189924	-0.671781
C	-0.767595	-5.820349	-2.335497
S	-3.085361	2.373217	1.129261

S	1.418541	8.079516	3.341692
Au	-2.825172	4.802215	1.611305
C	-2.77079	2.5172	-0.684756
Au	-0.610443	7.40293	2.63355
C	1.046772	8.48924	5.109723
S	-2.84802	7.101513	1.922891
C	-4.053208	7.382315	3.304653
S	0.754273	-2.720156	-3.786224
S	4.078951	4.140509	-2.793852
Au	2.105966	-1.056022	-4.736282
C	-0.859643	-1.812185	-3.748827
Au	3.590645	2.196443	-4.440364
C	2.766269	5.386725	-2.76273
S	3.387839	0.508165	-6.006594
C	2.257545	1.349468	-7.237314
S	-2.226624	4.835053	5.284056
S	3.683685	-0.14512	6.131557
Au	-0.962731	3.459366	6.735957
C	-3.505872	3.560997	4.853481
Au	1.836143	1.177954	7.21849
C	2.538046	-1.588956	5.899534
S	0.166326	2.226926	8.387275
C	0.896637	3.218171	9.749587
Au	6.78555	-2.166417	-1.915118
C	5.20433	-2.277974	-4.861612
S	7.716013	-3.469861	-0.163334
C	7.448408	5.924667	-1.186281
C	8.009729	7.049161	-0.34413
C	7.731748	8.388793	-0.650245
C	8.483408	6.804457	0.959114
C	8.310641	9.401302	0.132565
C	9.071388	9.088201	1.264811
C	9.286458	7.740202	1.604815

C	-5.316163	6.533566	3.120895
C	-6.668356	7.207624	3.228112
C	-7.805524	6.383277	3.147008
C	-6.863652	8.577225	3.434332
C	-9.087521	6.925775	3.202089
C	-9.261034	8.313036	3.153923
C	-8.147753	9.131871	3.349622
C	9.417107	-2.767565	0.050546
C	10.408962	-3.447863	-0.914685
C	10.539613	-2.639642	-2.189609
C	10.615927	-3.215992	-3.463713
C	10.72793	-1.251268	-2.106814
C	10.588557	-2.396673	-4.600841
C	10.311866	-1.029811	-4.471354
C	10.399525	-0.452455	-3.202153
C	-4.134239	2.432952	-1.38768
C	-4.8114	3.784475	-1.371451
C	-4.285719	4.872592	-2.082082
C	-6.017285	3.968922	-0.679821
C	-6.505324	5.253926	-0.428726
C	-5.819462	6.361643	-0.932778
C	-4.782865	6.158232	-1.850676
C	7.292739	2.347961	-2.741831
C	7.80344	2.897951	-4.052728
C	7.057713	2.683717	-5.224432
C	9.06666	3.48535	-4.219826
C	7.50795	3.199119	-6.442732
C	9.299197	4.36506	-5.276976
C	8.616377	4.062431	-6.470692
C	1.394589	7.598411	-0.434982
C	1.739262	9.027398	-0.088132
C	3.061316	9.33394	0.286877
C	0.944045	10.143107	-0.400481

C	3.292038	10.543823	0.955954
C	2.271034	11.498344	1.068804
C	1.052353	11.280411	0.409643
C	6.83166	7.373763	6.391774
C	6.659268	8.675792	7.135051
C	7.543365	9.743023	6.921598
C	5.810626	8.742529	8.240748
C	7.159169	11.028911	7.291444
C	5.921259	11.169558	7.932235
C	5.544163	10.044361	8.722969
C	-2.541198	2.601089	-4.8204
C	-3.437675	2.012962	-5.885322
C	-2.923026	1.752504	-7.166392
C	-4.602956	1.320594	-5.529195
C	-3.821468	1.458792	-8.198394
C	-5.177362	1.278189	-7.887226
C	-5.548525	1.109018	-6.546043
C	-1.672302	-1.94143	-5.029424
C	-3.133735	-2.293838	-4.794624
C	-4.076533	-1.928472	-5.76638
C	-3.614463	-2.905182	-3.621505
C	-5.380322	-2.422447	-5.721862
C	-4.989539	-3.056358	-3.422231
C	-5.855033	-2.959862	-4.519079
C	3.120016	-4.59132	3.300264
C	3.815181	-5.930909	3.178348
C	3.355284	-7.109092	3.784884
C	5.179166	-5.931366	2.832858
C	3.789832	-8.330155	3.247061
C	4.999612	-8.337219	2.53818
C	5.750061	-7.152445	2.453174
C	0.631019	7.330108	6.007829
C	0.531948	7.782823	7.442794

C	-0.679144	7.690828	8.14307
C	1.683996	8.146108	8.159007
C	-0.811095	8.291582	9.401335
C	0.279138	8.962429	9.966232
C	1.548218	8.780997	9.400246
C	6.668428	-0.369319	4.147394
C	3.092392	6.474481	-3.790692
C	3.038036	5.961456	-5.210534
C	4.20417	5.718457	-5.947569
C	1.839885	5.439646	-5.722021
C	4.132468	5.614517	-7.337684
C	1.826644	4.918071	-7.019951
C	2.906213	5.193727	-7.878417
C	7.63584	-0.786645	5.286077
C	8.99963	-1.227903	4.802579
C	9.674907	-0.57365	3.75716
C	9.682928	-2.277981	5.442956
C	10.870398	-1.120366	3.277912
C	11.662155	-1.936034	4.081848
C	10.950179	-2.701768	5.027981
C	6.216616	-3.122531	-5.638543
C	6.614942	-2.436927	-6.92641
C	5.641056	-2.20566	-7.913499
C	7.806803	-1.709635	-7.011794
C	6.011461	-1.65463	-9.140854
C	7.318229	-1.185398	-9.325282
C	8.197562	-1.160612	-8.239166
C	-4.568611	3.431174	5.933559
C	-5.760146	2.600469	5.514754
C	-5.805024	1.864834	4.319975
C	-6.987187	2.765924	6.180537
C	-6.990374	1.204336	3.962922
C	-8.090112	1.186455	4.831904

C	-7.986352	1.804371	6.09793
C	-2.674393	-1.429152	7.124441
C	-3.643081	-0.696925	8.029584
C	-5.014944	-0.976624	7.938279
C	-3.205495	0.006568	9.16014
C	-5.922909	-0.158608	8.616074
C	-5.445705	0.956342	9.313232
C	-4.099481	0.941014	9.713009
C	8.96755	3.094558	5.189452
C	9.377142	3.21159	6.637432
C	8.92596	4.271028	7.439572
C	10.205866	2.248968	7.23495
C	9.103278	4.218596	8.823353
C	10.649403	2.40922	8.554539
C	10.145107	3.447383	9.347184
C	0.964613	0.582953	-7.486913
C	1.205749	-0.654059	-8.327922
C	0.671671	-1.89856	-7.967472
C	1.779033	-0.554337	-9.606363
C	2.182083	-1.712949	-10.277922
C	1.079006	-3.068007	-8.614956
C	1.797476	-2.974196	-9.810119
C	3.02069	-2.765461	6.742649
C	2.718024	-2.615924	8.220099
C	1.407051	-2.360135	8.64854
C	3.668833	-2.949977	9.18818
C	3.438066	-2.632106	10.52802
C	1.15777	-2.248462	10.02148
C	2.271656	-1.944423	10.86597
C	5.277131	4.003444	8.785171
C	5.060109	3.001088	9.887083
C	5.717782	1.764983	9.858957
C	4.308308	3.325238	11.020394

C	5.257312	0.725353	10.662608
C	4.153926	0.969025	11.488309
C	4.102746	2.321426	11.980341
C	0.020977	4.374549	10.213788
C	0.300184	4.771895	11.654391
C	0.110331	6.099427	12.076541
C	0.556951	3.834511	12.678325
C	0.661594	6.495714	13.302072
C	0.555337	4.225092	14.026493
C	0.584775	5.589789	14.367239
C	-0.647929	-7.343458	-2.386006
C	-1.927322	-8.14873	-2.267384
C	-1.823075	-9.538064	-2.072872
C	-3.202315	-7.586843	-2.111754
C	-2.954661	-10.347457	-2.1951
C	-4.350115	-8.385264	-2.17401
C	-4.231982	-9.776423	-2.16972
C	-4.335131	-1.140398	1.771906
C	-4.582402	-2.276694	2.746628
C	-5.623396	-2.188805	3.686899
C	-3.66702	-3.325202	2.95139
C	-6.035552	-3.353248	4.351714
C	-5.262725	-4.519044	4.296211
C	-4.097795	-4.527935	3.516931
C	4.610557	-6.417536	-2.675186
C	3.530111	-6.509979	-3.736717
C	3.134912	-5.415879	-4.529736
C	2.810946	-7.704668	-3.942795
C	2.216125	-5.652394	-5.560922
C	1.345428	-6.736553	-5.564054
C	1.960089	-7.91498	-5.0301
H	-7.385411	5.393726	0.202414
H	10.108046	-0.410308	-5.345693

H	-3.402233	4.7471	-2.71155
H	10.633425	-2.852799	-5.59204
H	3.81917	8.548311	0.315337
H	-9.955305	6.26436	3.15488
H	10.836412	-0.771318	-1.131257
H	9.567597	9.886429	1.820921
H	10.214351	0.608855	-3.018951
H	-7.674396	5.29946	3.08395
H	-10.261076	8.745413	3.091912
H	-3.737273	1.668628	10.442664
H	10.630868	-4.30281	-3.57337
H	4.631547	-3.362871	8.879271
H	2.097362	-1.616426	11.893014
H	10.43057	3.533818	10.396463
H	6.496863	1.583412	9.114491
H	2.714237	-1.627628	-11.227937
H	-0.092528	6.859899	11.316612
H	8.126303	10.447705	-0.11931
H	0.660198	2.771342	12.445969
H	7.725385	11.89749	6.951053
H	4.249754	-2.734932	11.252534
H	3.722344	0.160471	12.080399
H	5.607951	-0.293823	10.48826
H	7.191508	8.643636	-1.564559
H	8.423768	9.599595	6.289682
H	4.277922	10.743372	1.380559
H	0.765772	7.565615	13.51358
H	0.595615	3.460794	14.807217
H	0.741203	5.905969	15.401596
H	5.521861	12.157067	8.170487
H	-6.503216	3.105125	-0.219529
H	-6.156106	7.372517	-0.696776
H	5.276141	-1.576001	-9.943757

H	-4.308708	7.013397	-2.335945
H	8.528926	5.765593	1.305423
H	7.626634	-0.780763	-10.291102
H	4.797959	10.178497	9.510387
H	9.841582	7.472156	2.505483
H	-8.27079	10.214293	3.422343
H	-6.014015	9.244437	3.584747
H	4.628449	-2.59976	-7.787404
H	5.307989	7.862145	8.64204
H	9.186455	0.206822	3.168691
H	2.425761	12.398	1.667313
H	6.131482	2.10729	-5.165224
H	11.324275	-0.646658	2.400666
H	-1.891463	2.028975	-7.401176
H	9.717534	3.565122	-3.341672
H	-4.928002	1.779096	3.676359
H	0.005822	9.998712	-0.943853
H	9.178192	-0.6907	-8.340487
H	-7.061122	0.707284	2.993554
H	2.421503	-7.097948	4.353518
H	8.492566	-1.718463	-6.165401
H	0.288949	12.059602	0.373623
H	-3.478505	1.404203	-9.232991
H	-1.545916	7.236442	7.653293
H	12.67264	-2.216684	3.779346
H	5.169419	5.927843	-5.477414
H	6.969236	2.96769	-7.363403
H	-3.740528	-1.384043	-6.653196
H	3.277332	-9.262191	3.48985
H	10.173953	5.01717	-5.283445
H	-5.370713	-1.719646	7.220351
H	-8.964316	0.58199	4.57964
H	8.881457	4.570885	-7.40045

H	-2.964664	-3.085506	-2.753882
H	0.937052	5.411817	-5.107838
H	2.668107	8.053255	7.692777
H	0.113988	-1.974166	-7.034151
H	5.697012	-4.988276	2.635299
H	9.163903	-2.841165	6.223023
H	-4.903928	1.243685	-4.481728
H	-6.990419	3.442256	7.04452
H	-5.912183	1.141139	-8.681942
H	0.577066	-2.330218	7.936051
H	11.447493	-3.507774	5.572682
H	-1.779766	8.294602	9.905792
H	-8.826517	1.799546	6.794385
H	8.243324	5.008782	7.012417
H	-6.057176	-2.261174	-6.562376
H	5.027611	5.686721	-7.956102
H	0.745244	-4.040271	-8.246942
H	5.399076	-9.268588	2.132354
H	-6.994889	-0.293187	8.459039
H	-2.146046	0.05888	9.420279
H	10.552606	1.397724	6.64355
H	3.987403	4.352945	11.201768
H	-6.556891	0.775391	-6.295096
H	-5.359081	-3.411132	-2.458015
H	0.142981	-2.035413	10.367669
H	6.763579	-7.176459	2.047779
H	0.90669	4.489416	-7.424142
H	2.433041	9.168156	9.912942
H	0.170654	9.502056	10.912597
H	2.818164	4.98288	-8.94622
H	-6.906928	-3.233514	-4.416005
H	8.624779	4.974956	9.450916
H	2.083152	0.420666	-9.995988

H	3.517027	2.551412	12.874581
H	2.109334	-3.874785	-10.341392
H	11.384564	1.714078	8.964806
H	-6.133652	1.699595	9.719197
H	-0.831794	-9.999995	-2.046977
H	-3.332571	-6.503201	-2.108983
H	-2.845666	-11.433917	-2.166315
H	-5.334696	-7.915628	-2.132576
H	-5.120347	-10.408499	-2.216391
H	-6.916421	-3.318379	4.997135
H	-2.742123	-3.353441	2.368674
H	-6.244198	-1.292682	3.735352
H	-3.44709	-5.403764	3.495526
H	-5.601497	-5.426655	4.799124
H	3.636138	-4.444196	-4.46545
H	3.129112	-8.580506	-3.365977
H	1.946376	-4.779896	-6.167896
H	0.536067	-6.801916	-6.294544
H	1.503928	-8.898476	-5.170839
Au	6.64465	-2.628342	1.543676
H	-5.281269	6.058118	2.124998
H	6.970486	6.324292	-2.092673
H	6.872514	4.695372	0.538149
H	11.388956	-3.512767	-0.41174
H	-2.124519	1.675887	-0.959483
H	10.090522	-4.477062	-1.135718
H	9.181415	1.502259	-2.067036
H	2.297353	7.118737	-0.828333
H	9.385628	-1.690797	-0.124152
H	-5.284933	5.693895	3.831936
H	8.270344	5.270908	-1.527764
H	8.784216	3.058227	-1.329557
H	7.820922	6.951807	6.647443

H	-3.552323	7.16504	4.255509
H	6.74803	1.410894	-2.928546
H	6.115002	4.203233	-0.983796
H	9.705976	-3.038689	1.073412
H	-2.255181	3.455557	-0.906197
H	6.53526	3.04613	-2.354666
H	-3.968195	2.099355	-2.424758
H	1.184432	7.033478	0.50473
H	0.472304	7.32769	-2.415904
H	-4.252163	8.459819	3.256943
H	6.058625	6.641537	6.704084
H	-4.75931	1.669519	-0.904555
H	-1.376494	-2.261508	-2.893105
H	2.78381	5.773729	-1.740674
H	4.613913	-2.909551	-4.185196
H	7.182659	6.599087	4.379108
H	-3.940622	3.901571	3.900467
H	-1.408028	0.755052	-4.951051
H	1.765656	4.950941	-2.93274
H	-0.569617	8.11605	-1.210724
H	3.722168	-3.911051	3.924524
H	-0.476429	-1.230404	7.089026
H	-1.941636	3.406097	-5.272671
H	-0.65753	-0.773278	-3.472455
H	7.234544	0.091952	3.33553
H	-2.988688	2.612561	4.662656
H	1.369914	6.51979	5.898036
H	7.174611	-1.573938	5.899021
H	-1.618486	-1.033456	-5.646043
H	-0.331963	6.923245	5.664387
H	7.318289	8.447916	4.674844
H	-1.333412	0.283044	7.439796
H	-1.992276	1.094438	-3.30716

H	4.512155	-1.788969	-5.555301
H	7.174358	1.85268	4.897185
H	3.140405	-4.131525	2.297393
H	1.980504	8.937041	5.481469
H	2.361362	7.291828	-3.668678
H	-1.237597	-2.747492	-5.646085
H	4.083877	6.899519	-3.574681
H	0.278767	9.27452	5.085841
H	-4.112891	2.985156	6.836098
H	1.57981	-4.334526	4.848596
H	-3.140477	3.066161	-4.028707
H	-2.410644	-2.399014	7.58232
H	7.097427	-3.338358	-5.01466
H	9.507707	2.269439	4.704532
H	5.931952	0.225027	4.699825
H	5.735785	-4.087945	-5.871437
H	7.766493	0.085395	5.952995
H	6.942154	3.249678	5.943407
H	1.226888	-5.594669	3.655352
H	-3.175088	-1.673379	6.176231
H	2.055833	2.350437	-6.844947
H	-4.92221	4.429644	6.240181
H	1.51203	-1.289075	6.127191
H	2.576295	-1.841548	4.830301
H	9.258939	4.015211	4.656837
H	0.271044	1.260622	-8.016301
H	2.862523	1.444275	-8.147995
H	5.133251	3.752978	6.589191
H	2.527528	-3.68036	6.373708
H	4.97629	5.01056	9.110866
H	0.5001	0.317209	-6.526067
H	4.100666	-2.905255	6.586466
H	4.201839	2.573306	7.529017

H	1.053313	2.463229	10.530113
H	0.158332	5.238227	9.546229
H	6.352984	4.051644	8.552196
H	-1.041814	4.081602	10.129694
H	1.88267	3.581951	9.436142
H	-0.174374	-7.610366	-3.34406
H	0.063287	-7.682935	-1.615101
H	-0.128356	-5.34091	-3.087993
H	-1.788807	-5.457779	-2.48891
H	-4.815031	-0.221725	2.14038
H	-2.579594	0.129482	1.822888
H	-2.201636	-1.560256	2.018271
H	-4.856346	-1.394791	0.831588
H	5.567154	-6.722851	-3.129673
H	5.810037	-4.660236	-2.200895
H	4.406068	-7.138861	-1.869691
H	4.162257	-4.279942	-2.673789
**B2-C**			
Au	-0.082352	0.001834	-0.077825
Au	2.711887	0.006741	-0.012772
Au	1.240157	2.476429	-0.029346
Au	1.460897	-1.848036	-1.64323
Au	1.301562	0.268875	2.395813
Au	0.217561	4.826966	1.056899
Au	3.102857	4.506318	0.833598
Au	3.831001	2.333152	-1.168887
Au	-0.864834	2.107701	1.832
Au	1.440969	0.962222	-2.368851
Au	1.651284	3.016107	2.697524
Au	5.558401	0.05459	-0.034514
Au	4.124321	-2.342681	-0.155161
Au	4.131525	1.805947	1.638926
Au	1.503472	-2.212649	1.179067

Au	3.998311	-0.407376	-2.452473
Au	3.907032	-0.932746	2.333844
S	6.045417	-0.806345	-3.754856
Au	1.980106	5.724754	3.251366
Au	-0.607786	4.301924	3.683673
Au	4.162745	3.89814	3.588497
Au	0.080682	1.563106	4.534478
Au	3.122704	1.27804	4.30767
Au	1.824414	3.581583	5.413894
S	4.80612	6.030222	-0.027703
S	-0.84276	5.692742	-0.913789
S	5.730261	-1.752086	3.700805
S	-2.414543	-1.060396	-0.077963
S	7.641252	1.187093	0.106312
S	6.588476	3.965176	3.610516
S	0.358526	-3.667055	2.68153
S	0.122873	2.317049	-3.864388
S	-1.400293	-0.207072	5.237215
S	2.908308	4.699875	7.254784
S	4.275069	-4.845196	-0.076985
Au	-1.11835	-3.091581	-0.515882
C	-3.047834	-1.390537	1.627533
Au	1.991268	-4.728975	-0.496949
C	4.736114	-4.84501	-1.893772
Au	4.746827	6.719572	2.328824
C	6.3733	5.101885	-0.212185
Au	7.127759	2.46357	1.886602
C	8.091153	2.291939	-1.327248
C	7.646219	3.576875	5.067432
Au	-0.249456	3.98872	-2.257962
C	-0.032406	7.26556	-1.476078
C	-1.466361	1.506083	-4.276048
Au	-0.427823	-1.790255	3.902351

C	-1.369328	-0.949462	6.934445
C	1.553929	-4.590696	3.778142
Au	3.797956	6.066149	5.798619
C	4.504439	3.719339	7.306499
S	4.873199	7.70261	4.439029
C	6.595879	7.281059	5.04932
S	-0.214591	-5.150913	-1.003806
C	-1.090502	-6.547494	-0.177383
S	-3.125358	2.361708	0.8258
S	1.453867	8.065473	3.185616
Au	-2.860651	4.757665	1.620008
C	-2.864616	2.393994	-0.989945
Au	-0.716091	7.336313	2.705481
C	1.221855	8.910427	4.821169
S	-2.960205	6.975484	2.228312
C	-4.197108	7.318373	3.539939
S	0.608403	-2.71502	-3.76503
S	3.95952	4.064205	-2.858026
Au	2.016699	-0.968101	-4.736111
C	-1.02139	-1.843922	-3.724903
Au	3.602162	2.24402	-4.365116
C	2.632003	5.282167	-3.059532
S	3.288122	0.574021	-5.937959
C	1.938168	1.228387	-7.044412
S	-2.406574	4.854489	5.218175
S	3.623954	-0.120953	6.177081
Au	-1.097065	3.386754	6.700507
C	-3.95063	3.859062	5.306088
Au	1.669215	1.121049	7.227453
C	2.674919	-1.6699	5.922758
S	-0.113079	2.060791	8.355987
C	0.541815	3.186458	9.682814
Au	6.728704	-2.124039	-1.896986

C	5.726496	-2.078389	-5.07272
S	7.593549	-3.533373	-0.226208
C	7.39302	5.986854	-0.936876
C	8.241994	6.782892	0.029956
C	8.906251	6.169157	1.10599
C	8.549801	8.13066	-0.212382
C	9.313487	6.95706	2.186243
C	9.616931	8.305422	1.957985
C	9.2599	8.880271	0.733626
C	-3.848201	8.618484	4.274578
C	-4.203123	8.698239	5.747956
C	-3.80059	9.849743	6.448841
C	-4.640873	7.612666	6.514481
C	-4.321191	10.117969	7.713778
C	-5.097931	9.141544	8.351612
C	-5.137259	7.855438	7.805833
C	9.400088	-3.145175	-0.138047
C	10.110127	-3.415025	-1.484587
C	11.006323	-2.302021	-2.009401
C	11.884212	-2.592913	-3.069115
C	11.156409	-1.055223	-1.383035
C	12.675933	-1.589808	-3.637544
C	12.712431	-0.307981	-3.075047
C	11.871503	-0.018441	-1.997563
C	-4.17656	2.05361	-1.711442
C	-5.1563	3.201516	-1.765
C	-5.045121	4.192317	-2.754738
C	-5.972863	3.512289	-0.665338
C	-6.995402	4.45603	-0.833139
C	-7.088418	5.208432	-2.009451
C	-6.121115	5.045473	-3.009539
C	7.235961	2.081182	-2.572448
C	7.948568	2.082769	-3.903671

C	7.295033	2.574323	-5.045959
C	9.170797	1.419311	-4.075256
C	7.81968	2.344789	-6.321643
C	9.76325	1.310196	-5.335343
C	9.077587	1.753829	-6.470561
C	1.303762	7.633808	-0.847475
C	1.980887	8.809145	-1.506563
C	1.344286	9.696251	-2.387619
C	3.377021	8.976191	-1.439297
C	2.110177	10.68727	-3.031237
C	3.39531	10.989769	-2.534972
C	3.944403	10.237003	-1.450745
C	7.245577	8.578082	5.587006
C	7.053893	8.746187	7.076419
C	6.55195	9.934025	7.625288
C	7.455609	7.745977	7.980278
C	6.352144	10.027527	9.008324
C	6.903473	9.095698	9.886519
C	7.185005	7.821644	9.345846
C	-2.392686	2.381003	-5.147151
C	-2.891598	1.558791	-6.305891
C	-4.149505	0.938317	-6.287649
C	-2.110517	1.384041	-7.463021
C	-4.367886	-0.163969	-7.120509
C	-3.743325	-0.152851	-8.374246
C	-2.617974	0.668612	-8.552277
C	-1.918197	-2.133909	-4.940974
C	-3.288253	-2.610923	-4.515597
C	-3.993847	-3.573016	-5.251726
C	-4.058969	-1.834599	-3.629171
C	-5.123261	-4.159482	-4.657737
C	-5.1454	-2.419537	-2.975522
C	-5.704772	-3.583408	-3.521732

C	3.03538	-4.360434	3.4853
C	3.946467	-5.567569	3.551197
C	3.509583	-6.880078	3.325807
C	5.277847	-5.384819	3.955107
C	4.439227	-7.914578	3.191918
C	5.802451	-7.619553	3.148688
C	6.210458	-6.400574	3.713209
C	0.657957	7.928285	5.85469
C	0.951351	8.140604	7.317109
C	2.066142	8.854979	7.780744
C	-0.022453	7.801234	8.26729
C	2.545639	8.584572	9.067127
C	1.797962	7.738217	9.89492
C	0.44717	7.522736	9.56402
C	6.747999	-0.192156	3.684686
C	2.940637	6.207614	-4.240461
C	1.69419	6.76247	-4.894978
C	0.560051	5.97403	-5.156973
C	1.613266	8.103256	-5.283195
C	-0.276972	6.285177	-6.222885
C	0.40508	8.599751	-5.778128
C	-0.33006	7.66246	-6.553516
C	7.793618	-0.211111	4.815775
C	9.180559	-0.638957	4.378853
C	10.118898	-1.076469	5.326749
C	9.705944	-0.179733	3.154384
C	11.404076	-1.44551	4.909094
C	11.82282	-1.218069	3.59353
C	10.924668	-0.659672	2.67454
C	6.658186	-1.85986	-6.264606
C	6.097259	-2.43819	-7.542314
C	4.760449	-2.283803	-7.95858
C	6.946813	-2.938649	-8.55279

C	4.390519	-2.906604	-9.167028
C	5.296948	-3.362684	-10.127735
C	6.557563	-3.786393	-9.57972
C	-4.071443	2.470306	4.70547
C	-5.433844	2.303043	4.023125
C	-6.267393	3.376688	3.658818
C	-5.921616	1.030945	3.698827
C	-7.249835	3.248884	2.680304
C	-7.708837	1.953427	2.4067
C	-7.213429	0.887949	3.187558
C	-2.554738	-1.894341	7.137573
C	-3.788901	-1.284081	7.774309
C	-3.822611	-0.000202	8.33942
C	-5.019488	-1.946156	7.634107
C	-4.93575	0.394645	9.09469
C	-6.08687	-0.395417	9.136991
C	-6.107242	-1.609485	8.439689
C	9.117129	3.572638	4.651905
C	10.134362	3.858691	5.730402
C	11.300566	3.079055	5.818213
C	9.877804	4.671881	6.843166
C	12.30916	3.507077	6.697561
C	10.950859	5.402871	7.364939
C	12.204969	4.762213	7.321405
C	2.459913	1.786493	-8.371139
C	3.157738	3.113082	-8.153718
C	2.373642	4.257021	-7.910223
C	4.477519	3.17878	-7.681492
C	5.014494	4.461275	-7.459354
C	2.968986	5.48517	-8.223515
C	4.364542	5.575138	-8.009404
C	3.193748	-2.741243	6.873717
C	2.890147	-2.328157	8.302395

C	1.577458	-2.151742	8.7569
C	3.924393	-2.053138	9.206762
C	3.645176	-1.483337	10.453837
C	1.294638	-1.990395	10.108839
C	2.315253	-1.401798	10.885784
C	5.413313	4.202158	8.436136
C	6.361159	3.146696	8.962932
C	6.057642	1.772763	8.972567
C	7.722548	3.459144	9.145477
C	6.789354	0.951649	9.841841
C	8.114658	1.288076	10.142253
C	8.581299	2.560125	9.787257
C	1.894607	2.71026	10.235114
C	1.979033	2.226803	11.659512
C	3.246145	2.181368	12.268167
C	0.883934	1.924629	12.468889
C	3.524131	1.233753	13.241957
C	1.154244	1.450654	13.764652
C	2.382506	0.746926	13.925067
C	-2.028496	-6.171689	0.978131
C	-2.034508	-7.178778	2.104148
C	-3.23027	-7.723144	2.587944
C	-0.830564	-7.617466	2.677527
C	-3.253777	-8.449042	3.782384
C	-0.83953	-8.4519	3.796046
C	-2.052299	-8.782072	4.412579
C	-4.497424	-1.885096	1.534642
C	-4.918952	-2.576839	2.819778
C	-4.279796	-3.788859	3.188244
C	-6.09212	-2.329087	3.530813
C	-4.984148	-4.782348	3.855686
C	-5.920801	-4.294767	4.808267
C	-6.315359	-2.941442	4.768752

C	4.986238	-6.26465	-2.456063
C	4.043018	-6.507566	-3.609547
C	3.137412	-7.578703	-3.638584
C	4.147957	-5.769013	-4.805155
C	1.965966	-7.423688	-4.388629
C	2.107318	-6.77879	-5.628269
C	3.262181	-6.012968	-5.86137
H	-7.707165	4.631649	-0.024388
H	13.331436	0.475827	-3.523509
H	-4.260463	4.099915	-3.510847
H	13.321825	-1.828945	-4.485423
H	0.284408	9.598751	-2.627285
H	-4.125925	11.076454	8.197573
H	10.539443	-0.804403	-0.518124
H	10.067546	8.915344	2.742879
H	11.888356	0.975101	-1.540999
H	-3.242556	10.626959	5.918767
H	-5.549153	9.340499	9.325132
H	-7.012133	-2.220327	8.422872
H	11.866121	-3.589119	-3.522108
H	4.961131	-2.186721	8.887349
H	2.091657	-1.016933	11.883212
H	13.069944	5.194189	7.827552
H	5.073099	1.420872	8.651733
H	6.036542	4.56513	-7.08831
H	4.082525	2.630389	11.719662
H	9.625003	6.481488	3.119315
H	-0.140733	2.137374	12.162054
H	5.948913	10.961173	9.411154
H	4.449498	-1.1101	11.089768
H	8.748867	0.603832	10.708754
H	6.419057	-0.052927	10.058303
H	8.790194	5.091903	1.262756

H	6.19303	10.728575	6.967298
H	1.673587	11.29867	-3.821974
H	4.538761	0.943523	13.512009
H	0.332285	1.25519	14.456962
H	2.50573	0.082003	14.783719
H	6.816746	9.235213	10.965691
H	-5.950236	2.885537	0.227584
H	-7.922117	5.898412	-2.152835
H	3.34869	-2.777893	-9.481647
H	-6.124373	5.680895	-3.896545
H	8.158429	8.61921	-1.108264
H	4.923903	-3.760674	-11.075131
H	7.459414	6.990128	9.999301
H	9.497165	9.926232	0.529929
H	-5.59833	7.039514	8.366364
H	-4.766907	6.623002	6.077568
H	3.986699	-1.877492	-7.305458
H	7.840864	6.804469	7.577687
H	9.799642	-1.280744	6.352029
H	3.904185	11.885881	-2.899976
H	6.328262	3.071104	-4.93798
H	12.108726	-1.855484	5.635698
H	-4.790753	1.077746	-5.411658
H	9.681245	0.974645	-3.218348
H	-6.013698	4.399368	3.936991
H	3.912413	8.197421	-0.881094
H	7.300528	-4.315051	-10.180485
H	-7.710411	4.128582	2.23204
H	2.451071	-7.101932	3.185913
H	8.004291	-3.007199	-8.254385
H	4.945708	10.43293	-1.065159
H	-5.218776	-0.823077	-6.936225
H	2.715358	9.381955	7.079291

H	12.81734	-1.535284	3.274001
H	0.496189	4.969838	-4.718458
H	7.274336	2.68481	-7.205693
H	-3.51805	-4.058024	-6.10858
H	4.088928	-8.921411	2.953282
H	10.733501	0.816969	-5.430036
H	-2.936345	0.636556	8.350057
H	-8.563859	1.796968	1.745461
H	9.508748	1.620832	-7.466251
H	-3.618934	-0.930188	-3.198377
H	2.402819	8.790585	-4.971234
H	-1.029059	7.509293	7.959352
H	1.282396	4.171665	-7.959974
H	5.611927	-4.411898	4.319078
H	9.0588	0.390083	2.472774
H	-1.148182	1.896115	-7.546215
H	-5.374678	0.151331	4.047973
H	-4.046977	-0.848587	-9.158112
H	0.761677	-2.195189	8.024131
H	11.252247	-0.419056	1.661132
H	3.519891	8.964042	9.381294
H	-7.653864	-0.099554	3.060706
H	11.455263	2.22449	5.155887
H	-5.600742	-5.019771	-5.132036
H	-0.980496	5.568353	-6.646294
H	2.368133	6.379038	-8.398325
H	6.535136	-8.393215	2.913882
H	-4.922774	1.36198	9.601431
H	-5.04444	-2.875478	7.061325
H	8.876406	5.099058	6.945959
H	8.066494	4.478354	8.958805
H	-2.078951	0.66269	-9.501296
H	-5.626787	-1.908008	-2.139715

H	0.261436	-1.970272	10.461198
H	7.2707	-6.207759	3.890561
H	0.254332	9.67072	-5.926352
H	-0.226234	7.07742	10.299908
H	2.177642	7.435701	10.872438
H	-1.096635	8.020455	-7.245756
H	-6.566684	-4.057774	-3.048531
H	13.237221	2.937939	6.778293
H	5.079165	2.278759	-7.549029
H	9.622957	2.837351	9.960413
H	4.878714	6.53423	-8.089098
H	10.788482	6.287408	7.98322
H	-6.942175	-0.088483	9.741557
H	-4.168761	-7.464006	2.091352
H	0.122042	-7.278458	2.262803
H	-4.205717	-8.777311	4.204119
H	0.104016	-8.773699	4.241812
H	-2.05744	-9.362493	5.336439
H	-4.609898	-5.804137	3.90663
H	-6.699743	-1.46345	3.281519
H	-3.370136	-4.072375	2.647786
H	-7.056324	-2.550658	5.466727
H	-6.306941	-4.958152	5.586181
H	3.118321	-8.249981	-2.772252
H	4.933136	-5.019861	-4.924748
H	1.09581	-8.053076	-4.190895
H	1.304869	-6.803073	-6.367596
H	3.404231	-5.503167	-6.816415
Au	6.598165	-2.614857	1.61535
H	-2.767708	8.811774	4.162831
H	6.882014	6.659614	-1.640183
H	6.724277	4.758768	0.766473
H	10.706867	-4.335085	-1.403789

H	-2.090411	1.642434	-1.198573
H	9.350213	-3.611787	-2.262135
H	9.141142	2.029552	-1.5139
H	1.982018	6.770618	-0.822107
H	9.574001	-2.178627	0.333957
H	-4.350128	9.456579	3.763457
H	8.046495	5.339469	-1.549248
H	8.064649	3.32308	-0.966313
H	6.86619	9.456865	5.047747
H	-5.159743	7.364153	3.0157
H	6.735624	1.102448	-2.476981
H	6.103656	4.221492	-0.810717
H	9.780125	-3.978976	0.470201
H	-2.493214	3.380278	-1.290267
H	6.412221	2.81165	-2.590451
H	-3.940202	1.734433	-2.73794
H	1.141283	7.875138	0.218799
H	0.056374	7.180209	-2.567644
H	-4.201221	6.465733	4.225721
H	8.328229	8.522547	5.374744
H	-4.640362	1.186277	-1.215084
H	-1.505825	-2.237245	-2.816919
H	2.605582	5.832839	-2.117247
H	5.841209	-3.075336	-4.634739
H	6.494191	6.610514	5.905783
H	-4.186976	3.83353	6.378047
H	-1.156517	0.629647	-4.856794
H	1.671853	4.763799	-3.167754
H	-0.79059	8.030294	-1.255869
H	3.439137	-3.578612	4.13841
H	-0.462044	-1.561181	7.046913
H	-1.829704	3.255245	-5.507833
H	-0.832493	-0.785403	-3.545052

H	7.303815	-0.181807	2.749765
H	-4.665786	4.542374	4.844886
H	1.070347	6.931007	5.612183
H	7.451563	-0.840576	5.651514
H	-2.014006	-1.229319	-5.565468
H	-0.429071	7.824425	5.702164
H	7.166665	6.864954	4.214997
H	-1.321049	-0.13473	7.66205
H	-1.958478	1.153035	-3.362261
H	4.675264	-1.952744	-5.355983
H	7.340739	2.633565	5.536393
H	3.124174	-3.945715	2.46472
H	2.226899	9.263915	5.083704
H	3.593069	7.029422	-3.914219
H	-1.450826	-2.899592	-5.577588
H	3.491475	5.636801	-5.007958
H	0.563396	9.768441	4.642307
H	-3.289883	2.317147	3.941695
H	1.298027	-4.344714	4.815213
H	-3.232154	2.757458	-4.550692
H	-2.214362	-2.70396	7.809515
H	6.805813	-0.77163	-6.391034
H	9.355292	2.617275	4.159736
H	6.060309	0.647399	3.824214
H	7.652423	-2.281636	-6.061245
H	7.872504	0.807212	5.228001
H	7.428191	4.384016	5.778907
H	1.260692	-5.632596	3.608568
H	-2.821224	-2.386018	6.18928
H	1.276447	0.370362	-7.226202
H	-3.918482	1.693052	5.466328
H	1.606066	-1.471458	6.055407
H	2.819181	-1.945999	4.869869

H	9.246919	4.345231	3.873173
H	1.60478	1.915764	-9.052525
H	1.377597	1.981033	-6.474782
H	4.992988	3.768683	6.329099
H	2.710916	-3.703494	6.637599
H	4.784841	4.547517	9.276632
H	3.147339	1.062339	-8.835725
H	4.278044	-2.866797	6.742199
H	4.195346	2.677025	7.45695
H	0.632452	4.189632	9.251652
H	2.243946	1.870503	9.600586
H	5.974263	5.093369	8.114518
H	2.64269	3.502242	10.075784
H	-0.252944	3.203403	10.438088
H	-1.684665	-5.209941	1.396041
H	-3.047879	-6.004853	0.604145
H	-0.305369	-7.239055	0.149243
H	-1.64058	-7.027465	-0.997642
H	-5.162234	-1.034246	1.324653
H	-2.985061	-0.445678	2.185017
H	-2.391115	-2.118448	2.117237
H	-4.588337	-2.597859	0.700472
H	6.036312	-6.344885	-2.776732
H	5.64771	-4.239064	-1.976828
H	4.823722	-7.018672	-1.673672
H	3.948863	-4.330155	-2.451823
**B2-D**			
Au	0.048337	0.005971	-0.000689
Au	2.853236	0.029768	-0.001541
Au	1.353107	2.478913	-0.07522
Au	1.57795	-1.900195	-1.535554
Au	1.498455	0.362264	2.426801
Au	0.331108	4.857625	0.948454

Au	3.213729	4.55699	0.669396
Au	3.917291	2.328852	-1.267976
Au	-0.714352	2.156484	1.847959
Au	1.514189	0.891847	-2.360529
Au	1.819654	3.131745	2.62091
Au	5.688173	0.091076	-0.090653
Au	4.277951	-2.325312	-0.092664
Au	4.290096	1.884277	1.548804
Au	1.677891	-2.16234	1.297253
Au	4.076916	-0.477254	-2.4548
Au	4.108091	-0.816868	2.349238
S	6.016007	-0.96798	-3.785803
Au	2.123032	5.860004	3.06859
Au	-0.437512	4.419516	3.611332
Au	4.343369	4.060469	3.418632
Au	0.298804	1.720135	4.545298
Au	3.337955	1.456635	4.257506
Au	2.051037	3.788166	5.308241
S	4.794999	6.167147	-0.280819
S	-0.682464	5.922637	-0.995044
S	5.964953	-1.694604	3.66203
S	-2.302055	-0.902461	0.09654
S	7.81426	1.252837	0.069359
S	6.764063	4.193047	3.363301
S	0.450479	-3.521881	2.875625
S	0.051716	2.190296	-3.820337
S	-0.965197	-0.117126	5.579037
S	3.010428	4.931139	7.249051
S	4.361391	-4.761861	0.066604
Au	-0.973973	-3.076072	-0.30701
C	-2.783256	-1.076388	1.858951
Au	2.107125	-4.749894	-0.287885
C	4.635446	-5.629972	-1.566717

Au	4.850178	6.884491	2.110285
C	6.310852	5.23964	-0.72568
Au	7.197188	2.57398	1.707362
C	8.224405	2.299774	-1.413899
C	7.801792	3.52911	4.740623
Au	-0.230457	3.956103	-2.321941
C	0.331025	7.353257	-1.590759
C	-1.561026	1.431503	-4.284412
Au	-0.165909	-1.650724	4.019356
C	-2.791607	-0.335323	5.5042
C	1.667667	-4.509299	3.884849
Au	4.026445	6.310425	5.503143
C	4.229159	3.608772	7.669834
S	5.192233	7.847981	4.203604
C	7.019052	7.787099	4.495709
S	-0.131329	-5.17971	-0.776948
C	-1.035119	-6.381569	0.301732
S	-3.038917	2.286897	1.048819
S	1.530759	8.203421	3.117018
Au	-2.731858	4.786097	1.587652
C	-2.645088	2.233589	-0.743737
Au	-0.57631	7.443685	2.426079
C	0.982271	8.708817	4.823163
S	-2.802585	7.075995	1.872553
C	-3.781685	7.296745	3.413338
S	0.815277	-2.817516	-3.634522
S	4.066163	3.968499	-3.045729
Au	2.073118	-1.154674	-4.674567
C	-0.838269	-1.978215	-3.653399
Au	3.574697	2.076497	-4.462127
C	2.654302	5.114993	-2.989626
S	3.259412	0.365442	-6.008454
C	2.121476	0.984125	-7.332995

S	-2.16456	4.802	5.250894
S	3.791818	0.122055	6.229134
Au	-0.982329	3.624466	6.627235
C	-3.795631	3.932853	5.258867
Au	1.912856	1.427477	7.224324
C	3.065598	-1.520531	5.884753
S	0.085224	2.371918	8.315965
C	0.724874	3.560169	9.574509
Au	6.862521	-2.150854	-1.888145
C	5.886746	-2.255252	-5.085707
S	7.874961	-3.423476	-0.194644
C	7.394735	6.176578	-1.257518
C	8.18877	6.89012	-0.187182
C	9.002949	6.153659	0.686648
C	8.362817	8.277113	-0.232573
C	9.586064	6.776726	1.787959
C	9.384375	8.148682	1.974349
C	8.997494	8.903326	0.852964
C	-3.942784	8.77139	3.780921
C	-4.208874	8.955125	5.260396
C	-4.025921	10.208677	5.870717
C	-4.971055	8.0389	6.009423
C	-3.791968	10.220006	7.252952
C	-4.33627	9.196781	8.041823
C	-4.940382	8.103187	7.410146
C	9.596434	-2.745332	-0.036624
C	9.810475	-1.503725	-0.901527
C	11.24502	-1.187182	-1.233654
C	12.18081	-2.170402	-1.593116
C	11.708992	0.138799	-1.167108
C	13.409189	-1.792612	-2.141344
C	13.940141	-0.546167	-1.784792
C	13.075802	0.420845	-1.253502

C	-3.92178	1.978333	-1.540769
C	-4.842868	3.178523	-1.474061
C	-4.322303	4.435812	-1.838926
C	-6.131312	3.133512	-0.942142
C	-7.047562	4.137612	-1.271001
C	-6.530298	5.357049	-1.740456
C	-5.131481	5.564579	-1.737273
C	7.199007	2.278738	-2.543002
C	7.753023	2.154038	-3.945507
C	7.066661	2.76935	-5.003616
C	8.942687	1.475854	-4.248275
C	7.359692	2.438261	-6.327649
C	9.219277	1.107846	-5.569091
C	8.393465	1.541158	-6.610323
C	1.697908	7.580578	-0.953332
C	1.961866	8.971757	-0.397903
C	3.297996	9.380075	-0.21128
C	1.022119	10.011907	-0.336043
C	3.53861	10.559215	0.506222
C	2.472297	11.210933	1.146922
C	1.165686	10.932485	0.713677
C	7.399218	7.121609	5.815842
C	8.553111	7.707754	6.593661
C	9.29966	8.83126	6.216957
C	8.857756	7.1733	7.859395
C	10.167881	9.431637	7.140225
C	10.668136	8.620815	8.177353
C	10.129878	7.326275	8.393721
C	-2.551988	2.519753	-4.685566
C	-3.752574	2.080975	-5.498183
C	-4.947536	2.816335	-5.369869
C	-3.847099	0.841168	-6.144407
C	-6.045991	2.482805	-6.169001

C	-5.889394	1.553419	-7.208024
C	-4.775836	0.703489	-7.185928
C	-1.747515	-2.460925	-4.800415
C	-3.002428	-3.115379	-4.276067
C	-3.817997	-2.455235	-3.340579
C	-3.328404	-4.437676	-4.596544
C	-4.780303	-3.162192	-2.622599
C	-4.571528	-4.964828	-4.236271
C	-5.2158	-4.398324	-3.126605
C	3.137421	-4.179196	3.650237
C	4.119092	-5.321277	3.697866
C	5.328192	-5.176205	4.402505
C	3.803135	-6.645377	3.343035
C	6.420087	-5.936731	3.959481
C	6.174904	-7.152286	3.305714
C	4.852457	-7.518714	3.022807
C	0.410534	7.586133	5.694158
C	0.574502	7.755148	7.184706
C	-0.513286	7.479244	8.027799
C	1.810874	8.050983	7.775589
C	-0.367878	7.55362	9.414826
C	0.761887	8.162297	9.964457
C	1.895927	8.287334	9.151146
C	6.952147	-0.131872	3.841045
C	2.877271	6.280236	-3.958972
C	1.687949	6.503378	-4.876846
C	1.161645	7.792381	-5.056622
C	0.938366	5.422169	-5.37113
C	-0.014012	8.000763	-5.781227
C	-0.142935	5.637514	-6.227778
C	-0.621888	6.93216	-6.444446
C	8.088439	-0.348846	4.85609
C	9.300993	-1.046758	4.287876

C	10.005152	-2.01671	5.013769
C	9.920552	-0.565791	3.113688
C	11.007655	-2.746356	4.360056
C	11.761163	-2.134923	3.358871
C	11.13101	-1.09459	2.654339
C	7.292498	-2.325644	-5.737039
C	8.09783	-3.526042	-5.310472
C	9.301378	-3.352549	-4.608432
C	7.741545	-4.834659	-5.672944
C	10.068391	-4.46931	-4.264476
C	9.450303	-5.715187	-4.169914
C	8.354846	-5.918875	-5.030192
C	-3.959815	2.703291	4.396127
C	-5.386602	2.413679	3.986594
C	-5.819866	1.095573	3.79041
C	-6.325585	3.416991	3.691398
C	-7.127349	0.82682	3.375413
C	-7.839842	1.852011	2.732558
C	-7.342225	3.167645	2.770497
C	-3.223912	-1.667503	6.125691
C	-3.583676	-1.624352	7.597303
C	-3.543477	-2.816735	8.333946
C	-4.212597	-0.519751	8.183015
C	-3.720081	-2.780136	9.716957
C	-3.735222	-1.544121	10.361681
C	-4.313864	-0.507029	9.58858
C	9.286576	3.61999	4.421887
C	10.163133	3.164636	5.570373
C	11.43067	2.632846	5.296804
C	9.871267	3.469787	6.904478
C	12.094255	1.864787	6.249749
C	10.787613	3.077408	7.888479
C	11.633382	1.980615	7.573927

C	1.144636	-0.060871	-7.867339
C	-0.041383	0.580739	-8.565051
C	-0.582026	0.000316	-9.7232
C	-0.752147	1.6445	-7.983891
C	-1.87717	2.173859	-8.626911
C	-1.487022	0.729321	-10.508031
C	-2.120559	1.856102	-9.966239
C	3.477607	-2.522978	6.965532
C	2.440465	-2.753227	8.045853
C	1.06279	-2.716046	7.786537
C	2.849646	-2.878117	9.383206
C	1.905343	-3.018257	10.402007
C	0.154531	-3.113936	8.773645
C	0.590569	-3.36169	10.080844
C	4.903321	3.829325	9.019375
C	5.745782	2.621637	9.365951
C	5.595738	1.972771	10.596836
C	6.789728	2.21105	8.525382
C	6.179321	0.722469	10.811767
C	7.037598	0.19928	9.838828
C	7.526607	1.067713	8.844773
C	0.882094	2.851386	10.915736
C	1.122553	3.772052	12.088173
C	0.676537	5.10345	12.154745
C	2.027823	3.3932	13.110019
C	0.748143	5.720501	13.415654
C	2.237822	4.13841	14.274476
C	1.794206	5.484247	14.299419
C	-0.148903	-7.045689	1.351488
C	-0.914157	-7.831614	2.393861
C	-0.365935	-8.016976	3.673396
C	-2.029178	-8.620446	2.0687
C	-1.063061	-8.760015	4.632158

C	-2.901404	-9.025372	3.082655
C	-2.386325	-9.145985	4.380928
C	-4.238704	-1.538888	1.944823
C	-4.383445	-3.044543	1.971728
C	-3.704128	-3.815314	2.929439
C	-5.409369	-3.671394	1.253501
C	-3.639288	-5.202687	2.781117
C	-4.449895	-5.824222	1.823016
C	-5.410925	-5.067903	1.144393
C	4.401861	-4.810244	-2.835756
C	3.876501	-5.656938	-3.979992
C	2.859777	-6.611332	-3.78793
C	4.248421	-5.424202	-5.318072
C	2.473666	-7.387322	-4.888005
C	2.574287	-6.92893	-6.195507
C	3.764527	-6.176215	-6.394318
H	-8.114827	4.014154	-1.083617
H	14.981194	-0.300871	-2.000824
H	-3.234664	4.497003	-1.967009
H	14.054167	-2.547496	-2.596658
H	4.125029	8.735162	-0.510056
H	-3.384788	11.114593	7.728909
H	11.016219	0.925041	-0.855525
H	9.774084	8.648518	2.863063
H	13.44379	1.421997	-1.022291
H	-3.647315	11.039671	5.266072
H	-4.233528	9.221834	9.127851
H	-4.660131	0.403108	10.084382
H	11.857798	-3.210515	-1.683154
H	3.90711	-2.759756	9.631171
H	-0.12652	-3.623279	10.860927
H	12.141438	1.444356	8.378781
H	4.891209	2.378487	11.327994

H	-2.461667	2.958888	-8.141152
H	0.002184	5.513466	11.401885
H	10.106239	6.183997	2.54205
H	2.444704	2.381195	13.065319
H	10.604431	10.411526	6.945139
H	2.240217	-3.057348	11.441533
H	7.497292	-0.780768	9.981846
H	5.928117	0.143603	11.702213
H	9.051916	5.065339	0.59654
H	9.089118	9.343763	5.275917
H	4.566292	10.869221	0.706006
H	0.22424	6.680958	13.508341
H	2.938123	3.763309	15.024957
H	1.96312	6.125567	15.16593
H	11.515921	8.968501	8.77147
H	-6.487255	2.198319	-0.499867
H	-7.202577	6.200633	-1.912628
H	11.038567	-4.308206	-3.785616
H	-4.720736	6.560746	-1.911085
H	7.92162	8.867458	-1.037287
H	9.944994	-6.561933	-3.691878
H	10.494414	6.695853	9.20546
H	9.048339	9.993725	0.891493
H	-5.432949	7.328994	8.001405
H	-5.360821	7.134889	5.53497
H	9.690831	-2.345888	-4.44402
H	8.242346	6.332461	8.205581
H	9.5723	-2.407062	5.939163
H	2.66345	11.990052	1.886224
H	6.2141	3.41546	-4.784004
H	11.410526	-3.633554	4.858227
H	-5.002365	3.654488	-4.670766
H	9.576308	1.072488	-3.455924

H	-5.175709	0.275375	4.107932
H	0.012473	9.855129	-0.724379
H	7.954214	-6.923658	-5.179549
H	-7.50567	-0.196859	3.368717
H	5.523551	-4.234215	4.923917
H	6.913397	-5.014114	-6.359245
H	0.323369	11.545472	1.03743
H	-6.974906	3.049952	-6.077878
H	-1.458214	7.146767	7.59225
H	12.675164	-2.593154	2.978029
H	1.665935	8.643116	-4.590414
H	6.760138	2.860754	-7.136257
H	-3.575378	-1.432436	-3.039266
H	7.432451	-5.675685	4.274922
H	10.091353	0.486589	-5.784192
H	-3.310913	-3.758153	7.831332
H	-8.765923	1.62507	2.200585
H	8.597097	1.237536	-7.638795
H	-2.698511	-4.975573	-5.310835
H	1.144916	4.393114	-5.05092
H	2.70465	8.159894	7.156166
H	-0.120529	-0.905395	-10.129241
H	2.797739	-6.903409	3.001871
H	9.443717	0.225986	2.529642
H	-3.012364	0.13891	-6.101267
H	-6.050297	4.461545	3.863883
H	-6.677569	1.41561	-7.951697
H	0.683719	-2.494813	6.786767
H	11.611408	-0.650854	1.779172
H	-1.227279	7.324188	10.051054
H	-7.895823	3.988141	2.312161
H	11.748825	2.570376	4.251521
H	-5.281956	-2.701624	-1.770818

H	-0.41832	9.00987	-5.879897
H	-1.756198	0.373343	-11.504637
H	7.00329	-7.790364	2.993792
H	-3.587672	-3.700111	10.292932
H	-4.527027	0.348557	7.603956
H	9.02369	4.102779	7.168071
H	7.048567	2.79329	7.639791
H	-4.699776	-0.131622	-7.884354
H	-4.903446	-5.924493	-4.636459
H	-0.905066	-3.198171	8.52879
H	4.632813	-8.501345	2.601254
H	-0.662084	4.783082	-6.667455
H	2.844534	8.612458	9.582921
H	0.83086	8.34942	11.037333
H	-1.501114	7.098114	-7.06944
H	-6.07836	-4.896607	-2.679069
H	12.922461	1.209079	5.98183
H	-0.459825	2.032055	-7.004983
H	8.346307	0.75381	8.195112
H	-2.885328	2.393705	-10.535572
H	10.605442	3.334064	8.934375
H	-3.699664	-1.467757	11.44938
H	0.595151	-7.566392	3.930198
H	-2.387852	-8.630613	1.035697
H	-0.624218	-8.923846	5.617972
H	-3.890929	-9.417561	2.842152
H	-2.994634	-9.57227	5.180327
H	-2.979853	-5.799499	3.414495
H	-6.093733	-3.073158	0.647193
H	-3.032283	-3.327789	3.64007
H	-6.134502	-5.561885	0.493035
H	-4.396253	-6.904008	1.68157
H	2.517157	-6.893528	-2.791466

H	5.061645	-4.725034	-5.50428
H	1.736927	-8.174429	-4.684479
H	2.085393	-7.451015	-7.020085
H	4.112995	-5.938594	-7.403284
Au	6.808112	-2.534414	1.611093
H	-3.009065	9.303787	3.536778
H	6.940881	6.906051	-1.945
H	6.652851	4.645788	0.134765
H	9.213185	-1.645573	-1.831456
H	-1.915065	1.431124	-0.892428
H	9.350914	-0.643872	-0.397294
H	9.183581	1.88427	-1.744076
H	2.508126	7.323384	-1.638555
H	9.746554	-2.48015	1.014362
H	-4.733773	9.234899	3.169164
H	8.08355	5.564203	-1.868083
H	8.413661	3.315424	-1.050453
H	7.596278	6.059823	5.61507
H	-4.747824	6.813578	3.213656
H	6.526922	1.414751	-2.390497
H	5.969072	4.556435	-1.506182
H	10.256571	-3.572012	-0.32784
H	-2.155629	3.171784	-1.028636
H	6.548463	3.15697	-2.491279
H	-3.677183	1.766064	-2.589418
H	1.806599	6.888367	-0.096734
H	0.394396	7.251413	-2.679385
H	-3.274897	6.749072	4.218928
H	6.520991	7.125117	6.486379
H	-4.424872	1.082175	-1.146478
H	-1.291468	-2.237418	-2.685412
H	2.59253	5.433656	-1.944883
H	5.564733	-3.205222	-4.659952

H	7.468891	7.271632	3.64051
H	-4.015171	3.731051	6.314505
H	-1.307255	0.777625	-5.129128
H	1.732558	4.564133	-3.207858
H	-0.346382	8.190418	-1.386526
H	3.470358	-3.392218	4.328908
H	-3.207375	0.50488	6.070345
H	-1.998738	3.257983	-5.298027
H	-0.66203	-0.900944	-3.656507
H	7.380993	0.158542	2.880187
H	-4.478286	4.728283	4.929728
H	0.918599	6.648574	5.400949
H	7.707254	-0.891565	5.733647
H	-2.011947	-1.602673	-5.439582
H	-0.651368	7.431541	5.45137
H	7.308169	8.843877	4.434056
H	-3.110548	-0.252694	4.460804
H	-1.940439	0.812543	-3.460344
H	5.122675	-1.898685	-5.789246
H	7.498236	2.496484	4.953629
H	3.23017	-3.73742	2.638913
H	1.888465	9.1292	5.28282
H	3.113638	7.207553	-3.419401
H	-1.206794	-3.167853	-5.446843
H	3.765258	6.061402	-4.57664
H	0.264722	9.526448	4.678728
H	-3.383019	2.861846	3.46923
H	1.387697	-4.406983	4.941136
H	-2.867429	3.091675	-3.803312
H	-2.443361	-2.424926	5.957871
H	7.149553	-2.367967	-6.830849
H	9.499546	3.014181	3.527581
H	6.273904	0.601757	4.294766

H	7.842324	-1.396059	-5.536569
H	8.405586	0.645757	5.217826
H	7.540767	4.130655	5.621904
H	1.442847	-5.538464	3.582102
H	-4.106858	-2.022539	5.562703
H	1.578057	1.832269	-6.902298
H	-3.507663	1.83987	4.883036
H	1.982458	-1.431803	5.751107
H	3.491729	-1.776906	4.909994
H	9.526132	4.664076	4.154911
H	0.783919	-0.656251	-7.003419
H	2.781991	1.369379	-8.122378
H	4.942432	3.492502	6.846011
H	3.67585	-3.491552	6.470256
H	4.143714	3.992293	9.797776
H	1.64858	-0.770095	-8.537837
H	4.431428	-2.216571	7.418922
H	3.605001	2.684889	7.705386
H	1.653313	4.020051	9.215695
H	-0.038145	2.27287	11.112383
H	5.521367	4.742969	8.97885
H	1.691681	2.107343	10.851698
H	-0.031474	4.354085	9.622708
H	0.597574	-7.686992	0.85227
H	0.42221	-6.255198	1.860006
H	-1.456633	-7.104919	-0.408881
H	-1.859334	-5.834142	0.773316
H	-4.664876	-1.130532	2.874252
H	-2.656363	-0.074931	2.290849
H	-2.094437	-1.760609	2.36995
H	-4.827986	-1.103413	1.125442
H	3.671992	-4.00896	-2.626791
H	3.995989	-6.514118	-1.515166

H	5.338545	-4.306957	-3.112041
H	5.680256	-5.975814	-1.534054
**B3-A**			
Au	0.026667	0.159251	-0.031944
Au	2.820731	0.148886	0.039666
Au	1.362774	2.626625	0.011425
Au	1.564428	-1.714185	-1.589407
Au	1.407318	0.417091	2.442192
Au	0.35206	4.977084	1.085865
Au	3.234706	4.649018	0.871988
Au	3.955419	2.4652	-1.121576
Au	-0.757124	2.264423	1.867798
Au	1.560603	1.103553	-2.322574
Au	1.770567	3.173085	2.737446
Au	5.66751	0.180881	0.02446
Au	4.22043	-2.208793	-0.091552
Au	4.246475	1.945695	1.688713
Au	1.598563	-2.06918	1.234023
Au	4.110527	-0.280489	-2.395613
Au	4.005163	-0.78938	2.392195
S	6.108447	-0.87202	-3.726773
Au	2.11313	5.881697	3.283074
Au	-0.482258	4.465361	3.712434
Au	4.286517	4.034473	3.62965
Au	0.187655	1.734945	4.575527
Au	3.228563	1.432246	4.356781
Au	1.942027	3.73683	5.450188
S	4.902679	6.146671	-0.026351
S	-0.460866	6.029347	-0.917964
S	5.809371	-1.623894	3.773795
S	-2.19349	-0.85549	-0.066362
S	7.878056	1.124289	0.262878
S	6.673352	4.285109	3.549606

S	0.655649	-3.346853	3.04735
S	0.174461	2.386258	-3.800583
S	-1.52341	0.167483	5.119618
S	2.932851	4.828906	7.371427
S	4.324831	-4.620076	-0.160787
Au	-1.083677	-2.882889	-0.468461
C	-2.849863	-1.014749	1.64819
Au	2.085679	-4.558858	-0.429091
C	5.02642	-5.402807	-1.678731
Au	4.917685	6.892707	2.350261
C	6.328095	5.061385	-0.421454
Au	7.157203	2.598669	1.944863
C	8.486398	1.981034	-1.266993
C	7.448418	3.790524	5.146305
Au	-0.092028	4.107578	-2.222825
C	0.632059	7.451406	-1.385337
C	-1.441976	1.539446	-4.052624
Au	-0.310372	-1.53183	3.98789
C	-1.68751	-0.492917	6.834047
C	1.767304	-4.449687	4.030951
Au	3.952759	6.223589	5.793374
C	4.272161	3.558391	7.543776
S	5.088964	7.787506	4.475029
C	4.622524	9.56561	4.552736
S	-0.170904	-4.99294	-0.831564
C	-0.875031	-6.147909	0.443814
S	-3.017366	2.456156	0.9306
S	1.644559	8.378136	3.335417
Au	-2.680339	4.945163	1.598476
C	-2.856136	2.654823	-0.888191
Au	-0.558205	7.464392	2.703761
C	1.15258	8.937424	5.025087
S	-2.818688	7.208018	2.087624

C	-3.677925	7.387227	3.713135
S	1.006973	-2.745379	-3.713267
S	4.325212	4.221943	-2.920656
Au	2.180831	-0.891274	-4.694882
C	-0.749385	-2.155518	-3.737396
Au	3.670539	2.297642	-4.336283
C	2.896486	5.370326	-3.01107
S	3.177395	0.765572	-5.970813
C	1.958349	1.386293	-7.229687
S	-2.122981	4.962715	5.499734
S	3.743595	0.008965	6.259479
Au	-0.921321	3.551393	6.732317
C	-3.23342	3.629709	4.820182
Au	1.834847	1.243036	7.29811
C	3.016335	-1.637706	5.854656
S	0.065393	2.19552	8.401664
C	0.46309	3.308578	9.817911
Au	6.875078	-2.020516	-1.83021
C	5.123985	-2.187188	-4.608447
S	7.762159	-3.256021	-0.049379
C	7.400436	5.849558	-1.161352
C	8.106195	6.748952	-0.170044
C	8.442568	8.080863	-0.459305
C	8.727874	6.215651	0.979464
C	8.559187	8.952457	0.635987
C	9.033311	8.453811	1.855627
C	9.088201	7.063712	2.034315
C	-3.705335	8.851844	4.153178
C	-4.212818	8.985048	5.569257
C	-5.551915	9.300784	5.838945
C	-3.419924	8.576738	6.650527
C	-5.920678	9.758549	7.10407
C	-5.088107	9.392713	8.176825

C	-3.959334	8.585566	7.940099
C	9.522267	-2.744284	0.175307
C	10.363754	-3.187795	-1.038349
C	11.211986	-2.119719	-1.705631
C	12.271841	-2.524184	-2.531487
C	11.060836	-0.743717	-1.499409
C	12.908348	-1.620954	-3.380821
C	12.639511	-0.252577	-3.214735
C	11.998434	0.151872	-2.017754
C	-4.214788	2.332181	-1.538187
C	-5.034299	3.583194	-1.729641
C	-6.059559	3.921257	-0.826587
C	-4.769849	4.517315	-2.746554
C	-5.250008	5.820834	-2.590028
C	-6.527669	5.976295	-2.040747
C	-6.903142	5.012493	-1.088157
C	7.485961	2.211557	-2.393461
C	8.056914	2.163651	-3.79472
C	7.205542	2.45901	-4.873315
C	9.392885	1.881867	-4.08984
C	7.516302	2.04194	-6.161208
C	9.783655	1.745418	-5.431562
C	8.785508	1.48893	-6.393806
C	1.787872	7.707924	-0.423508
C	2.549701	8.973905	-0.73277
C	2.086964	10.20527	-0.242954
C	3.644451	9.00079	-1.611569
C	2.771523	11.379711	-0.574809
C	4.110485	11.287674	-0.978111
C	4.542883	10.076319	-1.536154
C	5.78033	10.446264	4.074756
C	5.426019	11.558645	3.118212
C	6.408202	12.079096	2.255119

C	4.122111	12.050875	2.923201
C	6.238345	13.385197	1.779192
C	4.947896	13.882909	1.562524
C	3.891979	13.241008	2.2209
C	-2.338537	2.342414	-5.002626
C	-3.382356	1.454721	-5.637388
C	-3.064143	0.605063	-6.712161
C	-4.749763	1.605803	-5.354488
C	-4.098213	-0.101051	-7.336695
C	-5.23884	-0.401083	-6.57445
C	-5.579283	0.489369	-5.537746
C	-1.587453	-2.613516	-4.945812
C	-2.924887	-3.066095	-4.422447
C	-3.304589	-4.413798	-4.374878
C	-3.879189	-2.120199	-4.005547
C	-4.567658	-4.715409	-3.845857
C	-4.758834	-2.516067	-2.983867
C	-5.057593	-3.890591	-2.828334
C	3.199355	-4.571744	3.518269
C	3.802972	-5.912604	3.870302
C	3.640376	-6.499349	5.139121
C	4.61478	-6.60935	2.96349
C	4.463639	-7.562601	5.517677
C	4.912073	-8.407828	4.481151
C	4.82884	-7.977222	3.141975
C	0.590192	7.853767	5.943315
C	0.930768	7.940024	7.413746
C	0.210177	7.129905	8.311745
C	1.753858	8.922167	7.980097
C	0.675176	6.959997	9.616044
C	1.757401	7.716214	10.081352
C	2.27139	8.728419	9.269085
C	6.845365	-0.103355	3.948654

C	3.067601	6.317178	-4.232966
C	1.872533	6.17749	-5.146168
C	1.936751	5.356403	-6.284957
C	0.726802	6.980146	-5.020887
C	0.760337	5.055807	-6.984381
C	-0.474714	6.626514	-5.634979
C	-0.428845	5.740827	-6.720326
C	7.7717	-0.270027	5.174625
C	8.967977	-1.119843	4.831443
C	10.014613	-0.571594	4.070087
C	9.172939	-2.394473	5.373235
C	10.846323	-1.42072	3.340688
C	10.973357	-2.747481	3.784905
C	10.226325	-3.182365	4.886915
C	5.936954	-3.397517	-5.074783
C	5.107377	-4.182568	-6.063358
C	4.844745	-3.658772	-7.341178
C	4.411192	-5.340032	-5.683753
C	3.86884	-4.262622	-8.134989
C	3.61492	-5.626314	-7.965918
C	3.798891	-6.132718	-6.668321
C	-4.180725	3.008101	5.859647
C	-5.431207	2.445258	5.232281
C	-6.70442	2.905548	5.602058
C	-5.36462	1.707363	4.032982
C	-7.82171	2.141263	5.232946
C	-7.731885	1.240641	4.167156
C	-6.479675	0.994454	3.588204
C	-2.452501	-1.812185	6.899318
C	-3.487931	-1.938281	7.994823
C	-3.931176	-3.22914	8.343959
C	-3.75336	-0.946686	8.949176
C	-5.162301	-3.34387	8.997309

C	-5.815652	-2.169987	9.406633
C	-5.045496	-1.001121	9.516986
C	8.964306	3.605381	5.007486
C	9.83516	4.716043	5.548098
C	9.43334	5.583478	6.573172
C	11.037472	5.02794	4.886224
C	10.332194	6.525478	7.089079
C	11.991546	5.859515	5.469747
C	11.59138	6.704986	6.512788
C	0.803127	0.428069	-7.48611
C	1.016255	-0.681024	-8.49094
C	1.920642	-0.597207	-9.560861
C	0.467081	-1.951113	-8.247467
C	0.609743	-2.965788	-9.202031
C	1.808686	-1.445428	-10.660522
C	1.301858	-2.731033	-10.397108
C	3.63142	-2.697331	6.763384
C	2.979445	-2.844979	8.123917
C	1.646248	-2.483488	8.361562
C	3.768021	-3.196657	9.230071
C	3.180871	-3.463265	10.467664
C	1.057038	-2.774534	9.598142
C	1.78933	-3.441205	10.591049
C	5.440527	3.930764	8.444492
C	6.037593	2.732047	9.162812
C	7.364588	2.785202	9.623428
C	5.373169	1.500818	9.336367
C	7.793732	1.862939	10.580581
C	7.294433	0.55544	10.45783
C	6.084506	0.374931	9.77036
C	1.7513	2.883354	10.509575
C	1.994778	3.45652	11.885792
C	1.121823	4.358451	12.512868

C	2.921834	2.822844	12.733368
C	1.634781	5.156864	13.547306
C	3.430634	3.559217	13.811593
C	2.824642	4.770838	14.176808
C	0.17741	-6.685835	1.419051
C	0.10247	-8.138817	1.81657
C	-0.616866	-9.133593	1.13497
C	0.585695	-8.524031	3.088383
C	-0.261981	-10.471949	1.3908
C	0.907487	-9.84684	3.386689
C	0.32683	-10.85574	2.596653
C	-4.082813	-1.936335	1.672534
C	-4.076281	-2.883498	2.846057
C	-5.080838	-2.882938	3.821432
C	-3.048604	-3.83505	2.999619
C	-4.860493	-3.565837	5.023764
C	-4.130157	-4.760147	4.93778
C	-3.23788	-4.919846	3.857754
C	4.789762	-6.909361	-1.659219
C	5.835174	-7.763282	-0.976986
C	5.516971	-9.076549	-0.587036
C	7.08313	-7.289171	-0.540847
C	6.469607	-9.836086	0.103282
C	7.660328	-9.274648	0.57732
C	8.118613	-8.129504	-0.13991
H	-4.844422	6.608173	-3.232581
H	13.170169	0.482958	-3.824009
H	-6.283834	3.25144	0.006784
H	13.581504	-1.970709	-4.164527
H	1.159691	10.24469	0.335709
H	-6.876885	10.254318	7.273514
H	10.348887	-0.380196	-0.754907
H	9.234311	9.125078	2.692663

H	11.952625	1.21026	-1.750818
H	-6.221756	9.48062	4.991389
H	-5.38734	9.617808	9.203078
H	-5.436063	-0.14086	10.064679
H	12.495857	-3.591518	-2.618843
H	4.846816	-3.314559	9.100698
H	1.309756	-3.729508	11.528142
H	12.288566	7.431759	6.934444
H	7.923218	3.719054	9.49508
H	0.207099	-3.959483	-8.996844
H	0.214754	4.686971	12.000584
H	8.532178	10.031962	0.465507
H	3.447398	1.926801	12.391901
H	7.085197	13.883305	1.297862
H	3.800752	-3.802381	11.300582
H	7.773053	-0.279325	10.972895
H	8.7203	2.041279	11.131536
H	8.151829	8.495862	-1.430428
H	7.413066	11.643735	2.279542
H	2.37409	12.343959	-0.251426
H	1.064593	6.011327	13.916083
H	4.271117	3.171346	14.390079
H	3.247264	5.37684	14.97983
H	4.794563	14.836822	1.05354
H	-3.919229	4.349387	-3.411959
H	-7.089843	6.904889	-2.149786
H	3.56495	-3.768237	-9.061141
H	-7.814401	5.143363	-0.501017
H	8.647368	5.142059	1.197416
H	3.064435	-6.19778	-8.714638
H	2.869891	13.612567	2.117788
H	9.460353	6.639997	2.969223
H	-3.374267	8.203481	8.779143

H	-2.421734	8.169691	6.47918
H	5.247426	-2.681891	-7.620059
H	3.28595	11.602477	3.464304
H	9.950708	0.483859	3.783498
H	4.755639	12.170023	-0.975862
H	6.204978	2.856355	-4.67125
H	11.50162	-1.02936	2.560537
H	-2.058242	0.602638	-7.135639
H	10.150371	1.815197	-3.310143
H	-6.79485	3.637577	6.410002
H	3.973842	8.071329	-2.07797
H	3.447281	-7.134614	-6.413607
H	-8.799144	2.375053	5.660981
H	3.05901	-5.977202	5.905169
H	4.506173	-5.721043	-4.664499
H	5.5348	9.994663	-1.987285
H	-3.886704	-0.674679	-8.242209
H	-0.587929	6.485656	7.930794
H	11.693055	-3.420403	3.314313
H	2.85613	4.806083	-6.501423
H	6.762467	2.083557	-6.949561
H	-2.706123	-5.17663	-4.876412
H	4.515325	-7.887186	6.558123
H	10.829569	1.528258	-5.661581
H	-3.474949	-4.102515	7.870625
H	-8.611624	0.681838	3.843518
H	9.06301	1.145997	-7.393031
H	-3.7031	-1.057704	-4.187538
H	0.710453	7.756055	-4.251584
H	2.181158	9.713039	7.361794
H	2.468041	0.340661	-9.702478
H	4.788624	-6.168046	1.978329
H	8.473664	-2.798754	6.107866

H	-5.063646	2.394024	-4.663134
H	-4.388368	1.487345	3.5941
H	-5.920959	-1.19677	-6.875832
H	1.037346	-2.04261	7.57174
H	10.390495	-4.182406	5.292565
H	0.175846	6.263351	10.289665
H	-6.393209	0.335216	2.722044
H	8.467433	5.457174	7.066955
H	-4.933687	-5.743872	-3.921887
H	0.804359	4.370143	-7.834239
H	2.355823	-1.237865	-11.581547
H	5.327077	-9.389719	4.719273
H	-5.630156	-4.320552	9.134195
H	-3.194594	-0.010538	8.98036
H	11.314645	4.434634	4.009475
H	4.361281	1.347377	8.948757
H	-6.544314	0.419682	-5.033934
H	-5.281922	-1.763784	-2.390416
H	-0.00533	-2.569739	9.748888
H	5.207875	-8.594315	2.326496
H	-1.388145	7.179308	-5.407982
H	3.036641	9.406022	9.65218
H	2.156746	7.543994	11.082149
H	-1.335995	5.500939	-7.278447
H	-5.842402	-4.220271	-2.146562
H	10.013888	7.170082	7.91092
H	-0.124225	-2.127138	-7.347532
H	5.620488	-0.6111	9.702213
H	1.335656	-3.507922	-11.164192
H	12.969845	5.984127	5.00101
H	-6.826196	-2.204721	9.816688
H	-1.150477	-8.907107	0.208691
H	1.016562	-7.742534	3.72461

H	-0.684776	-11.24739	0.745336
H	1.41717	-10.085055	4.322227
H	0.512948	-11.910635	2.807582
H	-5.503007	-3.377199	5.885916
H	-2.223902	-3.845652	2.280269
H	-5.860042	-2.115869	3.777887
H	-2.575725	-5.787034	3.812175
H	-4.151506	-5.491281	5.748375
H	4.566451	-9.529307	-0.879132
H	7.360548	-6.263471	-0.806887
H	6.176886	-10.827113	0.461834
H	8.353195	-9.884613	1.162155
H	9.117063	-7.721599	0.032186
Au	6.688795	-2.471467	1.683313
H	-2.676651	9.248626	4.102707
H	6.952932	6.437913	-1.975303
H	6.713932	4.60872	0.501112
H	11.040185	-4.000788	-0.730334
H	-2.084509	1.934155	-1.194031
H	9.701272	-3.629615	-1.801708
H	9.26272	1.28294	-1.603985
H	2.454667	6.83739	-0.425085
H	9.535163	-1.653915	0.242053
H	-4.314016	9.451795	3.461579
H	8.10549	5.134969	-1.62169
H	8.980425	2.905431	-0.940362
H	6.552361	9.80874	3.615774
H	-4.695464	6.995802	3.5711
H	6.711575	1.426312	-2.327195
H	5.90836	4.258842	-1.032645
H	9.867208	-3.217236	1.099961
H	-2.512788	3.664428	-1.135021
H	6.94049	3.153691	-2.246073

H	-4.031718	1.860009	-2.516631
H	1.383659	7.783045	0.598404
H	0.993481	7.264987	-2.400478
H	-3.164373	6.764206	4.452131
H	6.266668	10.909821	4.951804
H	-4.754436	1.597134	-0.925276
H	-1.18128	-2.586525	-2.820312
H	2.973883	5.931973	-2.073854
H	4.306122	-2.520805	-3.960752
H	3.813507	9.65051	3.809989
H	-3.806351	4.108447	4.01026
H	-1.170061	0.589846	-4.533406
H	1.912558	4.857257	-2.99473
H	-0.051471	8.312698	-1.413518
H	3.818505	-3.747515	3.907
H	-0.688016	-0.573667	7.280055
H	-1.699088	2.798218	-5.775388
H	-0.750153	-1.070528	-3.604473
H	7.359386	0.183918	3.030704
H	-2.59771	2.862197	4.368229
H	0.982264	6.880508	5.592384
H	7.191141	-0.692714	6.00656
H	-1.714708	-1.782879	-5.65706
H	-0.499018	7.774826	5.802175
H	4.124342	9.76365	5.508784
H	-2.204268	0.313526	7.368299
H	-1.923234	1.308845	-3.095889
H	4.691116	-1.666786	-5.472217
H	6.958783	2.872954	5.497915
H	3.198644	-4.458569	2.424703
H	2.074997	9.354009	5.44683
H	3.148143	7.354273	-3.876012
H	-1.079299	-3.437126	-5.469521

H	3.994859	6.075871	-4.769082
H	0.443004	9.76344	4.885581
H	-3.634615	2.196851	6.372307
H	1.718236	-4.117912	5.075618
H	-2.810857	3.168142	-4.456531
H	-1.75211	-2.661726	6.922595
H	6.867186	-3.051454	-5.553888
H	9.242474	2.686536	5.555527
H	6.151374	0.641943	4.367906
H	6.226065	-4.013072	-4.210714
H	8.103842	0.737364	5.474621
H	7.178979	4.598148	5.837348
H	1.262866	-5.423203	3.970947
H	-3.009346	-1.941334	5.952324
H	1.571781	2.334817	-6.838283
H	-4.450228	3.746175	6.628747
H	1.92338	-1.584445	5.896899
H	3.284424	-1.823231	4.807488
H	9.21982	3.407698	3.95499
H	-0.03181	1.047811	-7.864838
H	2.560004	1.595276	-8.122557
H	4.610566	3.271491	6.540462
H	3.56606	-3.668694	6.240504
H	5.106534	4.666357	9.197527
H	0.465665	0.004633	-6.526712
H	4.707696	-2.503575	6.880444
H	3.724433	2.696146	7.94413
H	0.526676	4.337007	9.448606
H	1.788325	1.781946	10.568676
H	6.227304	4.437123	7.865566
H	2.599589	3.17095	9.86759
H	-0.412223	3.200893	10.472803
H	1.181427	-6.526983	0.984237

H	0.155051	-6.058371	2.321173
H	-1.328285	-6.941345	-0.162725
H	-1.679725	-5.621573	0.96764
H	-5.00481	-1.33819	1.677593
H	-3.075315	0.006521	1.976205
H	-2.046117	-1.406419	2.288222
H	-4.096496	-2.528131	0.743031
H	3.791838	-7.124405	-1.248567
H	6.087065	-5.125716	-1.70202
H	4.747714	-7.23971	-2.715276
H	4.53858	-4.941114	-2.544637
**B3-B**			
Au	-0.009555	0.087555	0.034255
Au	2.795429	0.081721	0.041651
Au	1.321352	2.547561	0.012312
Au	1.504507	-1.803958	-1.533797
Au	1.436986	0.380453	2.472335
Au	0.321365	4.916241	1.080037
Au	3.202884	4.589776	0.813122
Au	3.887543	2.392456	-1.165903
Au	-0.754116	2.207488	1.932698
Au	1.473412	1.002643	-2.293876
Au	1.786697	3.142008	2.72212
Au	5.632157	0.105015	-0.04006
Au	4.195578	-2.286023	-0.092084
Au	4.247076	1.889964	1.632351
Au	1.59415	-2.12442	1.303234
Au	4.021807	-0.391302	-2.407971
Au	4.034304	-0.824346	2.378711
S	6.027395	-0.922818	-3.662768
Au	2.119427	5.848518	3.223313
Au	-0.458774	4.432205	3.741111
Au	4.317645	4.028061	3.545064

Au	0.245365	1.708787	4.613963
Au	3.283197	1.417411	4.339234
Au	2.017748	3.741053	5.432098
S	4.793452	6.061712	-0.206525
S	-0.628605	6.006935	-0.956161
S	5.869232	-1.67233	3.690826
S	-2.251004	-0.870665	0.044432
S	7.861075	1.083765	0.092365
S	6.69043	4.095166	3.424727
S	0.471278	-3.482307	2.943265
S	-0.08575	2.142399	-3.751546
S	-1.244771	0.009329	5.485
S	3.193124	4.929934	7.275688
S	4.325736	-4.693104	0.050209
Au	-1.082454	-2.992408	-0.336358
C	-2.850181	-0.922088	1.788813
Au	2.003469	-4.685351	-0.340992
C	4.826193	-5.196922	-1.66861
Au	4.908697	6.841371	2.203899
C	6.344678	5.102024	-0.44434
Au	7.230828	2.48387	1.800786
C	8.379907	2.048244	-1.408829
C	7.47766	3.827333	5.063917
Au	-0.189832	3.989557	-2.203321
C	0.440486	7.406307	-1.563458
C	-1.746623	1.399398	-3.475414
Au	-0.275267	-1.600928	4.081143
C	-3.079285	-0.14918	5.353334
C	1.739512	-4.444035	3.913123
Au	3.996996	6.231303	5.726637
C	4.441837	3.579073	7.4539
S	5.132872	7.743306	4.320491
C	4.689632	9.535847	4.331339

S	-0.273113	-5.137899	-0.767709
C	-1.0032	-6.226106	0.53521
S	-3.009958	2.499056	1.095143
S	1.63	8.299232	3.159156
Au	-2.745056	4.858631	1.695006
C	-2.931688	2.468578	-0.710309
Au	-0.561004	7.471306	2.60446
C	1.091629	8.738127	4.889299
S	-2.800206	7.103316	2.18061
C	-4.015943	7.315218	3.546041
S	0.937999	-2.875612	-3.601584
S	4.206131	4.084734	-2.898832
Au	2.052575	-1.029084	-4.649657
C	-0.882442	-2.840868	-3.897071
Au	3.550896	2.204472	-4.367151
C	2.84757	5.319937	-2.927324
S	3.157909	0.568347	-5.955706
C	1.907855	1.202443	-7.157525
S	-2.194924	4.829345	5.379433
S	3.768288	-0.133356	6.133866
Au	-0.84794	3.558097	6.804611
C	-3.321165	3.406629	4.952777
Au	1.992076	1.274793	7.31077
C	2.677019	-1.585686	5.869802
S	0.337683	2.388732	8.408654
C	0.803223	3.495397	9.799805
Au	6.74382	-2.09903	-1.921602
C	5.47162	-2.258017	-4.837287
S	7.747804	-3.481318	-0.276217
C	7.308295	5.933954	-1.284382
C	7.993004	6.996734	-0.446773
C	8.54686	6.69206	0.807447
C	8.231326	8.276773	-0.958422

C	8.742835	7.70633	1.744508
C	8.747527	9.032727	1.288097
C	8.684761	9.281854	-0.090514
C	-4.196653	8.800507	3.890298
C	-4.068431	9.164493	5.357113
C	-3.443314	10.378729	5.697224
C	-4.16338	8.227991	6.395607
C	-3.588833	10.849005	7.008225
C	-4.387285	10.123973	7.904141
C	-4.574056	8.755837	7.637408
C	9.552718	-3.023887	-0.211516
C	9.838806	-1.729941	-0.995892
C	11.299222	-1.435738	-1.225846
C	11.853465	-0.24212	-0.744283
C	12.159471	-2.372504	-1.816416
C	13.135772	0.14562	-1.138492
C	13.813013	-0.611419	-2.094239
C	13.471951	-1.988306	-2.122777
C	-4.304306	2.334645	-1.35826
C	-4.883964	3.707372	-1.632152
C	-6.139078	4.091084	-1.152279
C	-4.091217	4.713872	-2.212763
C	-4.689173	5.815758	-2.813795
C	-5.993007	6.118928	-2.354205
C	-6.496915	5.439159	-1.205108
C	7.335595	2.235577	-2.506819
C	7.844254	2.131899	-3.932126
C	8.900072	1.289519	-4.306687
C	7.117097	2.77093	-4.947308
C	9.368513	1.278153	-5.622853
C	7.557065	2.727891	-6.271667
C	8.749827	2.07084	-6.593253
C	1.596979	7.72447	-0.624124

C	2.078193	9.167451	-0.679476
C	3.421461	9.421177	-0.334531
C	1.307665	10.270301	-1.066798
C	4.064987	10.569633	-0.775582
C	3.291272	11.674729	-1.160607
C	1.916358	11.504524	-1.344526
C	5.982543	10.303021	4.721329
C	5.813554	11.491857	5.645013
C	4.674709	11.75371	6.420485
C	6.817048	12.482427	5.653025
C	4.704188	12.805969	7.347674
C	5.639249	13.833438	7.256764
C	6.832185	13.531563	6.57045
C	-2.740471	1.709184	-4.589356
C	-3.870063	0.705119	-4.713269
C	-4.921857	0.971722	-5.60893
C	-3.854934	-0.58806	-4.173604
C	-6.140693	0.309851	-5.420196
C	-6.272765	-0.608654	-4.366476
C	-5.103461	-1.116654	-3.786024
C	-1.260946	-3.829137	-5.026808
C	-2.368116	-4.772248	-4.611856
C	-2.248956	-6.156574	-4.78844
C	-3.65842	-4.271466	-4.359942
C	-3.144587	-7.013209	-4.137827
C	-4.620588	-5.128139	-3.810254
C	-4.311334	-6.475379	-3.575532
C	3.212216	-4.189061	3.576047
C	4.079335	-5.418745	3.655572
C	3.783508	-6.580165	2.923914
C	5.104896	-5.518188	4.608737
C	4.67047	-7.661735	2.96125
C	5.846958	-7.596732	3.720828

C	6.148722	-6.425906	4.434993
C	0.877788	7.520854	5.782969
C	0.885931	7.74513	7.267758
C	-0.17136	7.265963	8.054716
C	1.906875	8.465986	7.902848
C	-0.033368	7.164839	9.439159
C	1.12643	7.654257	10.048338
C	1.974442	8.485395	9.299696
C	6.820398	-0.123838	4.026789
C	3.401072	6.579731	-3.588388
C	3.647201	6.384757	-5.067218
C	4.810196	6.866768	-5.681244
C	2.651241	5.869563	-5.913014
C	4.98164	6.738607	-7.065487
C	2.986673	5.354429	-7.161988
C	4.097032	5.936628	-7.800117
C	7.82458	-0.377716	5.155022
C	9.20269	-0.830719	4.71792
C	9.707168	-0.646513	3.419466
C	9.934557	-1.703369	5.545975
C	11.080278	-0.817752	3.197525
C	11.817288	-1.668658	4.025018
C	11.246446	-2.072723	5.238308
C	6.58228	-2.448391	-5.872845
C	7.701949	-3.28424	-5.300541
C	7.485453	-4.561704	-4.749482
C	9.056198	-2.927191	-5.460338
C	8.43796	-5.019956	-3.826076
C	9.784289	-4.672339	-3.959657
C	10.069586	-3.505611	-4.68844
C	-4.685567	3.50666	5.664789
C	-5.812445	3.632425	4.669632
C	-6.023204	2.6481	3.689046

C	-6.701111	4.717663	4.700812
C	-6.842922	2.932363	2.596044
C	-7.838621	3.90622	2.716676
C	-7.724605	4.839793	3.753027
C	-3.518752	-1.566047	5.706077
C	-3.53507	-1.933673	7.172489
C	-3.856039	-1.02165	8.185899
C	-3.22813	-3.250294	7.547274
C	-4.080965	-1.460253	9.492645
C	-3.70811	-2.757302	9.859167
C	-3.180405	-3.620136	8.892054
C	8.981908	4.120211	4.987703
C	9.358583	5.477029	5.55111
C	10.434151	6.190973	5.002416
C	8.754473	6.000185	6.705012
C	10.716778	7.493066	5.424437
C	9.025262	7.30137	7.131826
C	10.02581	8.047956	6.504567
C	1.535844	0.149305	-8.224944
C	0.055216	-0.129001	-8.303014
C	-0.628644	-0.71516	-7.228892
C	-0.713458	0.340464	-9.375744
C	-1.982699	-0.184752	-9.62275
C	-2.020903	-0.797518	-7.268727
C	-2.619876	-0.830337	-8.549422
C	3.09993	-2.717866	6.819814
C	2.342626	-2.560491	8.112369
C	2.977748	-2.177246	9.308118
C	0.988485	-2.920409	8.226352
C	0.240898	-2.312881	9.246548
C	2.252385	-1.661331	10.385475
C	0.861866	-1.841616	10.407139
C	5.687796	3.894903	8.257663

C	6.418908	2.621867	8.644988
C	5.763996	1.398556	8.857823
C	7.812327	2.656856	8.818625
C	6.513395	0.240088	9.081361
C	7.904175	0.2592	8.981845
C	8.52183	1.514319	9.196523
C	0.878186	2.70805	11.127254
C	2.191568	3.011929	11.796108
C	3.182582	2.038024	11.988161
C	2.417895	4.223074	12.479162
C	4.424816	2.448303	12.512222
C	3.686928	4.772532	12.578163
C	4.665013	3.783764	12.871556
C	-0.007036	-6.946157	1.433128
C	-0.714053	-7.665243	2.56224
C	-1.81308	-8.506752	2.32509
C	-0.282003	-7.509789	3.886068
C	-2.510585	-9.079938	3.390621
C	-0.957779	-8.110203	4.95008
C	-2.010652	-8.994223	4.6923
C	-4.335466	-1.294242	1.796672
C	-4.508958	-2.737696	1.399399
C	-5.134982	-3.10538	0.198574
C	-4.313347	-3.776422	2.330138
C	-4.831061	-4.36915	-0.32938
C	-4.466127	-5.400854	0.541511
C	-4.200357	-5.098373	1.884934
C	4.672108	-6.715004	-1.803239
C	3.41917	-7.175194	-2.517346
C	2.668598	-6.361036	-3.379364
C	2.816403	-8.390393	-2.127616
C	1.590744	-6.932545	-4.06742
C	0.892688	-8.014762	-3.542675

C	1.640586	-8.869119	-2.709735
H	-4.159388	6.464296	-3.510296
H	14.769692	-0.275362	-2.498673
H	-6.701913	3.391995	-0.527635
H	13.507619	1.133214	-0.854434
H	3.86843	8.632368	0.268661
H	-3.20403	11.832946	7.283566
H	11.817823	-3.386234	-2.028975
H	8.931812	9.853678	1.984045
H	14.131293	-2.696232	-2.631113
H	-3.015252	11.01575	4.920559
H	-4.668	10.543765	8.870942
H	-2.849359	-4.621971	9.1714
H	11.246467	0.415292	-0.116391
H	0.45931	-3.333587	7.361329
H	0.260643	-1.453835	11.23159
H	10.241451	9.066053	6.833277
H	4.673774	1.306227	8.794208
H	-2.489136	-0.031855	-10.57591
H	3.052118	1.024442	11.603351
H	8.940524	7.468329	2.790613
H	1.570527	4.925463	12.483066
H	3.828284	12.945948	7.988141
H	-0.846449	-2.414481	9.229375
H	8.481127	-0.663441	9.073268
H	5.986004	-0.715455	9.1316
H	8.540576	5.656597	1.159804
H	3.841657	11.050056	6.468372
H	5.137544	10.687061	-0.599717
H	5.207676	1.705941	12.678621
H	3.842021	5.82142	12.835105
H	5.670915	4.071611	13.186966
H	5.574466	14.702916	7.913256

H	-3.02467	4.510536	-2.406204
H	-6.467048	7.051079	-2.672885
H	8.182152	-5.88837	-3.210768
H	-7.397479	5.80165	-0.705751
H	7.980715	8.512826	-1.995101
H	10.542834	-5.144905	-3.333181
H	7.665575	14.237659	6.567791
H	8.834507	10.295981	-0.466909
H	-4.958933	8.093634	8.415484
H	-4.331356	7.169191	6.189913
H	6.471832	-4.972418	-4.703353
H	7.703396	12.316485	5.032213
H	9.138018	-0.083748	2.675351
H	3.769606	12.620284	-1.421914
H	9.410795	0.672734	-3.564694
H	11.507763	-0.497901	2.24362
H	-4.86415	1.830985	-6.281629
H	6.230359	3.353721	-4.689975
H	-5.34765	1.789833	3.63655
H	0.24094	10.160588	-1.27435
H	11.093178	-3.135851	-4.773813
H	-6.865876	2.233869	1.756075
H	2.936477	-6.601289	2.235142
H	9.27874	-1.998839	-5.996302
H	1.297732	12.349121	-1.654165
H	-7.009106	0.586048	-6.02143
H	-1.048125	6.839032	7.561968
H	12.853687	-1.914564	3.787469
H	5.576352	7.359013	-5.077506
H	10.242074	0.675318	-5.879945
H	-1.321995	-6.560234	-5.20987
H	4.44289	-8.565952	2.392757
H	7.002357	3.272732	-7.038665

H	-4.064363	0.021533	7.939098
H	-8.59171	4.033757	1.937496
H	9.133387	2.092228	-7.61513
H	-3.867829	-3.205623	-4.459799
H	1.695401	5.570486	-5.470268
H	2.67935	8.968234	7.317608
H	-0.07735	-0.955215	-6.311409
H	5.269182	-4.653455	5.26095
H	9.507309	-1.997151	6.508851
H	-2.974749	-0.958482	-3.646079
H	-6.580282	5.487079	5.467691
H	-7.250605	-1.009834	-4.095877
H	4.061683	-2.027975	9.286845
H	11.794918	-2.73323	5.912971
H	-0.806692	6.672595	10.032249
H	-8.440588	5.660066	3.835141
H	10.970325	5.775089	4.14558
H	-2.960786	-8.089031	-4.109724
H	5.850188	7.183258	-7.554412
H	-2.586331	-1.143457	-6.402991
H	6.570215	-8.413127	3.666501
H	-4.449754	-0.761723	10.245746
H	-2.920197	-3.963117	6.777793
H	7.964999	5.438633	7.21092
H	8.32976	3.619	8.772307
H	-5.143157	-1.962942	-3.097013
H	-5.605105	-4.73729	-3.531341
H	2.779955	-1.28359	11.263734
H	7.057777	-6.354336	5.034028
H	2.272306	4.751795	-7.725928
H	2.795591	9.009936	9.792871
H	1.267757	7.558253	11.126619
H	4.309233	5.716129	-8.848427

H	-5.029943	-7.120828	-3.06353
H	11.508761	8.060786	4.932036
H	-0.212975	0.919631	-10.158174
H	9.602275	1.576286	9.345651
H	-3.654336	-1.165842	-8.650568
H	8.487073	7.712759	7.987692
H	-3.788822	-3.079096	10.898819
H	-2.183366	-8.650095	1.307904
H	0.55975	-6.842141	4.083574
H	-3.38069	-9.707589	3.185083
H	-0.598732	-7.96479	5.970788
H	-2.523747	-9.497557	5.51377
H	-5.067485	-4.599129	-1.369947
H	-4.023626	-3.531956	3.356034
H	-5.480783	-2.314819	-0.475166
H	-3.955995	-5.895007	2.590937
H	-4.308458	-6.411668	0.161367
H	3.037855	-5.390276	-3.713825
H	3.347732	-9.027986	-1.414764
H	1.100878	-6.30972	-4.822006
H	-0.059245	-8.333878	-3.968731
H	1.217859	-9.814055	-2.360435
Au	6.692573	-2.527403	1.616918
H	-3.486593	9.409627	3.309254
H	6.780238	6.39399	-2.131928
H	6.760778	4.834077	0.531997
H	9.352289	-1.80107	-1.988231
H	-2.337467	1.56255	-0.880715
H	9.357018	-0.895997	-0.46999
H	9.234022	1.461879	-1.770763
H	2.452182	7.070759	-0.820855
H	9.733508	-2.688077	0.817178
H	-5.19702	9.122527	3.553695

H	8.061578	5.250638	-1.719276
H	8.773813	3.005794	-1.046371
H	6.518925	10.614504	3.812712
H	-4.929651	6.846465	3.16094
H	6.545694	1.474695	-2.370941
H	6.043462	4.178652	-0.948648
H	10.136383	-3.89378	-0.52416
H	-2.34673	3.320117	-1.074258
H	6.824815	3.198451	-2.39047
H	-4.211829	1.765594	-2.29612
H	1.268586	7.486211	0.403322
H	0.761241	7.172182	-2.584186
H	-3.66377	6.726121	4.39959
H	6.663642	9.59796	5.229905
H	-4.974972	1.756418	-0.706051
H	-1.338413	-3.129256	-2.936954
H	2.559147	5.483503	-1.88463
H	5.244166	-3.171057	-4.280018
H	4.414422	9.866981	3.327818
H	-3.46369	3.471109	3.864432
H	-1.546374	0.322531	-3.416644
H	1.984389	4.904062	-3.460526
H	-0.273894	8.236959	-1.611447
H	3.636298	-3.408617	4.214536
H	-3.492091	0.59514	6.046208
H	-2.20319	1.773427	-5.552792
H	-1.185354	-1.81359	-4.124279
H	7.335316	0.208488	3.123029
H	-2.781538	2.481539	5.161382
H	1.637329	6.764299	5.494395
H	7.405667	-1.080131	5.890413
H	-1.554506	-3.261376	-5.922894
H	-0.077589	7.050215	5.497055

H	3.980621	9.712008	5.141102
H	-3.398341	0.118904	4.337933
H	-2.106279	1.707386	-2.504465
H	4.550388	-1.886544	-5.305101
H	7.26113	2.809905	5.411806
H	3.269675	-3.795503	2.546861
H	1.895403	9.379217	5.278419
H	2.681852	7.404098	-3.437276
H	-0.370059	-4.414757	-5.305193
H	4.327075	6.886615	-3.081912
H	0.18518	9.349459	4.809622
H	-4.833015	2.608706	6.288627
H	1.524491	-4.271001	4.974729
H	-3.188064	2.702509	-4.425571
H	-2.888919	-2.280875	5.153878
H	6.145347	-2.951752	-6.754613
H	9.515557	3.337361	5.554664
H	6.079516	0.606946	4.374577
H	6.960477	-1.471964	-6.207105
H	7.950138	0.572763	5.707747
H	6.949258	4.532814	5.712596
H	1.480981	-5.49024	3.703612
H	-4.546294	-1.693166	5.314247
H	1.02781	1.533533	-6.592156
H	-4.698252	4.362307	6.355065
H	1.627764	-1.309304	6.017446
H	2.803761	-1.860507	4.815583
H	9.33169	4.038194	3.948737
H	2.059487	-0.794211	-7.998849
H	2.379738	2.088321	-7.603574
H	4.69307	3.19753	6.455523
H	2.857925	-3.685795	6.354693
H	5.398083	4.434805	9.177741

H	1.903415	0.476486	-9.207672
H	4.187132	-2.692445	6.981003
H	3.855057	2.793804	7.952256
H	1.749897	3.996567	9.561315
H	0.029096	2.989596	11.76764
H	6.362819	4.56929	7.709916
H	0.798436	1.629534	10.92808
H	0.024854	4.267032	9.809756
H	0.597893	-7.647127	0.832773
H	0.688211	-6.202794	1.84621
H	-1.617671	-6.932757	-0.039159
H	-1.676477	-5.605296	1.13661
H	-4.723392	-1.124304	2.815992
H	-2.681474	0.085505	2.188726
H	-2.244377	-1.634036	2.366536
H	-4.883713	-0.628065	1.113803
H	5.542584	-7.093123	-2.370977
H	5.877122	-4.895364	-1.764477
H	4.735471	-7.189777	-0.813363
H	4.239164	-4.62351	-2.389598
**B3-C**			
Au	0.046792	-0.033918	0.018152
Au	2.851363	-0.016973	0.048247
Au	1.357996	2.435922	-0.048528
Au	1.588558	-1.947922	-1.494641
Au	1.470875	0.325343	2.460573
Au	0.330201	4.819673	0.957466
Au	3.216356	4.511057	0.721156
Au	3.934846	2.276869	-1.212474
Au	-0.730388	2.121991	1.862284
Au	1.541465	0.840876	-2.318014
Au	1.796232	3.094927	2.650848
Au	5.688395	0.027346	-0.011139

Au	4.271174	-2.375761	-0.020825
Au	4.275583	1.838201	1.609422
Au	1.656325	-2.20269	1.33982
Au	4.100819	-0.533602	-2.38997
Au	4.07807	-0.86028	2.415025
S	6.041216	-1.105487	-3.726057
Au	2.103002	5.814667	3.093575
Au	-0.46787	4.389143	3.622415
Au	4.313102	4.020082	3.473939
Au	0.250994	1.691923	4.562058
Au	3.293233	1.4195	4.318402
Au	1.999423	3.758032	5.338818
S	4.771144	6.163509	-0.2276
S	-0.569596	5.674692	-1.100393
S	5.907938	-1.641053	3.769973
S	-2.249014	-0.920684	0.012938
S	7.936657	0.974914	0.219855
S	6.669417	4.265419	3.337615
S	0.500023	-3.549752	2.969577
S	0.017686	2.089086	-3.804025
S	-1.229097	-0.027716	5.330059
S	2.92657	4.990698	7.263531
S	4.452433	-4.851149	0.03136
Au	-0.963499	-3.08525	-0.292678
C	-2.715355	-0.963134	1.793996
Au	2.127409	-4.730958	-0.240956
C	4.890353	-5.057531	-1.762893
Au	4.895258	6.867179	2.136791
C	6.314361	5.282762	-0.70598
Au	7.202257	2.581235	1.815094
C	8.47689	1.885379	-1.308072
C	7.460909	3.915076	4.971194
Au	-0.189095	3.893643	-2.320207

C	0.176139	7.283622	-1.634634
C	-1.66333	1.329413	-3.937355
Au	-0.286472	-1.782728	4.115245
C	-1.132464	-0.563945	7.095086
C	1.73302	-4.453151	4.045928
Au	3.972891	6.288015	5.64535
C	4.357466	3.831771	7.583853
S	5.159783	7.721209	4.270104
C	4.869874	9.529975	4.489227
S	-0.162186	-5.25271	-0.623221
C	-0.740928	-6.227752	0.846577
S	-3.044091	2.35916	1.03327
S	1.508928	8.148499	3.199946
Au	-2.681414	4.746838	1.556052
C	-2.941749	2.431976	-0.795079
Au	-0.590136	7.39145	2.447061
C	1.039349	8.724805	4.896177
S	-2.818958	7.080731	1.741215
C	-3.735267	7.30348	3.341708
S	0.778721	-2.913753	-3.576917
S	4.069924	4.048976	-2.850937
Au	2.12732	-1.207489	-4.605887
C	-0.804524	-1.957973	-3.675215
Au	3.70877	2.073711	-4.39167
C	2.644512	5.142887	-3.149147
S	3.415394	0.312198	-5.861121
C	2.666467	0.961306	-7.413367
S	-2.181957	4.79505	5.305978
S	3.750164	0.012519	6.254011
Au	-0.931307	3.592317	6.700389
C	-3.46625	3.58595	4.709094
Au	1.913168	1.373385	7.248263
C	2.8464	-1.580673	6.056931

S	0.176614	2.411902	8.388439
C	0.63419	3.635595	9.697424
Au	6.880833	-2.215966	-1.7994
C	5.22512	-2.565649	-4.551222
S	7.834668	-3.453887	-0.020224
C	7.396868	6.274078	-1.12672
C	7.866248	7.163304	0.001504
C	7.7908	8.556097	-0.113752
C	8.573729	6.62868	1.086566
C	7.97884	9.365658	1.006737
C	8.330809	8.761528	2.217077
C	8.914106	7.477888	2.146188
C	-3.230205	8.494724	4.158162
C	-4.176038	8.941218	5.241254
C	-4.794669	10.205389	5.225555
C	-4.906685	8.011941	6.027458
C	-5.188121	10.659834	6.508185
C	-5.665487	9.763158	7.471675
C	-5.403641	8.390273	7.277327
C	9.563684	-2.801069	-0.122945
C	10.269051	-3.590949	-1.254509
C	10.804859	-2.786642	-2.426805
C	10.488536	-1.445195	-2.686672
C	11.346579	-3.492742	-3.521778
C	11.320881	-0.717796	-3.549551
C	11.910681	-1.40184	-4.61763
C	11.882463	-2.804683	-4.614348
C	-4.368994	2.367358	-1.34874
C	-5.022268	3.729883	-1.395138
C	-6.143305	4.004955	-0.600661
C	-4.395992	4.806211	-2.043225
C	-4.959372	6.080942	-1.994728
C	-6.254083	6.252494	-1.495251

C	-6.819765	5.222582	-0.736898
C	7.40904	2.071282	-2.387893
C	8.012847	2.363773	-3.736506
C	8.45292	1.338002	-4.584835
C	8.330159	3.680476	-4.111858
C	8.990685	1.657586	-5.837703
C	8.551473	4.010458	-5.446025
C	9.078101	2.990793	-6.259852
C	1.437733	7.726651	-0.909185
C	1.605353	9.228533	-0.798844
C	0.888944	10.122788	-1.608576
C	2.776495	9.723351	-0.202644
C	0.896262	11.486813	-1.287257
C	1.758726	11.934686	-0.283178
C	2.795157	11.082579	0.134961
C	5.042442	10.311784	3.1961
C	4.442937	11.699722	3.203462
C	3.247341	12.021839	3.85851
C	5.075162	12.720418	2.471578
C	2.695405	13.297276	3.707305
C	3.190429	14.186167	2.754585
C	4.562798	14.018819	2.437346
C	-2.695501	2.216268	-4.641099
C	-2.644161	2.169272	-6.149478
C	-2.757386	0.949694	-6.835558
C	-2.642634	3.345126	-6.908711
C	-2.789421	0.915449	-8.230021
C	-2.666343	2.100534	-8.965081
C	-2.466941	3.310009	-8.292773
C	-1.82243	-2.574139	-4.659652
C	-3.064137	-2.928782	-3.880489
C	-4.007856	-1.939972	-3.55528
C	-3.45219	-4.256975	-3.664547

C	-4.911355	-2.201536	-2.518481
C	-4.215841	-4.513845	-2.513813
C	-4.901725	-3.457608	-1.897101
C	3.213648	-4.143088	3.860345
C	4.121464	-5.360222	3.808132
C	3.69885	-6.697773	3.732377
C	5.504213	-5.182352	4.018097
C	4.669567	-7.706401	3.606032
C	6.024711	-7.482926	3.847585
C	6.461514	-6.140412	3.707898
C	0.551492	7.593541	5.797687
C	0.419911	7.90782	7.268183
C	1.38323	8.66177	7.952312
C	-0.518203	7.208399	8.03607
C	1.093129	9.205323	9.202093
C	-0.04236	8.712333	9.865492
C	-0.596109	7.492391	9.406601
C	6.838366	-0.063368	4.053898
C	2.89551	6.032659	-4.369336
C	4.072381	6.971674	-4.26394
C	3.953709	8.186988	-3.572129
C	5.192444	6.795835	-5.085552
C	5.088154	8.956562	-3.314832
C	6.342531	7.550777	-4.811766
C	6.326877	8.526088	-3.803483
C	7.795932	-0.238132	5.22596
C	9.077369	-0.979635	4.907664
C	9.690887	-1.764729	5.894839
C	9.674548	-0.93989	3.637642
C	10.971123	-2.288374	5.699047
C	11.550162	-2.258433	4.428052
C	10.909441	-1.550916	3.407486
C	6.166501	-3.363645	-5.476974

C	5.483012	-3.648592	-6.79623
C	5.417585	-2.645042	-7.772633
C	4.634306	-4.753743	-6.920223
C	4.919948	-2.951553	-9.038244
C	4.563498	-4.274549	-9.314944
C	4.082088	-4.999753	-8.191875
C	-4.744433	3.615041	5.54163
C	-5.750993	2.59289	5.059354
C	-6.06729	1.470156	5.838042
C	-6.119325	2.536836	3.702642
C	-7.247295	0.771344	5.561697
C	-7.781576	0.858664	4.26949
C	-7.145383	1.665712	3.316447
C	-2.20682	-1.604799	7.417771
C	-3.323291	-1.104695	8.310674
C	-4.159019	-2.03411	8.948249
C	-3.692152	0.244843	8.357178
C	-5.090978	-1.612285	9.896693
C	-5.159348	-0.257416	10.235738
C	-4.612534	0.663835	9.330514
C	8.822637	4.60755	5.093199
C	8.902355	5.612647	6.226895
C	7.790704	6.361723	6.64497
C	10.13517	5.854294	6.851376
C	7.92083	7.321088	7.651729
C	10.265596	6.837196	7.835254
C	9.180457	7.666287	8.143981
C	1.41226	1.808245	-7.190294
C	1.583639	3.309535	-7.375839
C	0.612274	4.15216	-6.811514
C	2.756235	3.92717	-7.833512
C	2.745581	5.285077	-8.176906
C	0.573784	5.500611	-7.173214

C	1.596595	6.048056	-7.958723
C	3.466085	-2.615034	7.008884
C	2.729729	-2.771577	8.317486
C	1.349237	-3.016668	8.358538
C	3.392523	-2.547767	9.530529
C	2.781558	-2.846613	10.74972
C	0.672668	-3.0334	9.579449
C	1.409255	-3.116535	10.769853
C	5.101656	4.06737	8.90668
C	5.562547	2.733703	9.442257
C	4.696836	1.932773	10.198296
C	6.811996	2.209411	9.0825
C	5.01787	0.604988	10.475528
C	6.290855	0.118426	10.155277
C	7.238724	0.97878	9.586449
C	0.435252	3.032398	11.100129
C	1.636864	3.186992	12.003467
C	2.087809	2.102449	12.769035
C	2.395817	4.367875	12.016742
C	3.224901	2.220138	13.57136
C	3.40038	4.550292	12.96958
C	3.829849	3.467477	13.743133
C	0.388887	-6.944853	1.593378
C	-0.011928	-8.270795	2.180092
C	0.601922	-9.472099	1.79303
C	-1.130009	-8.386042	3.038691
C	0.312104	-10.613024	2.557265
C	-1.517229	-9.603218	3.609434
C	-0.94066	-10.805004	3.127121
C	-4.173005	-1.418898	1.975168
C	-4.288424	-2.28589	3.199645
C	-3.788633	-3.602948	3.241104
C	-5.142564	-1.963755	4.272521

C	-3.871555	-4.265916	4.480726
C	-4.742483	-3.898651	5.511359
C	-5.048146	-2.500028	5.550209
C	5.028428	-6.508324	-2.230177
C	4.131307	-6.906317	-3.387512
C	4.531302	-7.952747	-4.239319
C	3.060741	-6.1251	-3.854751
C	3.5618	-8.582142	-5.029703
C	2.33942	-7.942481	-5.276444
C	2.083585	-6.708155	-4.671513
H	-4.444186	6.915155	-2.475633
H	12.438864	-0.8601	-5.404051
H	-6.567812	3.211561	0.019002
H	11.262052	0.372501	-3.568335
H	0.14882	9.751692	-2.320604
H	-5.411	11.723522	6.635865
H	11.436585	-4.58115	-3.47104
H	8.438084	9.35327	3.128405
H	12.301081	-3.363568	-5.453454
H	-4.507994	10.943138	4.467796
H	-6.051669	10.133233	8.423207
H	-4.814503	1.730506	9.452768
H	9.890026	-0.878051	-1.968352
H	4.447431	-2.265421	9.505331
H	0.89584	-3.284893	11.718435
H	9.288361	8.464326	8.880649
H	3.727931	2.32948	10.505946
H	3.62607	5.73421	-8.640037
H	1.564706	1.145541	12.701442
H	7.74945	10.431976	0.955751
H	2.102552	5.208669	11.382864
H	1.729629	13.501699	4.177717
H	3.353508	-2.811693	11.678539

H	6.575064	-0.898568	10.435599
H	4.289137	-0.035515	10.974086
H	7.415258	8.990217	-1.042491
H	2.750853	11.303971	4.511147
H	0.21358	12.175462	-1.78847
H	3.590465	1.353757	14.12546
H	3.904727	5.515056	13.049593
H	4.663275	3.584075	14.437597
H	2.691652	15.140906	2.575276
H	-3.432206	4.663903	-2.53664
H	-6.75502	7.218809	-1.573738
H	5.009814	-2.220264	-9.845458
H	-7.770448	5.378228	-0.223035
H	8.834681	5.569017	1.116883
H	4.282518	-4.58018	-10.324825
H	5.100923	14.795755	1.889275
H	9.420167	7.074865	3.025885
H	-5.786249	7.639329	7.971102
H	-4.685141	6.94737	5.885136
H	5.847988	-1.662049	-7.568875
H	6.065665	12.524593	2.051669
H	9.216264	-1.849772	6.8762
H	1.745786	12.975198	0.045878
H	8.314161	0.293192	-4.304314
H	11.464636	-2.827757	6.510362
H	-2.83085	0.01419	-6.276324
H	8.047505	4.483787	-3.423274
H	-5.606042	1.358786	6.822271
H	3.528954	9.03546	0.192992
H	3.497714	-5.910442	-8.347195
H	-7.64764	0.054488	6.280667
H	2.654906	-6.975855	3.586995
H	4.441955	-5.42623	-6.08307

H	3.600627	11.486466	0.746953
H	-2.920515	-0.037223	-8.746585
H	2.265956	9.013705	7.412374
H	12.524035	-2.71934	4.253513
H	3.00156	8.470463	-3.120658
H	9.359199	0.857922	-6.483575
H	-3.8914	-0.9219	-3.935313
H	4.314969	-8.739132	3.533631
H	8.583429	5.053983	-5.764071
H	-4.007573	-3.101329	8.768031
H	-8.657361	0.27	3.991696
H	9.426569	3.214509	-7.270437
H	-2.877388	-5.066658	-4.122086
H	5.225388	5.967743	-5.795955
H	-1.145534	6.438869	7.581318
H	-0.193434	3.714482	-6.226012
H	5.851958	-4.153341	4.150319
H	9.215101	-0.376431	2.823197
H	-2.599439	4.304895	-6.386699
H	-5.715324	3.25058	2.981354
H	-2.641582	2.066181	-10.055787
H	0.785397	-3.11429	7.426007
H	11.36048	-1.491033	2.414667
H	1.716456	9.984297	9.641861
H	-7.484397	1.652209	2.278515
H	6.790364	6.175229	6.24017
H	-5.570018	-1.411712	-2.152748
H	5.01284	9.837082	-2.673957
H	-0.258277	6.127549	-6.844222
H	6.731583	-8.314512	3.803055
H	-5.664324	-2.351328	10.460309
H	-3.191615	0.986733	7.732635
H	11.001059	5.246588	6.57639

H	7.507644	2.835374	8.515874
H	-2.333861	4.237955	-8.851024
H	-4.337526	-5.53548	-2.150154
H	-0.410941	-3.162129	9.60531
H	7.521684	-5.880079	3.740806
H	7.272442	7.33543	-5.342856
H	-1.326588	6.9682	10.027934
H	-0.305	9.086245	10.857383
H	7.240821	9.070807	-3.558574
H	-5.508377	-3.638643	-1.008259
H	7.03746	7.883137	7.963566
H	3.643468	3.342673	-8.07919
H	8.250976	0.624092	9.382312
H	1.555057	7.091798	-8.274083
H	11.241325	7.019039	8.290386
H	-5.806233	0.082834	11.045891
H	1.490691	-9.450891	1.15772
H	-1.628117	-7.471458	3.37427
H	0.920258	-11.503443	2.355239
H	-2.399476	-9.629466	4.253553
H	-1.243017	-11.775685	3.523077
H	-3.44427	-5.274883	4.52509
H	-5.607321	-0.971385	4.202852
H	-3.144693	-3.997231	2.450207
H	-5.582474	-2.045455	6.386433
H	-4.811236	-4.523329	6.405762
H	5.484535	-8.457383	-4.055769
H	2.799219	-5.185125	-3.363272
H	3.810583	-9.503299	-5.560907
H	1.585761	-8.419401	-5.905204
H	1.175528	-6.148321	-4.902334
Au	6.747054	-2.535509	1.732557
H	-2.26422	8.216608	4.614577

H	7.023971	6.883369	-1.961444
H	6.638583	4.637282	0.124068
H	11.118189	-4.150946	-0.828736
H	-2.360853	1.545491	-1.077238
H	9.588994	-4.365043	-1.646608
H	9.297683	1.26952	-1.702212
H	2.34165	7.287015	-1.351133
H	9.524633	-1.768301	-0.471598
H	-3.021242	9.345782	3.491053
H	8.248405	5.683157	-1.508926
H	8.900445	2.843809	-0.982857
H	4.55664	9.729055	2.390556
H	-4.785639	7.444957	3.051184
H	6.809077	1.152252	-2.453266
H	6.017405	4.656435	-1.552236
H	10.062318	-2.961995	0.836768
H	-2.39845	3.3253	-1.119185
H	6.714436	2.867195	-2.089235
H	-4.322735	1.951682	-2.369445
H	1.402417	7.322256	0.118711
H	0.303345	7.236977	-2.724606
H	-3.64595	6.375681	3.914455
H	6.104803	10.361591	2.926554
H	-4.9704	1.665808	-0.753266
H	-1.223368	-1.964452	-2.659284
H	2.525035	5.71639	-2.224453
H	4.764575	-3.191179	-3.783212
H	3.874534	9.662213	4.925679
H	-3.663636	3.882103	3.666366
H	-1.517554	0.418514	-4.526939
H	1.736785	4.543771	-3.306038
H	-0.643415	7.984091	-1.419331
H	3.59655	-3.458937	4.617427

H	-0.124613	-0.945352	7.292765
H	-2.60171	3.254151	-4.288324
H	-0.536105	-0.931257	-3.913709
H	7.377757	0.205436	3.140697
H	-3.015864	2.585465	4.692445
H	1.252403	6.749412	5.668527
H	7.278619	-0.711681	6.074377
H	-2.040046	-1.85491	-5.464005
H	-0.411458	7.218264	5.416757
H	5.601314	9.829786	5.253259
H	-1.244409	0.342793	7.700282
H	-1.993695	1.032381	-2.936012
H	4.422099	-2.119631	-5.149829
H	7.531457	2.829755	5.10324
H	3.338613	-3.607184	2.899952
H	1.956456	9.171642	5.303689
H	3.005411	5.388276	-5.253526
H	-1.39671	-3.470875	-5.132898
H	1.971375	6.617445	-4.522499
H	0.291834	9.518561	4.768114
H	-4.507283	3.429099	6.600561
H	1.411615	-4.290614	5.082881
H	-3.693929	1.871837	-4.319117
H	-1.729931	-2.462802	7.916833
H	7.084323	-2.782759	-5.65517
H	9.618349	3.857899	5.214773
H	6.082379	0.705005	4.261689
H	6.473105	-4.299731	-4.987832
H	8.070703	0.774511	5.578413
H	6.751267	4.287828	5.715817
H	1.524208	-5.50206	3.819395
H	-2.634244	-2.013092	6.486018
H	3.486158	1.497707	-7.901638

H	-5.195089	4.620759	5.491218
H	1.776887	-1.428517	6.228571
H	2.973967	-1.847016	5.00263
H	9.040459	5.130481	4.146911
H	0.996598	1.62077	-6.190323
H	2.451672	0.063065	-8.007718
H	5.019703	3.82669	6.713714
H	3.466731	-3.597726	6.505775
H	4.415757	4.529097	9.63426
H	0.630729	1.486051	-7.90137
H	4.522096	-2.371857	7.191765
H	3.880815	2.838655	7.627074
H	1.673088	3.93655	9.521249
H	-0.442826	3.506527	11.568775
H	5.944213	4.75907	8.762692
H	0.189187	1.964108	11.012504
H	0.009264	4.51665	9.515436
H	1.240246	-7.103734	0.914121
H	0.750367	-6.269639	2.377944
H	-1.451123	-6.949707	0.420104
H	-1.285697	-5.54714	1.509936
H	-4.834459	-0.544196	2.051821
H	-2.546471	0.03977	2.202008
H	-2.02525	-1.647103	2.304459
H	-4.479011	-1.99297	1.085485
H	6.076214	-6.722826	-2.490446
H	5.848592	-4.528698	-1.881141
H	4.791815	-7.184556	-1.389566
H	4.153702	-4.496482	-2.338853

References

[1] Baseggio, O.; Toffoli, D.; Fronzoni, G.; Stener, M.; Sementa, L.; Fortunelli, A. Extension of the Time-Dependent Density Functional Complex Polarizability Algorithm to Circular Dichroism: Implementation and Applications to Ag_8 and $Au_{38}(SC_2H_4C_6H_5)_{24}$. *J. Phys. Chem. C* **2016**, *120*, 24335-24345.

[2] Baseggio, O.; de Vetta, M.; Fronzoni, G.; Toffoli, D.; Stener, M.; Sementa, L.; Fortunelli, A. Time-dependent density-functional study of the photoabsorption spectrum of Au25(SC2H4C6H5)18 anion: Validation of the computational protocol. *Int. J. Quantum Chem.* **2018**, *118*, e25769.
