# Supplementary material for: The Conformational Dynamics of the Ligands Determines the Electronic Circular Dichroism of the Chiral Au38(SC2H4Ph)24 Cluster
Source: J Phys Chem Lett. 2023 Feb 14;14(7):1941–8. doi: 10.1021/acs.jpclett.2c03923 (PMC9940292; doi:10.1021/acs.jpclett.2c03923)
Supplement: Supplementary file 2 — jz2c03923_si_002.pdf [file jz2c03923_si_002.pdf]

jz-2022-03923g.R1

Name: Peer Review Information for "The Conformational Dynamics of the Ligands Determines the Electronic Circular Dichroism of the Chiral $Au_{38}(SC_2H_4Ph)_{24}$ Cluster"

First Round of Reviewer Comments

Reviewer: 1

Comments to the Author

The authors present a nice and really interesting result about the influence of the ligand shell of chiral clusters on the CD spectra.The CDs are usually qualitatively all similar, but the intensities vary quite a bit in the higher energy region, and so do the energies of the clusters. It is not often that the calculations are done with the full ligand shell, so one was not aware of this phenomenon. Therefore, I recommend the manuscript for publication after

- I would recommend changing the colours in Figure 1 (yellow-orange) to make it clearer.

- Could the authors clarify why there are no lower energy structures for B2 and B3 in Figure 3. It is mentioned that the original 19 structures were reduced to 12 that really had conformational and spectral differences, is this the reason?

-It might be interesting to get an idea of the computational cost/general effort of the whole new approach compared to just optimising the crystal structure at the HDA-B3LYP/TZP level and then calculating the ECD.

Author's Response to Peer Review Comments:

Trieste, February 3rd 2023

Your Ref. ID jz-2022-03923g

Dear Prof. Editor,

please consider the revised version of the manuscript entitled:

"The Conformational Dynamics of the ligands determines the Electronic Circular Dichroism of the Chiral $Au_{38}(SC_2H_4Ph)_{24}$ Cluster"

Authors:
M. Monti, G. Brancolini, E. Coccia, D. Toffoli, A. Fortunelli*, S. Corni, M. Aschi* and M. Stener*:

*corresponding authors, e-mail: stener@units.it, alessandro.fortunelli@cnr.it, massimiliano.aschi@univaq.it.

Which has been amended accordingly to the comments of the referee:

Referee: I would recommend changing the colours in Figure 1 (yellow-orange) to make it clearer.
Authors: we have improved Figure 1 using balls instead of sticks for sulphur atoms. The caption has been rephrased accordingly.

Referee: Could the authors clarify why there are no lower energy structures for B2 and B3 in Figure 3. It is mentioned that the original 19 structures were reduced to 12 that really had conformational and spectral differences, is this the reason?
Authors: We have inserted in the text the following three sentences (page 10) in order to clarify the point raised by the referee: "*The three basins differ in terms of dimensions and energy range, indeed while B2 and B3 are quite small and associated to higher $\Delta G°$ values (> 2.0 kJ/mol), B1 is very extended and contains the most probable conformers. Such result shows the possibility of having different, more or less probable, ligand conformations in different region of the ES. Therefore the reduction from 19 to 12 structures thanks to the RMSD analysis does not interfere on the absence of low energy structures in the B2 and B3 basins.* "

Referee: It might be interesting to get an idea of the computational cost/general effort of the whole new approach compared to just optimising the crystal structure at the HDA-B3LYP/TZP level and then calculating the ECD.
Authors: We have inserted in the text the following four sentences (page 14) in order to give a complete assessment of the computational effort needed for the B3LYP-HDA calculations: "*The present B3LYP-HDA calculation has been rather demanding, 6 days using 144 cores on the Galileo supercomputer of CINECA (Bologna, Italy) consisting of several nodes each with 2 x CPU Intel CascadeLake 8260, with 24 cores each. Instead, for each structure calculated at the LB94 level, the SCF required 5 hours at 144 cores on the Galileo supercomputer, while the polTDDFT section took 15 hours with 24 CPU on a HP ProLiant DL580 Gen10 server. The latter consists in 4 processors each with 18 cores Intel® Xeon® Gold 6140 CPU @ 2.30GHz, in total*

72 cores and 728 GB of RAM. We are working on a new implementation of the B3LYP-HDA scheme based on the Resolution of the Identity instead of the presently implemented numerical integration, which is very promising in terms of numerical efficiency."

Besides the scientific improvement suggested by the referee we have also implemented the following non-scientific changes suggested by the editor:

1. TOC Graphic: we have provide a TOC image just above the abstract (page 2).

2. Title: we have capitalized the title words.

3. Headers: we have removed the section heading(s)

4. References: we have avoided URLs.

5. References: we have fixed the style.

We hope that now you will find the revised manuscript acceptable for publication in *The Journal of Physical Chemistry Letters*.

with best regards, I remain
yours sincerely

M. Stener (corresponding author)

Prof. Mauro Stener
Dipartimento di Scienze Chimiche e Farmaceutiche
Università di Trieste
Via L. Giorgieri 1
I-34127 TRIESTE
ITALY
Tel.: ++39-040-558 3949
FAX: ++39-040-558 3903
e-mail: stener@units.it
